# Maintenance Therapy for Children and Adolescents with Asthma: Guidelines and Recommendations from the Emilia-Romagna Asthma (ERA) Study Group

**DOI:** 10.3390/jcm12175467

**Published:** 2023-08-23

**Authors:** Valentina Fainardi, Carlo Caffarelli, Michela Deolmi, Giulia Zambelli, Elisabetta Palazzolo, Sara Scavone, Barbara Maria Bergamini, Luca Bertelli, Loretta Biserna, Paolo Bottau, Elena Corinaldesi, Nicoletta De Paulis, Emanuela Di Palmo, Arianna Dondi, Marcella Gallucci, Battista Guidi, Francesca Lombardi, Maria Sole Magistrali, Elisabetta Marastoni, Silvia Pastorelli, Alessandra Piccorossi, Maurizio Poloni, Sylvie Tagliati, Francesca Vaienti, Giuseppe Gregori, Roberto Sacchetti, Francesco Antodaro, Andrea Bergomi, Lamberto Reggiani, Alessandro De Fanti, Federico Marchetti, Roberto Grandinetti, Nicole Mussi, Giampaolo Ricci, Susanna Esposito

**Affiliations:** 1 Pediatric Clinic, Department of Medicine and Surgery, University of Parma, 43126 Parma, Italy; valentina.fainardi@unipr.it (V.F.); carlo.caffarelli@unipr.it (C.C.); mdeolmi@ao.pr.it (M.D.); zambelligi@gmail.com (G.Z.); elisabetta.palazzolo@unipr.it (E.P.); sara.scavone92@gmail.com (S.S.); roberto.grandinetti@unipr.it (R.G.); nicole.mussi1@gmail.com (N.M.); 2 Paediatric Unit, Department of Medical and Surgical Sciences of Mothers, Children and Adults, University of Modena and Reggio Emilia, 41125 Modena, Italy; barbaramaria.bergamini@unimore.it; 3 Pediatric Clinic, Scientific Institute for Research and Healthcare (IRCCS) Azienda Ospedaliero-Universitaria di Bologna, 40138 Bologna, Italy; luca.bertelli@aosp.bo.it (L.B.); emanuela.dipalmo@gmail.com (E.D.P.); arianna.dondi@aosp.bo.it (A.D.); marcella.gallucci@aosp.bo.it (M.G.); giampaolo.ricci@unibo.it (G.R.); 4 Paediatrics and Neonatology Unit, Ravenna Hospital, Azienda Unità Sanitaria Locale (AUSL) Romagna, 48121 Ravenna, Italy; lorettabiserna@libero.it (L.B.); federico.marchetti@auslromagna.it (F.M.); 5 Paediatrics Unit, Imola Hospital, 40026 Imola, Italy; p.bottau@ausl.imola.bo.it; 6 Paediatric Unit, Carpi Hospital, 41012 Carpi, Italy; elcorinaldesi@yahoo.it; 7 Paediatrics and Neonatology Unit, Guglielmo da Saliceto Hospital, 29121 Piacenza, Italy; n.depaulis@ausl.pc.it (N.D.P.);; 8 Hospital and Territorial Paediatrics Unit, 41026 Pavullo, Italy; b.guidi@ausl.mo.it; 9 Paediatrics Unit, Maggiore Hospital, 40133 Bologna, Italy; francesca.lombardi@ausl.bologna.it; 10 Paediatrics Unit, Santa Maria Nuova Hospital, Azienda Unità Sanitaria Locale (AUSL)-Scientific Institute for Research and Healthcare (IRCCS) of Reggio Emilia, 42123 Reggio Emilia, Italyalessandro.defanti@ausl.re.it (A.D.F.); 11 Paediatrics Unit, Sassuolo Hospital, 41049 Sassuolo, Italy; s.pastorelli@ospedale.sassuolo.it; 12 Paediatrics and Paediatric Intensive Care Unit, Cesena Hospital, Azienda Unità Sanitaria Locale (AUSL) Romagna, 47521 Cesena, Italy; 13 Paediatrics Unit, Rimini Hospital, Azienda Unità Sanitaria Locale (AUSL) Romagna, 47921 Rimini, Italy; polonimaurizio@gmail.com; 14 Paediatric Clinic, Ferrara Hospital, 44124 Ferrara, Italy; sylvie.tagliati@unife.it; 15 Paediatrics Unit, G.B. Morgagni Pierantoni Hospital, Azienda Unità Sanitaria Locale (AUSL) Romagna, 47121 Forlì, Italy; francesca.vaienti@auslromagna.it; 16 Primary Care Pediatricians, Azienda Unità Sanitaria Locale (AUSL) Piacenza, 29121 Piacenza, Italy; g.greg@agonet.it (G.G.); robertosacchetti16@gmail.com (R.S.); 17 Primary Care Pediatricians, Azienda Unità Sanitaria Locale (AUSL) Modena, 41125 Modena, Italy; francesco.antodaro@gmail.com (F.A.);; 18 Primary Care Pediatricians, Azienda Unità Sanitaria Locale (AUSL) Imola, 40026 Imola, Italy

**Keywords:** asthma, inhaled corticosteroid, long-acting beta agonist, oral corticosteroids, pediatric pulmonology, short-acting beta agonist

## Abstract

Asthma is the most frequent chronic disease of childhood, affecting up to 20% of children worldwide. The main guidelines on asthma maintenance therapy in pediatrics suggest different approaches and describe different stages of asthma to determine the most appropriate treatment. This project aims to summarize the most recent evidence regarding maintenance therapy for asthma in children and adolescents. A multidisciplinary panel of experts was asked clinical questions regarding the treatment of children and adolescents with asthma. Overall, 10 clinical questions were addressed, and the search strategy included accessing electronic databases and a manual search of gray literature published in the last 25 years. After data extraction and narrative synthesis of results, recommendations were developed using the Grading of Recommendations, Assessment, Development, and Evaluations (GRADE) methodology. Results showed that the choice of medication depends on the severity of the child’s asthma, phenotype, age, preference, and individual factors. In addition to medications, the identification of comorbidities and modifiable factors is crucial to obtaining good control. Asthma in children is heterogeneous, and its evolution varies over time. Since most recommendations for asthma management in childhood are extrapolated from clinical studies performed in adults, more clinical trials specifically designed for young children should be conducted.

## 1. Introduction

Asthma is the most frequent chronic disease of childhood, affecting up to 20% of children worldwide [1]. Asthma symptoms include wheezing, dyspnea, chest tightness, and coughing, usually associated with reversible airway obstruction [2]. Asthma exacerbations result in missed school, progressive loss of lung function, impaired activity, and impaired sleep [3,4]. Daily maintenance therapy aims to avoid exacerbations and obtain good day-to-day symptom control. Most guidelines and recommendations suggest daily inhaled corticosteroids (ICS) for the treatment of patients with persistent asthma to decrease airway inflammation, control symptoms, and reduce the risk of exacerbations [2,5,6]. When ICS alone cannot control asthma symptoms, the addition of a long-acting beta-agonist (LABA) and/or leukotriene receptor antagonists (LTRA) can be considered [7,8].

Indications for asthma maintenance therapy mainly come from three documents: (1) the National Asthma Education and Prevention Program Coordinating Committee Expert Panel Working Group (NAEPP; last update on asthma in 2020) that review certain topics by GRADE analysis [6]; (2) the Global Initiative for Asthma (GINA) Report, an international collaboration launched by the United States National Heart, Lung and Blood Institute, the National Institutes of Health and the World Health Organization that is updated every year with the newest publications on the topic but does not use GRADE methodology [2]; (3) the National Institute for Health and Care Excellence (NICE) guideline produced by the joint initiative of the British Thoracic Society (BTS) and the Scottish Intercollegiate Guidelines Network (SIGN) guidelines on asthma that, however, are expected to be updated in 2024. NAEPP and GINA have different but complementary approaches, and both describe different steps of asthma to decide the most appropriate treatment both in children and adolescents [2,6].

This project aims to summarize the most recent evidence regarding maintenance therapy for asthma in children and adolescents (i.e., 6–17 years). We did consider the management of preschool wheezing because we have already developed guidelines on this topic [9,10].

## 2. Materials and Methods

We set up a multidisciplinary panel of experts that included all the Heads of the Pediatric Units in Emilia-Romagna Region, Italy; the Heads of the outpatient clinics for pulmonology and allergology; a sample of primary care pediatricians (identified in each province based on the number of the pediatric population according to ISTAT 2018 data); and a patients’ Association (Respiro Libero, Parma, Italy). This study group (named Emilia-Romagna Asthma Study Group and described in detail in a previous publication on the management of children with acute asthma attacks [11]) included members with previous experience in the development of documents and recommendations with the GRADE method [12,13].

The aim was to address ten different key questions regarding the treatment of children and adolescents with asthma. Clinical questions have been formulated by the expert panel using the PICO format (Patients, Intervention, Comparison, Outcomes), and systematic reviews have been conducted on PubMed to answer these specific questions with the aim of formulating recommendations. Each subgroup (at least two people) formulated a search strategy and reviewed the retrieved references for relevant papers from April 1997 to April 2023. Prospective or retrospective cohort and case-control studies were included. Included studies investigated children aged >6 years of age. Letters, comments, editorials, and case reports were excluded. Only full manuscripts published in the English language were included. The quality of the evidence has been assessed for each individual outcome. Search strategies, extended evidence tables, and individual outcomes are available in Supplementary Material File S1. Questions were:

1. In children with mild asthma and occasional symptoms, is short-acting beta2agonists (SABA) combined with inhaled corticosteroids or as-needed ICS-formoterol preferred to SABA alone?
2. In children with asthma, is daily therapy with ICS more effective than daily LTRA?
3. In children with uncontrolled asthma symptoms despite daily ICS, is increasing the dose of ICS more effective than adding LABA or LTRA?
4. In children with uncontrolled asthma symptoms despite daily therapy, what is the preferred option between increasing the therapy or assessing modifiable factors (adherence, inhalation technique, exposure to allergens)?
5. In children with asthma, is a metered dose inhaler (MDI) preferred to a dry powder inhaler (DPI)?
6. Which patients with asthma can benefit from immunotherapy?
7. In children with uncontrolled asthma symptoms despite daily medium-dose ICS combined with LABA, is increasing the dose of ICS more effective than adding the long-acting muscarinic antagonist (LAMA) tiotropium?
8. Considering the biologics for severe asthma, what are the differences between omalizumab, mepolizumab, and dupilumab?
9. In children with asthma, does vitamin D supplementation help with asthma control?
10. In children with asthma, does flu vaccination help with asthma control?

Recommendations are graded as strong or weak after considering the quality of the evidence, the balance of desirable and undesirable consequences of the compared management options, the assumptions about the relative importance of outcomes, the implications for resource use, and the acceptability and feasibility of implementation. The panel then decided on the strength of the recommendations. A dedicated voting process (collection of voting forms through individual email messages) was developed for the present guidelines, and an online meeting with the participation of the full voting panel was organized. More specifically, voting panel members were provided with the results of the various literature searches, the evidence summaries, the proposed recommendations, and the related GRADE tables. Each voting member was then allowed to individually vote in favor or against each recommendation, propose possible modifications, and judge each recommendation as strong or weak according to GRADE rules. For recommendations with an agreement of <75%, further voting rounds were conducted after implementing dedicated amendments based on the provided comments. After reaching an agreement of ≥75% for all recommendations, all the authors reviewed and approved the final manuscript and Supplementary Material File S1.

## 3. Results

### 3.1. PICO Question 1. In Children with Infrequent Symptoms, Is SABA Combined with ICS or As-Needed ICS-Formoterol Preferred to SABA Alone?

#### 3.1.1. Executive Summary

Children and adolescents with infrequent symptoms (symptoms less than twice a month and no exacerbations in the last 12 months when considering children aged >12 years) should start with the lowest level of asthma treatment, referred to as step 1.

While NAEPP guidelines suggest SABA as the first step of asthma treatment, the last 2022 GINA report recommends that SABA has to always be associated with low-dose ICS (in patients aged 12 years and older, low-dose ICS-formoterol taken as needed for relief of symptoms is also considered an alternative) since severe attacks can also present in patients with “mild asthma” [2,6]. Formoterol is a selective beta 2 agonist that has a rapid onset of action after inhalation; its effect lasts 12 h while the effects of SABA last 3–4 h. On the other hand, the effects of LABA appear slowly after inhalation, so LABA is not useful in acute attacks of bronchospasm.

Mild asthma is a definition of asthma severity that implies sporadic symptoms; however, that can sometimes be misleading both for the patient and the clinician since 30% of severe exacerbations and deaths occur in mild asthma patients with infrequent symptoms [14,15,16]. SABA is effective for the quick relief of symptoms; however, one of the major risks associated with the use of SABA alone is loss of asthma control. Overreliance on SABA can mask underlying inflammation and airway narrowing, leading to worsening symptoms, increased use of rescue medication, and an increased risk of exacerbations. Regular use of SABA can also desensitize the airway beta2-receptors, reducing the bronchodilator response and making it less effective over time [17]. In adults, the use of three or more canisters per year has been associated with an increased risk of emergency visits or hospitalizations the and use of eleven or more canisters per year has been associated with an increased risk of death [18,19].

The double-blind trial conducted by O’Byrne et al. showed that budesonide–formoterol used as needed was more effective than SABA alone in patients 12 years of age and older with mild asthma [20]. Patients were randomized to one of these three regimens: twice-daily placebo plus terbutaline used as needed, (terbutaline group), twice-daily placebo plus budesonide–formoterol used as needed, or twice-daily budesonide plus terbutaline used as needed. Results showed that budesonide–formoterol used as needed was superior to terbutaline used as needed for symptom control [34.4% vs. 31.1%; odds ratio, 1.14; 95% confidence interval (CI), 1.00 to 1.30; *p* = 0.046] but inferior to budesonide maintenance therapy (34.4% and 44.4%, respectively; odds ratio, 0.64; 95% CI, 0.57 to 0.73) [20]. This and other studies showed that exacerbation rates with the two budesonide-containing regimens were similar and lower than with terbutaline [20,21,22]. Similar results were reported by Bateman et al. [23].

There is only one study on young children (4 to 11 years old) that investigated the role of ICS with salbutamol as rescue medication and demonstrated that this approach was more effective than salbutamol alone in preventing exacerbations [21].

For children with mild asthma with symptoms twice a month or more (step 2), two approaches are recommended by the GINA report: (1) low-dose ICS-formoterol, taken as needed for relief of symptoms and if needed before exercise (in children aged >12 years) or (2) daily low-dose maintenance ICS and as-needed SABA for quick-relief therapy (in children aged 6–11 years and in children aged >12 years who prefer this regimen) [2]. The last NAEPP Expert Panel recommended for patients in step 2 aged >12 years two similar approaches: (1) daily low-dose ICS and as-needed SABA or (2) as-needed ICS and SABA used at the same time for symptom relief (NAEPP) [6].

The use of as-needed ICS combined with SABA had first been suggested in the paper by Papi et al. [22], where in patients with mild asthma, the use of ICS (beclomethasone) and salbutamol driven by symptoms was as effective as daily use of inhaled beclomethasone and was associated with a lower 6-month cumulative dose of ICS. When considering the as-needed low-dose ICS-formoterol approach compared to maintenance ICS, the risk of exacerbations was non-inferior in two double-blind randomized controlled trials (RCTs) [20,23] and superior in two open-label RCTs [24,25], with a significantly lower intake of the ICS dose in the as-needed ICS-formoterol regimen. However, symptom control was inferior in the budesonide–formoterol used aa s needed group compared to budesonide maintenance therapy (34.4% and 44.4%, respectively; odds ratio, 0.64; 95% CI, 0.57 to 0.73) [20].

Regular ICS with as-needed SABA is a further possible option for mild asthma; however, the likelihood of poor adherence in children with infrequent symptoms, particularly adolescents, should be considered. The post hoc pooled analysis of Symbicort Given as-needed in Mild Asthma (SYGMA) 1 and 2 suggested this approach in this age group (n = 889). The study assessed the efficacy and safety of as needed budesonide-formoterol (BUD-FORM) in adolescents. Patients 12–18 years old were randomized to twice-daily placebo + as needed BUD-FORM, twice-daily BUD + as needed terbutaline (BUD maintenance), or twice-daily placebo + as needed terbutaline (SYGMA 1 only). In SYGMA 1, the annualized rate of severe exacerbations in adolescents was 77% lower with as needed BUD-FORM versus as needed terbutaline (0.04 vs. 0.17; RR 0.23; 95% CI, 0.09 to 0.65; *p* = 0.005). The severe exacerbation rate was similar with as needed BUD-FORM and BUD maintenance (pooled analysis: 0.08 vs. 0.07/y; *p* = 0.634), suggesting this approach as an alternative treatment for adolescents with mild asthma [26]. One of the two SYGMA studies (SYGMA 1) was a 52-week, double blind, RCT on patients aged 12 years or older (n = 5721). A post hoc analysis of 3849 patients randomized to different treatments demonstrated that, as needed, budesonide-formoterol reduced the short-term risk of severe exacerbations after a single day of more than two as needed inhalations, even when the use of the medication is infrequent [27].

A recent RCT analyzed the cost-effectiveness between the as-needed use of SABA alone versus as-needed use of SABA plus ICS in pediatric patients with mild intermittent asthma (Step 1). The study considered as its primary outcome the first course of prednisone for asthma exacerbations. Compared with the use of SABA alone, the as-needed use of SABA plus ICS was associated with lower overall treatment costs and less need for prednisone [28]. In a probabilistic Markov cohort model with a 70-year time horizon, as-needed budesonide/formoterol was demonstrated to be a more cost-effective option for the treatment of mild asthma compared to daily ICS [29].

Many systematic reviews and meta-analyses confirmed the efficacy of combined therapy in controlling asthma symptoms and reducing severe exacerbations and hospitalization. A Cochrane meta-analysis assessed the use of combined inhalers in the treatment of mild asthma when taken on an as-needed, symptom-driven basis for adults or children. Of the six studies included, three [24,27,30] compared as-required combined ICS and fast acting beta-agonist inhaler (FABA) with as required SABA. The results showed fewer exacerbations requiring systemic steroids with combined inhalers (OR 0.45, 95% CI 0.34 to 0.60) and fewer exacerbations requiring hospital admission (OR 0.35, 95% CI 0.20 to 0.60). Compared with SABA alone, any changes in asthma control, preferably measured by the Asthma Control Questionnaire (ACT), though in favor of FABA/ICS, were small and not statistically significant. When compared with regular maintenance use of ICS, as-required fixed-dose combination inhalers did not lead to a significant difference in the annual rate of severe asthma exacerbations requiring systemic steroids (rate ratio 0.90, 95% CI 0.76 to 1.06), but did reduce the odds of asthma-related hospital admission, emergency department visit, or urgent care visit (OR 0.63, 95% CI 0.44 to 0.91) [16]. Rodriguez-Martinez et al. reviewed the literature on the use of ICS on an intermittent or as-needed basis as an add-on therapy to SABA or formoterol, with or without ICS use during stable periods of the disease (ICS alone or ICS-formoterol). Seventeen studies were included (16 RCTs and 1 meta-analysis). Data showed that the use of ICS on an intermittent or as-needed basis (as an add-on therapy to SABA) was more effective than treatment with SABA alone [31].

Some protocols have been presented to evaluate new strategies for controlling asthma exacerbations, considering the well-known effects of prolonged SABA use as rescue therapy. MANDALA is a global Phase 3, randomized, double-blind, parallel-group, event-driven asthma exacerbation study. The study will compare the effect of two fixed dose combinations of salbutamol/budesonide (180/160 μg and 180/80 μg) versus salbutamol for as-needed use for treating symptoms in patients with various ICS-containing maintenance therapies. The study will recruit 1000 patients per treatment group for adults/adolescents and 50 patients per treatment group for patients aged 4–11 years. This study, if successful, will confirm salbutamol/budesonide pMDI as a rescue therapy irrespective of background asthma therapy [32]. The CARE study will be a 52-week phase III RCT on children aged 5 to 15 years with mild asthma treated either with budesonide-formoterol or salbutamol. This will be the first RCT to assess the safety and efficacy of as-needed budesonide-formoterol in children with mild asthma [33].

In conclusion, increasing evidence supports taking ICS whenever SABA is taken. However, in large population studies, the severity of asthma is usually defined by the prescribed treatment, and it might be possible that patients considered to have mild asthma (for example, those treated with SABA only) had undertreated asthma and therefore experienced fewer exacerbations when treated with formoterol/ICS rather than with SABA alone.

#### 3.1.2. Recommendation

*For young children aged 6–12 years with infrequent symptoms (step 1), as needed, low-dose ICS associated with SABA for symptom relief is suggested.*

***Quality of evidence: low. Strength of recommendation: B***

*For adolescents aged >12 years with infrequent symptoms (step 1), low-dose ICS associated with SABA or low-dose ICS-formoterol are the preferred strategies.*

***Quality of evidence: moderate. Strength of recommendation: B***

### 3.2. PICO Question 2. In Children with Asthma, Is Daily Therapy with ICS More Effective than Daily LTRA?

#### 3.2.1. Executive Summary

For children aged >12 years, all guidelines suggest the use of ICS (combined or not with formoterol) as the preferred treatment for children with symptoms at presentation that clearly indicate the need for maintenance therapy (GINA and NAEPP step 2 [2,6]). Daily LTRAs such as montelukast or zafirlukast are considered in NAEPP guidelines as an alternative controller option for patients who are unable or unwilling to use ICS or who cannot tolerate ICS. If asthma is uncontrolled with a low- or medium-dose of ICS (steps 3–5), LTRA may be considered in addition to ICS, and the response to treatment will be reviewed in 4–8 weeks [2,5,6].

However, LTRAs have an increased risk of adverse consequences in terms of neuropsychiatric events and a need for monitoring that make their use less desirable. In 2020, the US Food and Drug Administration issued a boxed warning for montelukast because of adverse effects related to serious behavior- and mood-related changes.

The evidence suggests that ICS have anti-inflammatory effects and reduce airway hyperresponsiveness, allowing them to control asthma symptoms and reduce the risk of future exacerbations and the related decline in lung function [34]. Most of the benefits associated with ICS are obtained with low daily doses (budesonide 200 μg daily or fluticasone 100 μg daily). However, adherence to ICS is poor in adults and children, with average dispensing covering less than 25% of days, with consequent over-reliance on SABA, and with an increased risk of severe exacerbations and death [35]. LTRA blocks leukotrienes that are involved in airway smooth muscle contraction, vascular permeability, mucus production, and airway inflammation [34]. Although montelukast improves exacerbation rates compared with placebo in school-aged children, it is not as effective as daily ICS [36]. In a meta-analysis comparing montelukast vs. ICS, ICS were generally more effective than montelukast in mild-to-moderately persistent asthmatic children under controlled clinical conditions [37]. A systematic review of RCTs evaluating the treatment of mild to moderate persistent asthma with montelukast or ICS in children aged 2 to 18 years showed improvement in asthma symptoms with both treatments but more with ICS and concluded that ICS should be the first-line treatment in this population [38]. Over the last 5 years, only three studies compared daily therapy with ICS (budesonide) versus daily LTRA (montelukast) in children with mild persistent asthma. The study by Mane et al. included 54 children aged 3 to 12 years with mild persistent asthma who were randomized to receive either oral montelukast or inhaled budesonide. The results showed that children on inhaled budesonide were more controlled in terms of asthma symptoms, required fewer reliever medications, had fewer episodes of night awakening due to asthma, and had an improvement in their activity [39]. The other two studies, both retrospective and observational, evaluated the effectiveness of montelukast versus budesonide inhalation suspension (BIS) in children with mild persistent asthma. The study conducted by Jina Shin et al. in Korea showed that patients on montelukast had better adherence and a longer time to lose persistency in comparison with BIS patients. On the other hand, asthma-related total costs were higher for montelukast, likely due to higher pharmaceutical costs and better treatment adherence/persistence [40]. The Chinese study suggested that both BIS and montelukast were effective in children with mild persistent asthma, with potentially greater benefits for montelukast regarding asthma control and health care costs [41]. Both studies were set in a real-world setting where treatment adherence and persistence were known to be suboptimal.

In addition, recent evidence suggests that genetic variation may contribute to variability in montelukast response since a small percentage of individuals may benefit while most will not obtain any effects [42].

That said, the Th_2_-high phenotype characterized by elevated fractional exhaled nitric oxide (FeNO), peripheral eosinophilia, elevated allergen-specific IgE, or positive skin tests may respond better to daily ICS rather than LTRA [43,44].

#### 3.2.2. Recommendation

*ICS should be the first-line treatment for children and adolescents with mild to moderate persistent asthma, particularly those with features suggesting Th_2_ inflammation.*

***Quality of evidence: high. Strength of recommendation: A***

*ICS is more effective than an LTRA.*

***Quality of evidence: moderate. Strength of recommendation: A***

*Due to its simplicity of administration, a trial with LTRA may be considered an alternative to ICS in patients with poor adherence to treatment or who have difficulty using inhalers. LTRA is also considered an add-on therapy when daily ICS cannot provide adequate symptom control.*

***Quality of evidence: low. Strength of recommendation: C***

### 3.3. PICO Question 3. In Children with Uncontrolled Asthma Symptoms Despite Low Daily ICS, Is Increasing the Dose of ICS More Effective than Adding LABA or LTRA?

#### 3.3.1. Executive Summary

Children and adolescents with persistent asthma despite step 1 or step 2 treatments should be reviewed to assess comorbidities and modifiable risk factors (such as inhalation technique, exposure to allergens, and adherence to daily therapy) and eventually increase therapy. Step-up therapy includes: increasing to medium-dose ICS, starting the combination low-dose ICS/LABA as daily controller therapy, or adding LTRA to daily ICS. In adolescents > 12 years of age, both the NAEPP Expert Panel and the GINA report recommend with high certainty of evidence for steps 3 and 4 (moderate to severe asthma) the use of the single-inhaler maintenance and reliever therapy (SMART) approach (low- or medium-dose ICS-formoterol in a single inhaler used as both daily controller and reliever therapy) [2,6]. A moderate certainty of evidence has been reported for ages 4- to 11-year-olds since in this age group only one study has demonstrated benefit and a lower risk of growth suppression among those using SMART compared with higher daily doses of daily ICS [43].

In the case of children 5 to 11 years of age, when asthma is not adequately controlled with low-dose maintenance ICS with as-needed SABA, treatment includes: (1) increasing ICS to a medium-dose; (2) changing to low-dose ICS-LABA; (3) adding LTRA; or (4) switching to SMART therapy [2,6].

The SMART approach must be reserved for those patients able to correctly perceive their symptoms since the risk of under or overtreatment is consistent.

Several previous studies, mostly performed in adolescents, demonstrated that the SMART approach is more effective in reducing exacerbations compared to a higher daily dose of ICS with an as needed SABA, a daily dose of ICS plus LABA and an as-needed SABA, and a higher daily dose of ICS plus LABA and an as-needed SABA [45,46,47,48,49]. In adults and adolescents with mild asthma, a double-blind randomized phase 3 trial comparing twice-daily placebo plus budesonide 200 μg/formoterol 6 μg used as needed versus maintenance therapy with twice-daily budesonide (200 μg) plus terbutaline (0.5 mg) used as needed showed that the combination budesonide/formoterol used as needed was non-inferior to budesonide maintenance therapy in terms of asthma exacerbations. As a secondary endpoint, the study demonstrated that the median daily dose of ICS was lower in the budesonide/formoterol group than in the budesonide maintenance group (66 μg vs. 267 μg) [23].

In a cohort of adolescents with persistent asthma, patients were randomized for 12 weeks in five arms, including: twice-daily fluticasone 50 μg, twice-daily fluticasone 100 μg, twice-daily fluticasone 50 μg/salmeterol 12.5 μg, twice-daily fluticasone 100 μg/salmeterol 12.5 μg and twice-daily placebo. Results showed a significant improvement in forced expiratory volume in 1 s (FEV_1_) at 12 weeks in all groups compared with placebo (*p* < 0.05); however, the combined therapy with ICS/LABA (50 μg/12.5 μg and 100 μg/12.5 μg twice-daily) was superior to ICS alone (50 μg and 100 μg twice-daily). In particular, the proportion of patients achieving at least 15% improvement in FEV_1_ was higher in the ICS/LABA arms than in the ICS alone arms. No difference among groups was found in asthma symptoms score, average use of as needed albuterol/salbutamol, or adverse events [50].

A post hoc analysis of six RCTs demonstrated that in adolescents with persistent asthma (12–17 years), the efficacy of maintenance and reliever therapy with budesonide/formoterol (SMART therapy) in terms of time to severe exacerbations, number of severe exacerbations, symptoms score, night-time awakenings, as-needed inhalations, morning peak expiratory flow (PEF), and FEV_1_ was similar or more effective than daily budesonide plus as needed terbutaline, daily budesonide/formoterol plus as needed terbutaline, daily budesonide/formoterol plus formoterol, and daily salmeterol/fluticasone plus as needed terbutaline. In addition, the budesonide/formoterol maintenance and reliever therapy arm received the lowest daily ICS dose [51].

The PALLADIUM study on asthmatic patients older than 12 years compared five different arms, including: high-dose mometasone/indacaterol (320 μg/150 μg) once daily, medium-dose mometasone/indacaterol (160 μg/150 μg) once daily, high-dose mometasone (400 μg) twice-daily, medium-dose mometasone (400 μg) once daily; and high-dose fluticasone/salmeterol (500 μg/50 μg) twice daily. High-dose mometasone/indacaterol and medium-dose mometasone/indacaterol groups presented greater improvement in FEV_1_ after 26 weeks of treatment compared to the same dose of ICS used alone; high-dose mometasone/indacaterol administered once daily was non-inferior to high-dose fluticasone/salmeterol administered twice daily. Furthermore, patients treated with ICS/LABA showed higher improvement in the asthma control questionnaire (ACQ)-7 score at week 26 and greater values of PEF compared to patients treated with ICS alone [52].

In younger children with persistent asthma (<12 years), evidence shows that the administration of fluticasone 100 μg/formoterol 10 μg twice daily is superior to fluticasone 100 μg twice daily alone in improving FEV_1_ after 12 weeks of treatment [53]. Moreover, the combination of mometasone 100 μg/formeterol 10 μg twice daily was shown to be more effective in improving FEV_1_ across 12 weeks of treatment compared to momentasone 100 μg twice daily alone. Children in the ICS/LABA group reported less salbutamol use and fewer adverse events [54].

A recent meta-analysis, including 11 studies conducted in children, assessed the efficacy of fluticasone/salmeterol compared to fluticasone alone. Compared to fluticasone alone, patients treated with the combination of fluticasone/salmeterol obtained higher FEV_1_ and experienced fewer exacerbations, with no difference in adverse events. However, when the ICS dose was doubled, the two treatments were equivalent [55].

In children, there is little evidence for adding a leukotriene receptor antagonist to low-dose ICS. An RCT conducted in 2019 in 135 children demonstrated that the combination of montelukast sodium tablets with inhaled budesonide reduced asthma symptoms and hospital stays compared to montelukast or budesonide alone. This combination resulted in a higher increase in FEV_1_, forced vital capacity (FVC), and PEF and reduced levels of inflammation mediators such as tumor necrosis factor-α (TNF-α), interleukin-4 (IL-4), IL-8, CD8 + cell count, immunoglobulin E (IgE), and hypersensitive C-reactive protein [56]. However, a recent systematic review reported that salmeterol/fluticasone was superior to montelukast or montelukast + fluticasone in children and adolescents aged 4 to 18 years with bronchial asthma. Four weeks of treatment with salmeterol/fluticasone were associated with a lower risk of exacerbation, a significant improvement in PEF, and a higher level of asthma control [57].

In addition, the recent FDA warning for the use of LTRA in adolescents must always be taken into account [58].

#### 3.3.2. Recommendation

*In adolescents > 12 years with moderate persistent asthma (step 3 or 4), despite the assessment of comorbidities and modifiable factors, a SMART approach with ICS/formoterol as maintenance and reliever therapy is the preferred option. ICS/LABA as maintenance and SABA as a reliever are valid alternatives; however, adherence to therapy must be checked since there is a higher risk of SABA overuse.*

***Quality of evidence: high. Strength of recommendation: A***

*In children 6 to 11 years with moderate persistent asthma (step 3 or 4), despite the assessment of comorbidities and modifiable factors, a SMART approach with ICS/formoterol as maintenance and reliever therapy (with a check of inhalation technique) and ICS/LABA as maintenance and SABA as reliever are both valid alternatives; increasing the ICS dose can be considered.*

***Quality of evidence: moderate. Strength of recommendation: B***

### 3.4. PICO Question 4. In Children with Uncontrolled Asthma Symptoms Despite Daily Therapy, What Is the Preferred Option between Increasing the Therapy or Assessing Modifiable Factors (Adherence, Inhalation Technique, Exposure to Allergens)?

#### 3.4.1. Executive Summary

Asthma is the most common chronic disease in children [59]. ICS are the mainstay of treatment for most asthma patients [60], as these medications are able to reduce inflammation. Despite regular use of therapy [61], some children (5% of the child population with asthma) experience continuous and frequent asthma symptoms and exacerbations defined as problematic severe asthma. Among children with problematic severe asthma, it is crucial to distinguish those with “difficult-to-treat asthma” because of a wrong diagnosis or modifiable underlying factors (asthma plus co-morbidities) from those with true “severe, therapy-resistant asthma” (STRA) who have persistent symptoms despite optimization of the basics of asthma management [62].

All children who meet the criteria for problematic severe asthma despite receiving treatment should have a comprehensive multidisciplinary assessment. After confirmation of the asthma diagnosis (confirmed wheeze, reversible airflow obstruction with spirometry, detailed medical history and examination, skin prick tests for aero and food allergen sensitization, measurement of airway inflammation with exhaled nitric oxide, exclusion of other diagnosis), modifiable factors that could influence patients’ response to therapy and comorbidities must be assessed. Modifiable factors include adherence to therapy, inhalation technique, and environmental factors [62].

Hence, when addressing the issue of uncontrolled asthma, taking care of these modifiable factors as well as increasing therapy can be good strategies to attain better control of the disease. While many studies have evaluated the benefits of “stepping-up” the therapy when asthma is not well controlled, less is known about what can be done about modifiable factors and whether these interventions can actually be beneficial for asthmatic children.

Adherence to treatment is one of the most important issues for children with difficult asthma. Aschalew and colleagues found that both adherence to therapy and inhalation techniques were relevant in determining the risk of uncontrolled asthma [63], and Basharat and colleagues highlighted the correlation between high adherence to therapy and good asthma control [64]. In the study by Giubergia, improving inhalation technique as well as environmental conditions resulted in a significant increase in lung function after 6 months. More specifically, this study divided the population into “severe difficult-to-treat asthma” and “severe treatment-resistant asthma”; both groups underwent the same protocol with monthly follow-up visits. Comorbidities, environmental control, therapy adherence, pulmonary function, and asthma control were assessed at each visit, as was the need for any therapy modifications. Interestingly, after 6 months of follow-up, patients from both groups showed significant increases in lung function; however, the STRA group still needed a significantly higher dose of LABA and ICS [65]. There is increasing evidence that mobile apps with daily reminders may be effective in ameliorating adherence to treatment. Mobile apps can be linked with electronic monitoring devices (EMD) attached to a patient’s inhaler. This combination can detect the effective use of the inhaler and the administered doses. Information received by EMD is shared with healthcare professionals and discussed with the patient. Inhalation technique should be checked at every visit, as up to 40% of children with severe asthma may have suboptimal inhaler technique [61].

When assessing poor control and adherence to therapy in patients with STRA, the psychological component must be considered since a high percentage of patients with asthma also have psychological issues [62]. Studies have demonstrated that adequate psychological assessment and patient education regarding the mechanism and nature of asthma should be considered important interventions to improve patient compliance [66].

Comorbidities such as allergic rhinitis, dermatitis, or food allergies [67,68] and obesity must be assessed when treatment seems ineffective. Allergic rhinitis has been associated with severe asthma attacks [68] and a high body mass index (BMI) with a higher risk of treatment failure likely due to dysanaptic growth and impaired ICS response [69,70].

Environmental factors are difficult to assess and modify. Inhaled pollutant particles prolong the oxidative stress of respiratory cells and favor a chronic inflammatory state of the mucous membranes, which, over time, can worsen asthma symptoms, as demonstrated by a study on the pediatric population in rural Africa [71], which showed that humidity, visible mold growth, the use of paraffin for cooking, and second-hand smoke were associated with a two- to three-fold increased risk of symptoms. The wholesomeness of the air, therefore, remains a key point in the prevention of asthmatic symptoms.

In this context, the use of telemedicine can contribute to achieving good asthma control and distinguishing some of the modifiable factors associated with persistent symptoms. Digital devices such as smartphones, tablets, or computers interfaced with portable medical devices such as monitors for air quality and pollen counts can remotely describe the environment where the patient lives and suggest behaviors to avoid exposure to triggers. Smartphone applications, text messaging, and alerts can promote adherence to daily therapy and self-management of symptoms. Educational videos and tutorials on the administration of therapy and the correct use of inhalers can increase symptom control and adherence to treatment [72,73].

#### 3.4.2. Recommendation

*In patients with uncontrolled asthma despite treatment, before stepping up therapy, increasing the ICS dose, or adding a new medication, modifiable factors, and comorbidities must be reassessed. If modifiable factors are identified and addressed but asthma control remains poor, increasing therapy may be necessary.*

***Quality of evidence: low. Strength of recommendation: C***

### 3.5. PICO Question 5. In Children with Asthma, Is Metered Dose Inhaler (MDI) Preferred to Dry Powder Inhalers (DPI)?

#### 3.5.1. Executive Summary

The patient must be able to use the inhaler appropriately to ensure adequate lung delivery and maximize the benefit of the medication. A good inhalation depends on the distribution of the active substance in the airways and, therefore, the effectiveness of the therapy. However, the correct use of most devices is not intuitive and requires proper education. In addition, having more than one device for relief and maintenance therapy can be confusing since the inhalation technique can be different.

Metered-dose inhalers (MDI) and dry powder inhalers (DPI) are the most common asthma devices available on the market. MDI inhalers are pressurized devices that involve the delivery of the pre-dosed drug in a high-velocity nebulized solution. MDI is small and portable but requires coordination between actuation and inhalation. For this reason, spacers or holding chamber devices are recommended to help with coordination, slow down the speed of nebulization, reduce oropharyngeal deposition, and ensure an adequate distribution into the lungs. However, the spacer is not small or very portable, and when used with a mask, as for small kids, the inhalation of the medication can be compromised by the movements of the child. Another disadvantage is that MDI does not have a dose counter, so it is difficult to tell how much drug remains in the device.

On the other hand, DPIs do not need spacers, incorporate dose counters, and are easier to teach than MDIs. DPIs need to be triggered by the patient’s breathing, so these devices must be reserved for schoolchildren aged 10 years and older. The drug is delivered in the form of a dry powder, which can reach the lower airways once the acceleration of the inhalation flow has disaggregated the powder into smaller particles [74]. Since the young child is unable to generate an adequate inspiratory rate (at least 15 L/min), the GINA report excludes the use of DPI in children under 5 years of age [2].

Dolovich et al. reviewed the literature published on aerosol devices (MDI, DPI, and nebulizers) and concluded that no difference could be found in any of the outcomes analyzed and that all devices could be used in a variety of clinical settings [75]. However, a significant proportion of children treated with asthma medication do not correctly inhale the medication, regardless of the device [76,77].

Recent evidence on the device being preferred by children is scarce. In a retrospective study on 58 patients between the ages of 6 and 18 years, those (n = 24) offered to change from MDI to DPI showed a significant reduction in lung function (FEV_1_ reduced from 98.5% to 91%, *p* = 0.013; FEF_25–75%_ reduced from 89.5% to 76%, *p* = 0.041) [78]. Other available studies focused on the appropriateness of the inhalation technique, reaffirming that the efficacy of the treatment is strongly influenced by the correct execution of the various steps required for the delivery of the drug in each device. A prospective study analyzed by means of a questionnaire the correctness of the inhalation technique in 100 patients aged between 6 and 18 years one month after a detailed explanation of the inhalation technique followed by a practical demonstration. This study showed that patients learned better how to use DPI compared to MDI, with a lower error rate in the execution of the therapy (correct steps: 60.6% MDI; 80% Turbohaler; 58% capsule-based DPI) [76]. A recent cross-sectional study demonstrated that the percentage of appropriateness in the use of DPI in a pediatric population aged between 7 and 17 years was superior to MDI, (whereas for DPI it was 38.5% for Turbohaler, 28.9% for Diskus, and 12.5% for Handihaler, respectively, vs. 13.4%) [79].

#### 3.5.2. Recommendation

*Currently, there is no clear opinion on the superiority of one inhaler over the other. The decision is based on the patient’s needs, abilities, and preferences. Accurate description of the devices and demonstration of inhalation techniques are mandatory in asthma clinics to provide the patient and the family with the correct information to take the medication. DPI may be reserved for older children aged at least 10 years.*

***Quality of evidence: low. Strength of recommendation: D***

### 3.6. PICO Question 6. Which Patients with Asthma Can Benefit from Immunotherapy?

#### 3.6.1. Executive Summary

Allergen-specific immunotherapy (AIT) is the only available treatment that targets one of the causes of asthma. It consists of the administration of aeroallergen extracts, generally at increasing doses in the initial phase (“build-up phase”) and then at a maintenance dose. Immunotherapy can be administered sublingually or subcutaneously, and the dose is delivered throughout the year or before or during the allergen season [80]. The aim of AIT is to achieve immune tolerance to the allergen by acting on innate and adaptive immunity, ensuring long-term efficacy. In particular, AIT transiently increases the concentration of allergen-specific IgE in the blood and then decreases it by reducing the Th2 immune response and the number and activity of mast cells, basophils, and eosinophils. Moreover, AIT promotes the production of Treg cells and, therefore, of IL-10 and other factors responsible for immunosuppression [80].

In relation to the immunological role of AIT, the need to identify biological markers to predict and monitor the effects of immunotherapy in patients is developing. No markers have yet been validated; however, there are ongoing studies on biomarkers related to innate and adaptive immunity. For example, after AIT, there is an increase in allergen-specific antibody isotypes, such as IgG1, IgG2, IgG4, and IgA [81].

Currently, asthma treatment guidelines and recommendations have conflicting opinions on AIT. The NICE 2021 guideline does not even mention AIT as a therapeutic option for pediatric patients with asthma [82]. The BTS-SIGN 2019 guideline, although recognizing the benefits of respiratory symptoms control, states that evidence is unclear and therefore AIT is not recommended in children and adults with asthma [5]. On the contrary, GINA 2022 considers the use of SLIT in adolescents (more than 12 years old) and adults with mild to moderate asthma and allergic rhinoconjunctivitis (AR) driven by house dust mites in addition to standard therapy [2]. The NAEPP 2020 guideline recommends with moderate certainty of evidence the use of SCIT in individuals 5 years of age and older with mild to moderate asthma as an additional treatment to standard pharmacotherapy, in those patients whose asthma is controlled. On the other hand, NAEPP recommends with a moderate certainty of evidence against the use of SLIT in individuals with persistent allergic asthma, being that SLIT did not benefit the critical outcomes of exacerbations, asthma control, and quality of life [6].

The European Academy of Allergy and Clinical Immunology (EAACI) guideline 2019 states that AIT is recommended in monoallergic patients whose AR is driven by a specific allergen, while in patients polysensitized by analogous allergens, it is possible to use AIT with the main allergen or with a mixture of allergens; AIT with the allergen that gives more allergic effects or consecutive AIT with different allergens represent the main therapeutic strategies in polysensitized patients by non-homologous allergens. In particular, SLIT (tablets or drops) administered pre-, pre-/coseasonal, or in continuous form are effective in the short term for grass pollen AR, while continuous SLIT tablets are effective in the short term for house dust mite (HDM) AR; a continuous SLIT for 3 years is effective in the long term, therefore for more than 2 years from the end of the immunotherapy, for grass pollen (tablets and drops) and HDM (just tablets) AR [83,84]. In the EAACI guidelines dealing with dust mite allergic asthma, SCIT and SLIT (just drops) are recommended in addition to basic medical therapy in controlled or partially controlled asthma as they have demonstrated a reduction in symptoms and medication use with an improvement in the quality of life; in the case of uncontrolled dust mite allergic asthma, it is recommended first to achieve asthma control and then add AIT [85,86]. The German guidelines of 2022 (DGAKI) recommend AIT in the pediatric population with AR due to grass allergy because safety and efficacy have been demonstrated, as well as a reduction in symptoms and use of drug therapy in the short and long term and a preventive effect on the onset of asthma. Regarding children with allergic asthma from grass pollen, SCIT is recommended from the age of 3 as it reduces the symptoms and the need for medical therapy, and in some cases also bronchial hyperreactivity, thus acting on the exacerbations. The evidence on SLIT is more limited; however, efficacy and safety have been demonstrated in the short term. For patients with tree pollen allergy, AIT is recommended in children with AR with a demonstration of efficacy and safety up to 6 years after the suspension of AIT. For the pediatric population with asthma due to birch allergy, there are not enough studies; therefore, AIT is indicated but with low evidence. Furthermore, alder/hazel/oak pollen have a high cross-reactivity with birch, and therefore the German guidelines state that it is possible to use the same AIT. As far as allergy to dust mites is concerned, the DGAKI recommends AIT in particular in asthmatic subjects for its demonstrated efficacy in terms of reduction of symptoms, need for therapy, reduction of exacerbations, and safety. The evidence for AIT in children and adolescents with HDM rhinoconjunctivitis is smaller but still recommended. Finally, the German guidelines also give indications for ragweed allergy, in which SLIT is recommended in the pediatric population both in rhinoconjunctivitis and in allergic asthma; studies evaluating SCIT are few [87].

AR is a risk factor for the development of asthma, especially when characterized by early onset in childhood, allergenic polysensitization, and the presence of moderate-to-severe symptoms. Trials suggest that beginning AIT has a preventive effect on asthma in patients with AR, in particular if started when symptoms are mild and when the child is younger. Conflicting results are reported about its preventive role in new sensitizations [81]. A systematic review with meta-analysis published in 2022 by Ferraia et al., confirms the preventive action of AIT (both SCIT and SLIT) for the development of asthma in the pediatric population with allergic conditions such as rhinitis, AR, and/or atopic dermatitis, monosensitized against whichever aeroallergen (grass pollen, birch, or HDM), performing IT for at least 3 years. The preventive action was also evaluated for a 3-year post-treatment period (follow-up) [88].

A few studies have analyzed the use of AIT in children with asthma in the last 5 years [81,89,90,91,92,93,94,95,96,97,98,99].

Zielen et al. studied grass-pollen SLIT in both adults and children with moderate-severe AR and demonstrated that treated patients required fewer medications for AR compared to controls [RC −0.188 [95% CI −0.222 to −0.155]; *p* < 0.001; Age < 18y: CR −0.127 [CI 95% −0.145 to −0.11]; *p* < 0.001]. However, no difference was found in asthma onset. Interestingly, in the group treated with SLIT, asthma advanced more slowly in terms of asthma medication prescription, as inferred from the proxy prescription data set [89].

A French retrospective study conducted from 2012 to 2016 in a population aged more than 5 years with moderate-severe AR found that patients who had received at least two immunotherapy prescriptions over two consecutive years had a lower need for AR medications. Moreover, patients with AR and asthma who received SLIT-grass pollen tablets had less need for asthma medication than patients who did not receive immunotherapy [93], and there was a minor evolution of AR into allergic asthma, suggesting that SLIT may slow down the so-called “atopic march.”

A case-control study published in 2019 including 60 (30 treated with SCIT (subcutaneous immunotherapy) + asthma standard treatment and 30 treated only with standard treatment) children aged between 5 and 10 years with mild-moderate asthma and sensitization to house dust mites showed that SCIT in addition to standard asthma medications resulted in a reduction in the need for baseline therapy (according to GINA 2015 recommendations) in the SCIT group at 3 and 6 months. Both groups reported reductions in asthma symptoms at 3 and 6 months [90].

In a large German study including 39.167 individuals aged >12 years, in the 10.5% of the cohort who received AIT, AIT was associated with a significant reduction in the risk of asthma progression, particularly in adolescents (12–17 years) [91].

In a recent case-control study published in 2022, immunotherapy in addition to standard therapy for moderate-severe asthma (treated with the combination ICS/LABA) was associated with long-lasting immunotolerance towards the allergen. The study included 30 children aged between 5 and 12 years with moderate-severe asthma treated with ICS/LABA or ICS/LABA + HDM SCIT. The study showed that Th17 lymphocytes and IL-17 levels were elevated in asthmatic patients compared to healthy subjects. A significant reduction of Th17 lymphocytes and IL-17 levels was demonstrated in both groups; in addition, the group treated with ICS/LABA + HDM-SCIT also showed an increase in Treg cells and IL-10 levels, suggesting that AIT would promote, when associated with corticosteroids, immunosuppression and therefore long-term immunological tolerance towards allergens [92].

The retrospective study published in 2018 by Amat et al. demonstrated that in children and adolescents with moderate-severe asthma with documented allergy to house dust mites aged 6 to 18 years, AIT was associated with reduced asthma exacerbations (after 1 year from 2.1 ± 4.5 to 0.38 ± 0.68; *p* < 0.001; after three years to 0.44 ± 0.58; *p* = 0.01) and reduced doses of inhaled corticosteroids (from 500 μg/day to 300 μg/day after 1 year—*p* < 0.01 and to 200 μg/day after 3 years—*p* = 0.01) after 1 and 3 years from the initiation of immunotherapy [95].

A recent review, including 13 trials on children with AR and mild-moderate asthma receiving sublingually or subcutaneously dust mite AIT, demonstrated that immunotherapy generally reduced asthma symptoms compared to standard therapy alone. The review shows that subcutaneous IT is more effective than SLIT in reducing the symptoms of allergic rhinitis and asthma and medication use; notably, data concerning the efficacy of SLIT are conflicting since some studies prove the effects on asthma symptoms and control while others don’t [94]. However, one of the studies in the review demonstrates the good efficacy of the immunotherapy administered in the dual alternative (which consists of a first subcutaneous administration with a faster action, followed by a sublingual administration with an easy intake, fewer side effects, and greater compliance). An increase in FEV_1_ also occurred in the group of patients given the SCIT/SLIT combo [97]. Other subsequent reviews have then demonstrated the same clinical efficacy (reduction of exacerbations, better quality of life, reduction of asthmatic symptoms and pharmacotherapy, improvement of lung function) and safety of sublingual immunotherapy compared to SCIT [98,99].

A systematic review of SCIT and SLIT in children with mild to moderate allergic asthma and AR (allergy to grasses, dust mites, mold, or polysensitized) showed that AIT was associated with improvement in asthma symptoms, quality of life, lung function, and a reduction in medicine use [100].

Randomized observational studies are needed to include all pediatric age groups (preschool, school, and adolescent), a study in which aeroallergens can be addressed, consider the different methods of administration and a specific dose, and consider the possible risks and/or benefits. Further studies on laboratory investigations (e.g., a spirometric evaluation, the determination of FeNO, or other measurable outcomes) are necessary for identifying biomarkers of AIT efficacy.

#### 3.6.2. Recommendation

*AIT is effective in the pediatric population with IgE-mediated allergic respiratory diseases. Candidates for AIT are patients with mild to moderately controlled asthma who need long-lasting or multiple drugs to maintain asthma control. Both SLIT and SCIT AIT are beneficial in improving asthmatic symptoms and quality of life and reducing the use of short- and long-term medications.*

***Quality of evidence: moderate. Strength of recommendation: B***

### 3.7. PICO Question 7. In Children with Uncontrolled Asthma Symptoms Despite a Daily Medium-Dose of ICS Combined with LABA, Is Increasing the Dose of ICS More Effective than Adding the LAMA Tiotropium?

#### 3.7.1. Executive Summary

According to the main international guidelines [2,5,6,101], a stepwise pharmacologic approach should be used in moderate-to-severe asthmatic patients to reach symptom control, relieve symptoms when they occur, and minimize the risk of exacerbations. For patients with uncontrolled asthma symptoms despite medium- or high-dose ICS/LABA or ICS/formoterol (steps 4–5), either increasing the ICS doses or adding LAMA such as tiotropium in a separate inhaler are proposed strategies [102]. Furthermore, the ERS/ATS Guidelines on severe asthma agree on the positive effects of adding LAMA both for children and adolescents (strong recommendation, moderate quality of evidence) on the basis of two trials performed in adolescents (14–17 years of age) and children (6–11 years) with severe uncontrolled asthma [101].

There is no clear consensus on whether increasing the dose of ICS is more effective than adding LAMA. A systematic review and meta-analysis of 8 RCTs studied the role of increasing ICS during exacerbations and concluded that, despite the higher risk of non-serious adverse events, the risk of exacerbation was significantly reduced (OR 1.05, 95% CI 0.73–1.51) (moderate quality of evidence) [103]. Similar results were found for consistent dosage increases [104,105]. However, a separate analysis of several RCTs showed no significant clinical improvement but an increased rate of adverse events such as diminished linear growth [106,107].

Vogelberg et al. assessed the efficacy of tiotropium in children. Patients treated with 5 mg (n = 135) and 2.5 mg (n = 135) of tiotropium significantly improved peak spirometric parameters (FEV_1_ at weeks 24 and 48 and peak FVC at week 24) compared with placebo in children with moderately symptomatic asthma. Symptom control improved with tiotropium, and the incidence of adverse events was lower in patients receiving tiotropium than in those receiving a placebo (most adverse events were mild or moderate in intensity) [108]. Further studies confirmed the role of tiotropium as an add-on therapy in children with moderate-to-severe asthma. In particular, 5 µg of tiotropium improved peak FEV_1_ within 3 h after dosing in a cohort of 401 participants (6 to 11 years) in a double-blind, placebo-controlled trial [109]; similar results were demonstrated for the same tiotropium doses (5 µg) and outcomes (peak FEV_1_) in an older population (398 participants, 12 to 17 years) with an analogous study design [110]. A systematic review and meta-analysis of 7 RCTs showed positive effects of tiotropium add-on therapy, such as a significant increase in FEV_1_ in patients receiving tiotropium compared to those receiving placebo (mean difference, 110 mL; [95% CI 80–140 mL]; *p* < 0.001). In particular, the benefit of tiotropium was greater after longer therapy (24 weeks) versus relatively short therapy (12 weeks or 4 weeks) [111]. Some studies conducted in mixed cohorts (pediatric and adult populations) have questioned whether the use of tiotropium was affected by the asthmatic Th2 phenotype and have concluded that its positive effects on asthma control and reduction of exacerbations were independent of IgE levels or eosinophilic count [112,113]. The confirmation of these results in the pediatric population has been demonstrated by specific studies [114].

Several studies conducted mainly in adolescents demonstrated the safety and effectiveness of the add-on LAMA approach [115,116,117,118]. In particular, a pooled analysis of 5 RCTs supports the favorable risk-benefit profile of once-daily tiotropium as an add-on to maintenance ICS with or without additional controllers in pediatric patients with symptomatic asthma [116]. The safety of tiotropium was extensively proven in a pooled analysis of 12 RCTs (aged 1 to 75 years) [119].

A systematic review and meta-analysis of 14 RCTs compared tiotropium treatment with standard therapy (ICS or ICS/LABA) in patients with moderate to severe asthma and found a significant increase in spirometric parameters (morning PEF, evening PEF, peak FEV, and trough FEV), with no differences with the control group in terms of adverse events; the study included both adult and pediatric populations but did not distinguish by age cohorts [120]. A recent meta-analysis that included 15 randomized clinical trials with 7122 participants 12 years of age or older with uncontrolled, persistent asthma reported that LAMA vs. placebo as an add-on therapy to inhaled corticosteroids was associated with a lower risk of exacerbations requiring systemic corticosteroids [121].

Similar results came from two other systematic reviews. One analyzed 11 RCTs, including children and adults, and concluded that tiotropium as an add-on to ICS therapy was safe and associated with consistent improvements in lung function across different age groups [122]. The review by Murphy et al. considered 7 RCTs, which included preschool children (n = 102), school-aged children (6–11 years; n = 905), and adolescents (aged 12–17 years; n = 895) with moderate to severe asthma treated with ICS with or without LABA. Once-daily tiotropium (5, 2.5, or 1.25 μg) improved lung function parameters, including peak and FEV_1_, vs. placebo [123].

Chipps et al. in a retrospective cohort study, compared the effectiveness of add-on tiotropium versus increased ICS plus LABA. The study population included patients aged ≥12 years with asthma diagnosis initiated on ICS/LABA. The population was divided into two groups: one group receiving tiotropium Respimat 1.25 µg (two puffs once daily) (the Tio group), and the other group having their ICS plus LABA dose increased (the inc-ICS group). Tio group showed a significant decrease in exacerbation risk (hazard ratio 0.65, 95% CI 0.43–0.99; *p* < 0.05) and a reduction of all-cause visit rate within 12 months (47% lower in Tio group, *p* < 0.0001) and of asthma-related emergency visit rate (74% lower in Tio group, *p* < 0.0001). Equally, all-cause and asthma-related hospitalization rates were 48% and 76%, respectively, lower in the tiotropium group [124].

A study investigated the use of tiotropium compared to inhaled mometasone in patients with low eosinophil levels in sputum affected by mild and persistent forms of asthma (low levels of sputum eosinophilia have been correlated with low response to inhaled corticosteroids), finding no significant differences [125].

Systematic reviews compared separately the add-on therapy of LABA to ICS (21 RCTs) with other add-on therapies (LAMA + ICS or LTRA + ICS) and concluded that tiotropium and LABA had similar efficacy, provided greater improvements in lung function than montelukast as an add-on to ICS, and had comparable and favorable safety profiles [105,126].

A systematic review and meta-analysis of 20 RCTs studied the outcomes and adverse events associated with triple therapy (ICS, LABA, and LAMA) versus dual therapy (ICS plus LABA) in a population of 11,894 children and adults with persistent uncontrolled asthma (3 trials included 1870 patients aged 6 to 18 years). The results of this meta-analysis showed a reduction in severe exacerbation risk in patients on triple therapy (22.7% versus 27.4% in patients on Esadual therapy, risk ratio 0.83 [95% CI 0.77–0.90], 9 trials, n = 9932 patients). Triple therapy was also associated with improvement in asthma control scores compared with dual therapy (standardized mean difference [SMD] −0.06 [95% CI, −0.10—−0.02]; mean difference in ACQ-7 scale, −0.04 [95% CI, −0.07–−0.01], 14 trials, 11,230 patients). No significant difference in asthma-related quality of life, mortality, or serious adverse events was demonstrated between the two groups [127].

A meta-analysis of 17 RCTs analyzed the effectiveness and safety of dual (ICS/LABA) and triple therapies (ICS/LABA/LAMA) in a population of 17,161 adolescents and adults with uncontrolled asthma. Medium-dose and high-dose triple therapies reduced steroid-requiring asthma exacerbations (HR 0.84 [95% CI 0.71–0.99] and 0.69 [95% CI 0.58–0.82], respectively). High-dose triple therapy reduced more frequent steroid-requiring asthma exacerbations compared to medium-dose triple therapy (HR 0.83 [95%CI 0.69–0.996]). No difference was found in asthma-related hospitalizations between triple therapy and medium-dose ICS/LABA. Concerning differences in terms of adverse events, high-dose triple therapy results in a reduction of all-cause asthma-related adverse events compared to medium-dose triple therapy [128].

Tiotropium bromide is the only LAMA licensed for long-term treatment of asthma in patients aged ≥6 years who continue to have symptoms despite controller medication administration [129]. As with tiotropium, glycopyrronium is an anticholinergic with higher selectivity for M3 receptors than for M2 receptors and dissociates more slowly from the M3 receptors than from the M2 receptors [130]. The study of glycopyrronium focuses on its effects on methacholine-induced bronchoconstriction in adults with symptomatic asthma. Here, tiotropium provided statistically superior bronchoprotection at both 24 and 72 h compared with glycopyrronium [130].

#### 3.7.2. Recommendation

*In children and adolescents with moderate to severe asthma, both increasing the dose of ICS + LABA and adding tiotropium in a separate inhaler can be effective in terms of asthma control, reduction of exacerbations, and lung function improvement.*

***Quality of evidence: moderate. Strength of recommendation: B***

### 3.8. PICO Question 8. Considering the Biologics for Severe Asthma, Which Are the Differences among Omalizumab, Mepolizumab, and Dupilumab?

#### 3.8.1. Executive Summary

Most children with asthma achieve control with low- to medium-doses of ICS; however, approximately 2% to 10% develop severe asthma with uncontrolled symptoms, lung function impairment, and frequent exacerbations. Biologics should be considered as add-on treatments when asthma meets the criteria described in steps 5 or 6 of NAEPP [6] or step 5 of GINA [2] and is therefore uncontrolled despite LABA + daily medium- to high-dose ICS, with or without other controllers such as LTRA, LAMA, azithromycin, or oral corticosteroids.

In children with severe asthma, the dominant phenotype is the Th2-high phenotype, characterized by eosinophilic inflammation, aeroallergen sensitization with high levels of allergen-specific IgE, elevated peripheral blood eosinophils, and/or high FeNO levels. This phenotype is the primary target of currently approved biologics [131].

At present, the biologics approved for asthma in children are omalizumab, mepolizumab, and dupilumab.

Omalizumab was the first monoclonal antibody approved for severe asthma in patients >6 years old with evidence of allergic sensitization to perennial aeroallergens and IgE. It is a biological medication that inhibits the binding of IgE to the high-affinity FcεRI receptor on the surfaces of mast cells, basophils, plasmacytoid dendritic cells, and eosinophils, preventing allergic responses and airway inflammation. Omalizumab administration consists of a subcutaneous injection with a pre-filled syringe, with dosing and frequency (every 2 or 4 weeks) depending on the baseline free total IgE level. Omalizumab reduces severe asthma exacerbations and ICS doses [132]. Over the last five years, other studies have confirmed the effectiveness of omalizumab in children with moderate to severe asthma.

In an observational single center ‘real-life’ study with a six-year follow-up, Folqué et al. confirmed the clinical improvement with omalizumab, showing a decreased number of admissions for asthma exacerbations and visits to the emergency department. In addition, omalizumab was associated with a reduction in fluticasone dose after 6 months of treatment (from 452 mcd/day to 329.89 mcg/day) and a reduction in the use of LABA after 12 months of treatment (from 98% to 75%). Lung function has significantly improved [133]. Similarly, the multi-center, observational study of Garcia et al. described the long-term outcomes (up to six years) of omalizumab in children with severe persistent allergic asthma and demonstrated that the reduction in moderate-to-severe exacerbations and emergency visits/hospitalizations, the decrease in FE_NO_, and the increase in FEV_1_, remained stable over time [134]. Exacerbations were reduced by 48.5% and severe crisis by 100% after 16 weeks of omalizumab treatment as well as the use of corticosteroids and salbutamol [135]. After 12 months of treatment with omalizumab, adolescents with moderate to severe allergic asthma not only had fewer exacerbations and better ACT scores but also better lung function [136]. When patients discontinued omalizumab while asthma was well controlled, hospitalizations and oral steroid use remained lower than before omalizumab initiation [137].

A post-marketing surveillance evaluated the long-term safety and effectiveness of omalizumab in Japanese pediatric patients with severe allergic asthma and confirmed the good tolerance of the therapy, with 10.2% (13/127) of patients experiencing adverse drug reactions (pyrexia and urticarial) [138]. Considering eosinophils, the STELLAIR study showed that omalizumab effectiveness (assessed through the Global Evaluation of Treatment Effectiveness and annual exacerbation reduction) was similar both in patients with “high” eosinophil counts (≥300 cells/µL) and in patients with “low” eosinophil counts (<300 cells/µL) [139].

Mepolizumab targets interleukin-5 (IL-5), a protein that is involved in the production and activation of eosinophils. By blocking IL-5, mepolizumab can reduce the number of eosinophils in the blood and airways, which can significantly reduce asthma exacerbations and improve lung function in children with severe asthma, particularly those with high levels of eosinophils. It is administered by subcutaneous injection every 4 weeks; children aged 6 to 11 years receive 40 mg, and those older than 12 years receive 100 mg. The medication is available as a pre-filled syringe or pen. Mepolizumab reduces severe asthma exacerbations and the use of oral steroids in patients with severe asthma and blood eosinophil counts greater than or equal to 150 cells/µL [140,141].

Initially approved for children aged >6 years on the basis of a small trial performed in 36 children [142], more recently, the large Mepolizumab Adjunctive Therapy for the Prevention of Asthma Exacerbations in Urban Children-2 (MUPPITS-2) trial by Jackson et al. confirmed its efficacy in this population [143].

The effect of mepolizumab in patients previously treated with omalizumab was assessed by Chapman et al. in a 32-week trial. The switch from omalizumab to mepolizumab resulted in significant improvement in symptom control and health status [77% and 79% of patients achieved the minimum clinically important differences in Asthma Control Questionnaire-5 (≥0.5 points) and in St. George’s Respiratory Questionnaire (≥4 points)]. Additionally, it has been shown that lung function improvement and a substantial reduction in the rate of clinically significant exacerbations (reduced by 64%) and in exacerbations requiring hospitalization or visits to the emergency department (reduced by 69%) have been associated [144].

The safety and pharmacodynamics of mepolizumab in the long term were studied by Gupta et al., who found a positive benefit-risk profile in children with severe asthma and an eosinophilic phenotype. Over 52 weeks, the study recorded a consistent reduction in exacerbation rates (69% lower than baseline, with mean annualized rates reduced to approximately 1 event/year), improvement in asthma control, and a sustained reduction in blood eosinophil counts (from 336 cells/µL to 50 cells/µL) [145]. The same group studied the pharmacokinetics and pharmacodynamics of subcutaneous mepolizumab in children with severe eosinophilic asthma treated every 4 weeks for a total of three doses and demonstrated increased symptom control and a reduction of about 80% in blood eosinophils [142].

Dupilumab is a human antibody directed against the IL-4Ra chain of the receptor for IL-4 and IL-13, the main cytokines of T2 inflammation. IL-4 and IL-13 induce B-cell class switching to IgE production and promote TH2 cell development. It is widely used in children from 6 years of age with moderate-to-severe atopic dermatitis when topical therapies are insufficient or not recommended [146], because, in atopic dermatitis pathogenesis, IL-4 and IL-13 play a key role, decreasing the expression of genes that encode for important elements of the epidermal barrier [147]. Regarding severe asthma, dupilumab has been released for children aged 6 to 11 at a dose of 100 mg every 2 weeks or at a dose of 300 mg every 4 weeks if the weight is <30 kg. For those weighing 30 kg or more, dupilumab is administered at 200 mg every 4 weeks. For adolescents 12 years of age or older, dupilumab can be administered at a dose of 200 mg or 300 mg every 2 weeks. The medication is available as a pre-filled syringe or pen. In the QUEST trial, which also included 107 adolescents, Dupilumab significantly reduced the risk of severe asthma exacerbations, decreased the use of oral steroids, and improved lung function and asthma control in patients with moderate to severe asthma with evidence of high blood eosinophils and elevated FENO levels (>25 ppb) [148,149].

In the 52-week double-blind, placebo-controlled VOYAGE trial, Bacharier et al. found that add-on dupilumab therapy (at a dose of 100 mg for patients ≤ 30 kg and 200 mg for those > 30 kg) reduced asthma exacerbations (relative risk reduction 59.3% [95% CI, 39.5 to 72.6; *p* < 0.001]) and improved lung function and asthma control compared with placebo in children with moderate-to-severe asthma and a blood eosinophil count of >150 cells/µL and/or FENO > 20 ppb. The reduction in exacerbation increased up to 65% in children with blood eosinophil counts greater than or equal to 300 cells/µL [150].

The IDEAL study by Albers et al. [151] showed that patients with severe asthma can be eligible for more than one biologic targeting Th2 inflammation; however, it also highlighted the different phenotypes and endotypes in severe asthma. In fact, it showed that even if there are some overlaps in treatment eligibility, the patient groups eligible for treatment with anti-IgE or anti-IL-5 therapies are often distinct.

The biomarker-driven approach is crucial to personalizing asthma treatment; however, patients may be eligible for more than one biologic, and the phenotype and biomarkers can change over time.

#### 3.8.2. Recommendation

*All biologics for children > 6 years with asthma target Th_2_ inflammation. Biomarkers can be predictive of treatment response to biologics. In severe allergic asthma (high IgE), omalizumab can always be chosen. If the patient shows high FeNO and high blood eosinophils, mepolizumab or dupilumab can be considered (regardless of IgE). All biologics reduce severe exacerbation rates; however, only dupilumab seems to be associated with improvements in lung function. If a patient with moderate to severe asthma is affected by moderate to severe atopic dermatitis or eosinophilic esophagitis, dupilumab might be the best choice. On the contrary, if the patient suffers from chronic spontaneous urticaria, they may likely benefit from omalizumab. Although all are administered subcutaneously, the number and frequency of injections vary substantially between biologics.*

*A trial of 4 to 6 months is recommended to assess the impact of a biologic on exacerbations and asthma control; in the event of no response, switching to another biologic can be considered.*

***Quality of evidence: high. Strength of recommendation: A***

### 3.9. PICO Question 9. In Children with Asthma, Does Vitamin D Supplementation Help with Asthma Control?

#### 3.9.1. Executive Summary

Despite previous evidence [152], the most updated Cochrane review on the role of vitamin D in reducing the risk of asthma exacerbation and improving asthma control did not find significant evidence [153].

Over the last few years, several RCTs have been published. Eighty-four asthmatic children aged 3–18 years with vitamin D deficiency were randomized to receive treatment with vitamin D or placebo. The study demonstrated no significant differences in respiratory resistance or reaction after 3 months of supplementation [154]. In the Vitamin D Kids Asthma Study, vitamin D supplementation (4000 IU) was not significantly associated with changes in lung function measures, asthma control, or asthma-related quality of life [155]. Even when administered to high-risk children (1 severe exacerbation in the previous year), vitamin D does not reduce the risk of future severe asthma exacerbations [156]. Vitamin D supplementation did not improve asthma control measured by ACT score or exacerbations in two recent RCTs [157,158].

Slightly positive results come from a very recent study where children with severe asthma had lower mean vitamin D levels than those with mild/moderate asthma, and vitamin D was positively correlated with FEV_1_. However, vitamin D levels were not associated with asthma control [159].

A systematic review, including studies published until 2021 (n = 15), reported no association between vitamin D status and asthma control [160].

#### 3.9.2. Recommendation

*Vitamin D supplementation is not associated with improvements in asthma control or lung function.*

***Quality of evidence: low. Strength of recommendation: C***

### 3.10. PICO Question 10. In Children with Asthma, Does Influenza Vaccination Help with Asthma Control?

#### 3.10.1. Executive Summary

Children with asthma constitute a high-risk population for complications from influenza [161]. There is evidence to suggest that influenza vaccination is protective against asthma exacerbations [162].

The analysis of data from the United States covering the period 2005–2018 showed that there were greater decreases in the odds of current asthma prevalence among children who were vaccinated compared with those who were not vaccinated for influenza, suggesting that vaccination may have contributed to the declining prevalence of asthma in American children [163]. A recent Spanish study including both children and adults estimated that current or previous influenza vaccination of people with asthma prevented almost half of the influenza cases [164].

There were concerns that live attenuated influenza vaccines such as the intranasally administered vaccines could expose children with wheezing or asthma to a higher risk of medically significant wheezing. Therefore, the Advisory Committee on Immunization Practices recommended against the vaccine in preschool children with a history of at least one wheezing episode in the past 12 months and for the vaccine to be used with caution in older children (≥5 years) with asthma [165]. A recent meta-analysis, including 14 studies published over 20 years and involving a total of 1.2 million participants, provided evidence that live attenuated influenza vaccines are well tolerated with no safety concerns in individuals aged 2–49 years with a diagnosis of asthma or recurrent wheezing [166]. In a recent American RCT, quadrivalent live attenuated influenza vaccine was not associated with an increased frequency of asthma exacerbations compared with quadrivalent inactivated influenza vaccine among children aged 5 to 17 years with asthma [167].

#### 3.10.2. Recommendation

*Influenza vaccination is strongly recommended for pediatric patients with asthma to help prevent severe influenza-related complications.*

***Quality of evidence: moderate. Strength of recommendation: A***

All recommendations are summarized in Table 1.

## 4. Discussion

Asthma management relies on symptom relief and long-term control. Long-term control is focused on preventing symptoms and depends on asthma maintenance therapy to reduce airway inflammation. Controller medications include ICS, considered the first-line treatment for asthma maintenance therapy; leukotriene modifiers; LABA (salmeterol and formoterol); and LAMA (tiotropium). Biologics are reserved for severe asthma. The choice of medication depends on the severity of the child’s asthma, phenotype, age, preference, and individual factors. The initial assessment of the patient must always be followed by regular reassessments to check asthma control and treatments’ effects.

Since severe attacks can also happen in patients with mild asthma, before considering a regimen with SABA as needed, an assessment of control based on the severity and frequency of symptoms, limitations of activities, and use of SABA is crucial. When choosing the regimen with ICS daily treatment, the clinician has to confirm whether the patient is likely to be adherent with maintenance therapy because a non-adherent patient, especially in adolescence, will be exposed to the risks of SABA-only treatment and therefore severe exacerbations. Especially in these patients, who are likely to be poor adherents to daily treatment when they do not have symptoms, formoterol/ICS as needed can be a valid alternative since the single inhaler formoterol/ICS will be used to relieve symptoms and at the same time reduce lung inflammation. Daily ICS are the preferred starting option in patients with frequent symptoms since there is poor evidence in favor of daily LTRA.

In persistent asthma, SMART therapy with a single inhaler is widely considered the preferred approach, particularly in adolescents. When symptoms are not controlled despite daily treatment, several options are suggested, including adding LAMA or a biologic. These steps of treatment require tertiary care in a severe asthma clinic. SLIT and SCIT AIT can improve asthmatic symptoms and quality of life and reduce the use of short- and long-term medications.

In addition to medications, identification of comorbidities such as obesity, rhinitis, or gastroesophageal reflux and modifiable factors such as inhaler technique and adherence to therapy are crucial to obtaining good control. Furthermore, risk factors for exacerbations must be taken into account (recent exacerbations, poor inhalation technique, poor adherence, and comorbidities) to choose the best therapeutic regimen. The final aim of each asthma plan is to develop individualized treatments that are tailored to the child’s specific needs. In addition, influenza vaccination represents a relevant preventive approach in order to avoid respiratory complications in children with asthma.

## 5. Conclusions

Our study provides evidence-based recommendations to guide clinicians on maintenance therapy for children and adolescents with asthma. Asthma in children is heterogeneous, and its evolution varies over time. In addition, the assessment of phenotypes and endotypes is difficult since bronchoscopy is rarely performed, and obtaining samples might be tricky in young children. Since most recommendations for asthma management in childhood are extrapolated from clinical studies performed in adults, more clinical trials specifically designed for young children should be conducted.

## Figures and Tables

**Table 1:** Summary of PICO questions and recommendations.

	Quality of Evidence	Strength of Recommendation
**PICO question 1.** *In children with infrequent symptoms, is SABA combined with ICS or, as needed, ICS-formoterol preferred to SABA alone?*		
For young children aged 6–12 years with infrequent symptoms (step 1), as needed, low-dose ICS associated with SABA for symptom relief is suggested.	Low	B
For adolescents aged >12 years with infrequent symptoms (step 1), low-dose ICS associated with SABA or low-dose ICS-formoterol are the preferred strategies.	Moderate	B
**PICO question 2.** *In children and adolescents with asthma, is daily therapy with ICS more effective than daily LTRA?*		
ICS should be the first-line treatment in children with mild to moderate persistent asthma, particularly in those with features suggesting Th_2_ inflammation.	High	A
ICS are more effective than LTRA.	Moderate	A
Due to its simplicity of administration, a trial with LTRA may be considered an alternative to ICS in patients with poor adherence to treatment or who have difficulty using inhalers. LTRA is also considered an add-on therapy when daily ICS cannot provide adequate symptom control.	Low	C
**PICO question 3**. *In children with uncontrolled asthma symptoms despite low daily ICS, is increasing the dose of ICS more effective than adding LABA or LTRA?*		
In adolescents >12 years with moderate persistent asthma (step 3 or 4), despite the assessment of comorbidities and modifiable factors, a SMART approach with ICS/formoterol as maintenance and reliever therapy is the preferred option. ICS/LABA as maintenance and SABA as a reliever are valid alternatives; however, adherence to therapy must be checked since there is a higher risk of SABA overuse.	High	A
In children 6 to 11 < 12 years, with moderate persistent asthma (step 3 or 4), despite the assessment of comorbidities and modifiable factors, a SMART approach with ICS/formoterol as maintenance and reliever therapy (with a check of inhalation technique) and ICS/LABA as maintenance and SABA as a reliever are both valid alternatives; increasing the ICS dose can be considered.	Moderate	B
**PICO question 4.** *In children with uncontrolled asthma symptoms despite daily therapy, what is the preferred option between increasing the therapy or assessing modifiable factors (adherence, inhalation technique, exposure to allergens)?*		
In patients with uncontrolled asthma despite treatment, before stepping up therapy, increasing the ICS dose, or adding a new medication, modifiable factors, and comorbidities must be reassessed. If modifiable factors are identified and addressed but asthma control remains poor, increasing therapy may be necessary.	Low	B
**PICO question 5.** *In children with asthma, is a MD) preferred to a DPI?*		
Currently, there is no clear opinion on the superiority of one inhaler over the other. The decision is based on the patient’s needs, abilities, and preferences. Accurate description of the devices and demonstration of inhalation techniques are mandatory in asthma clinics to provide the patient and the family with the correct information to take the medication. DPI may be reserved for older children aged at least 10 years.	Low	C
**PICO question 6.** *Which patients with asthma can benefit from immunotherapy?*		
AIT is effective in the pediatric population with IgE-mediated allergic respiratory diseases. Candidates for AIT are patients with mild to moderately controlled asthma who need long-lasting or multiple drugs to maintain asthma control. Both SLIT and SCIT AIT are beneficial in improving asthmatic symptoms and quality of life and reducing the use of short- and long-term medications.	Moderate	B
**PICO question 7.** *In children with uncontrolled asthma symptoms despite daily medium-dose ICS combined with LABA, is increasing the dose of ICS more effective than adding the LAMA tiotropium?*		
In children and adolescents with moderate to severe asthma, both increasing the dose of ICS+LABA and adding tiotropium in a separate inhaler can be effective in terms of asthma control, reduction of exacerbations, and lung function improvement.	Moderate	B
**PICO question 8.** *Considering the biologics for severe asthma, which are the differences between omalizumab, mepolizumab, and dupilumab?*		
All biologics for children >6 years with asthma target Th_2_ inflammation. Biomarkers can be predictive of treatment response to biologics. In severe allergic asthma (high IgE), omalizumab can always be chosen. If the patient shows high FeNO and high blood eosinophils, mepolizumab or dupilumab can be considered (regardless of IgE). All biologics reduce severe exacerbation rates; however, only dupilumab seems to be associated with improvements in lung function. If a patient with moderate to severe asthma is affected by moderate to severe atopic dermatitis or eosinophilic esophagitis, dupilumab might be the best choice. On the contrary, if the patient suffers from chronic spontaneous urticaria, they may likely benefit from omalizumab. Although all are administered subcutaneously, the number and frequency of injections vary substantially between biologics. A trial of 4 to 6 months is recommended to assess the impact of a biologic on exacerbations and asthma control; in the event of no response, switching to another biologic can be considered.	High	A
**PICO question 9.** *In children with asthma, does vitamin D supplementation help with asthma control?*		
Vitamin D supplementation is not associated with improvements in asthma control or lung function.	Low	C
**PICO question 10.** *In children with asthma, does influenza vaccination help with asthma control?*		
Influenza vaccination is strongly recommended for pediatric patients with asthma to help prevent severe influenza-related complications.	Moderate	A

AIT, allergen immunotherapy; DPI, dry powder inhaler; FeNO, fractional exhaled nitric oxide; ICS, inhaled corticosteroids; LABA, long-acting bronchodilators; LTRA, leukotrience receptor antagonist; MDI, metered dose inhaler; PICO, Patient, Intervention, Comparison and Outcome; SABA, short-acting bronchodilators; SLIT, sublingual immunotherapy.

## Data Availability

Not applicable.

## Supplementary Materials

The following supporting information can be downloaded at: https://www.mdpi.com/article/10.3390/jcm12175467/s1, Supplementary Material File S1: Clinical questions and PICO items.
