# Supplementary material for: Maintenance Therapy for Children and Adolescents with Asthma: Guidelines and Recommendations from the Emilia-Romagna Asthma (ERA) Study Group
Source: J Clin Med. 2023 Aug 23;12(17):5467. doi: 10.3390/jcm12175467 (PMC10487522; doi:10.3390/jcm12175467)
Supplement: Supplementary file 1 [file jcm-12-05467-s001.zip › jcm-2475284-supplementary.pdf]

Supplementary material

Clinical questions and PICO items

PICO question 1. In children with mild asthma and occasional symptoms, is short-acting beta2agonists (SABA) combined to inhaled corticosteroids (ICS) or as-needed inhaled corticosteroids (ICS)-formoterol preferred to SABA alone?

Patient or population: children and adolescents with asthma
Setting: primary to tertiary care
Intervention: as needed ICS-formoterol or ICS plus SABA
Comparison: SABA alone
Outcome: symptom control and exacerbations

Search strategy:
"Asthma"[Mesh] OR "asthma" AND ("Anti-Asthmatic Agents"[Mesh] OR "Adrenergic beta-2 Receptor Agonists"[Mesh] OR "Bronchodilator Agents"[Mesh] OR "albuterol"[MeSH Terms] OR "salbutamol" OR "formeterol" OR "SMART therapy" OR "Adrenal Cortex Hormones"[Mesh] OR "inhaled steroid*")
Filters applied: last 5 years, Child: 6-12 years, Adolescent: 13-18 years

PICO n° 1 Workflow of study selection

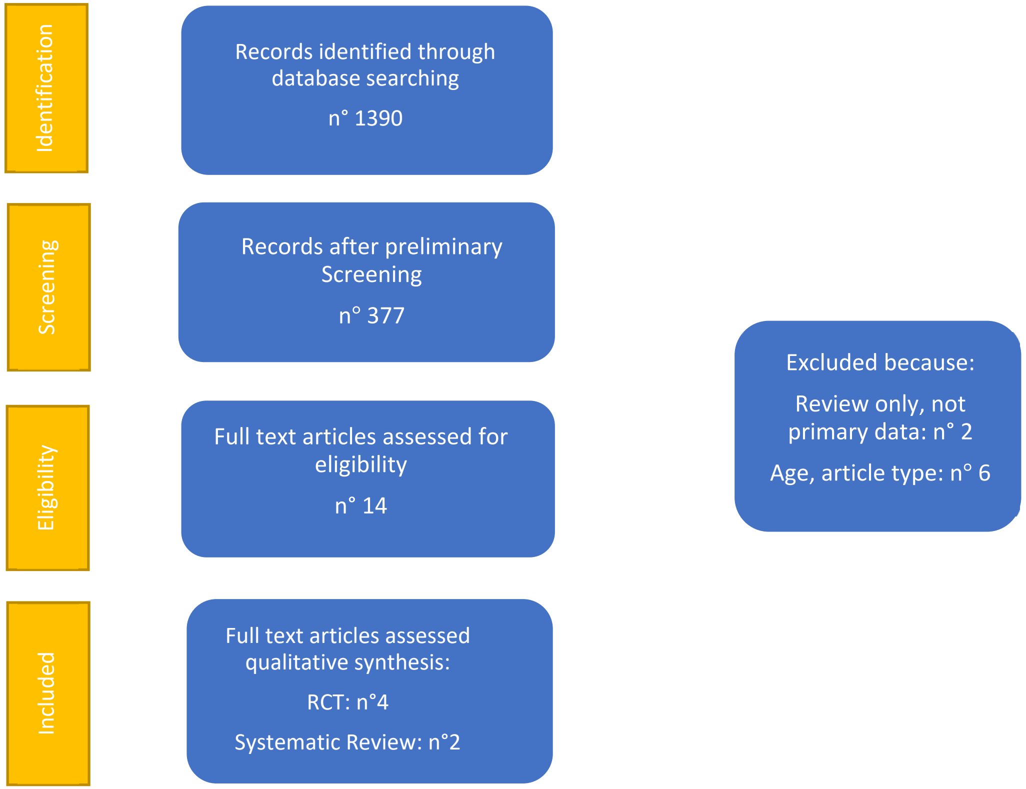

Modified from: Moher D, Liberati A, Tetzlaff J, Altman DG, The PRISMA Group (2009).
Preferred Reporting Items for Systematic Reviews and Meta-Analyses: The PRISMA Statement. PLoS Med 6(7): e1000097

Title of the study, first author, year [ref]	Type of study	Study design	Population	N of patients, (age)	Outcomes	Results	
Efficacy and Safety of As-Needed Budesonide-Formoterol in Adolescents with Mild Asthma. Reddel HK, 2021 [1]	Randomized Controlled Trial	Patient were randomised to twice-daily placebo + as-needed BUD-FORM, twice-daily BUD +as-needed terbutaline (BUD maintenance) or twice-daily placebo + as-needed terbutaline	Paediatric patients diagnosed with mild asthma	889 (12-18 years)<br><br>as-needed terbutaline (n 144), as-needed BUD-FORM (n 366), or BUD maintenance (n 379)	Annual severe exacerbations	In SYGMA 1, the annualised rate of severe exacerbations in adolescents was 77% lower with as-needed BUD-FORM versus as-needed terbutaline.	0.04 vs 0.17; RR 0.23; 95% CI, 0.09 to 0.65; P=.005
					Time to first severe exacerbation	Significantly longer with as-needed BUD-FORM versus as-needed terbutaline	Hazard ratio, 0.33; 95% CI,<br>0.13 to 0.85; P= .02)
						Not significantly different between as-needed BUD-FORM and BUD maintenance	Hazard ratio, 1.23; 95% CI, 0.70 to 2.18; P= .47
					Lung function (FEV_1% predicted)	No statistically significant difference between as-needed BUD-FORM and as-needed terbutaline in SYGMA 1.	0.9%; 95% CI, 1.1 to 2.8; P = .395
						A statistically significant difference in as-needed BUD-FORM versus BUD maintenance in SYGMA 1	3.9%; 95% CI, 5.8 to 1.9; P < .001
					ACQ-5 score	The change from baseline in ACQ-5 score was significantly greater with as-needed BUD-FORM compared with as-needed terbutaline but this difference did not reach the minimal clinically important difference of 0.5	0.17; 95% CI, 0.30 to 0.03; P =.02
					Adherence	No statistically significant	
					Adverse events	Higher with as-needed<br>terbutaline (41.0%), primarily due to asthma-related events.	

As-Needed Use of Short-Acting β2-Agonists Alone Versus As-Needed Use of Short-Acting β2-Agonists Plus Inhaled Corticosteroids in Pediatric Patients With Mild Intermittent (Step 1) Asthma: A Cost-Effectiveness Analysis. Rodriguez-Martinez CE, 2022 [2]	Randomized Controlled Trial	Evaluation of the cost-effectiveness of the as-needed use of SABAs alone versus the as-needed use of SABAs plus ICS	Asthmatic patients with acute exacerbation	5-11 years	First course of prednisone for an asthma exacerbation (AE)	Lower overall treatment costs and a higher probability of a lack of a requirement for a first course of prednisone	0.6500 vs 0.5100
Inhaled Combined Budesonide-Formoterol as Needed in Mild Asthma. O'Byrne, 2018 [3]	Double-blind, randomized, parallel-group, 52-week, phase 3 trial	Evaluation the efficacy and safety of budesonide-formoterol (200 µg of budesonide and 6 µg of formoterol) used as needed, as compared with terbutaline (0.5 mg; terbutaline) used as needed and with twice-daily budesonide (200 µg;)	Patients 12 years of age or older with mild asthma	3849 (n=478 <18 yrs)<br><br>1280 (n=144 <18 yrs) terbutaline<br><br>1279 (n=161 <18 yrs) budesonide-formoterol group<br><br>1290 (n=173 <18 yrs) to budesonide	Asthma control symptoms<br><br>Rates and time to first moderate to severe exacerbation between budesonide–formoterol used as needed vs budesonide maintenance therapy and vs terbutaline group<br><br>ACQ-5 score	Budesonide–formoterol used as needed was superior to terbutaline used as needed<br><br>Budesonide–formoterol used as needed resulted in a 64% lower rate of severe exacerbations than terbutaline used as needed<br><br>The rates of severe exacerbations in the budesonide–formoterol group and the budesonide maintenance group did not differ significantly<br><br>Budesonide–formoterol used as needed also resulted in a 60% lower rate of moderate-to-severe exacerbations than terbutaline used as needed<br><br>Change from baseline in the ACQ-5 score in favour of the budesonide–formoterol group versus the terbutaline group	34.4% vs. 31.1% of weeks; odds ratio, 1.14; 95% confidence interval [CI], 1.00 to 1.30; P=0.046<br><br>0.07 vs. 0.20; rate ratio, 0.36; 95% CI, 0.27 to 0.49)<br><br>0.07 and 0.09; rate ratio, 0.83; 95% CI, 0.59 to 1.16<br><br>0.14 vs. 0.36<br><br>−0.15; 95% CI, −0.20 to −0.11

		plus terbutaline (0.5 mg) used as needed		maintenance group	Change of baseline FEV_1 before bronchodilator	65.0 ml in the budesonide–formoterol group vs. 11.2 ml in the terbutaline group	95% CI, 47.6 to 82.4<br>95% CI, −6.4 to 28.9
As-Needed Budesonide-Formoterol versus Maintenance Budesonide in Mild Asthma.<br>Bateman ED, 2018 [4]	Double-blind, randomized, parallel-group, phase 3 trial	Twice-daily placebo plus budesonide–formoterol (200 µg of budesonide and 6 µg of formoterol, n= 2089) used as needed vs budesonide maintenance therapy with twice-daily budesonide (200 µg) plus terbutaline (0.5 mg) used as needed (n=2087)	Mild asthma	4215 (12-83 years old)	Evaluation whether budesonide–formoterol used as needed was noninferior to budesonide maintenance therapy in terms of the annualized rate of severe exacerbations	Budesonide–formoterol used as needed was noninferior to budesonide maintenance therapy.<br><br>Annualized rate 0.11 (95% CI 0.10 to 0.13) in the budesonide– formoterol group and 0.12 (95% CI 0.10 to 0.14) in the budesonide maintenance group.	Rate ratio 0.97 (one-sided 95% upper confidence limit, 1.16)
					N severe exacerbations	No difference	HR 0.96 (95% CI, 0.78 to 1.17).
					Steroid dose	The median daily metered dose of inhaled glucocorticoid was lower in the budesonide–formoterol group (66 µg) than in the budesonide maintenance group (267 µg).<br>A mean of 0.52±0.55 inhalations per day of budesonide–formoterol was used as needed, as compared with 0.49±0.70 inhalations per day of terbutaline used as needed in the budesonide maintenance group.<br>Difference of 0.11 units in favor of budesonide maintenance therapy.	(95% CI, 0.07 to 0.15)
					The forced expiratory volume in 1 second (FEV_1) before bronchodilator use, trialspecific asthma-related discontinuation	The change from baseline in the FEV_1 both before and after bronchodilator use was less in the budesonide–formoterol group than in the budesonide maintenance group (mean difference in FEV_1 before bronchodilator use, −32.6 ml; mean difference in FEV_1 after bronchodilator use, −23.1 ml)	[95% CI, −53.7 to −11.4]<br><br>[95% CI, −41.9 to −4.2]

					Use of maintenance therapy and as-needed reliever therapy and the percentage of reliever-free days<br><br>Score on the Asthma Control Questionnaire–5 (ACQ-5), and score on the standardized Asthma Quality of Life Questionnaire (AQLQ)	Fewer patients in the budesonide–formoterol group than in the budesonide maintenance group used more than 8 inhalations of the as-needed agent per day (10.4% vs. 15.0%) or more than 12 inhalations per day (4.1% vs. 7.4%) at least once<br><br>The decrease in the budesonide– formoterol group was less than in the budesonide maintenance group (mean difference, 0.11 units), and fewer patients in the budesonide–formoterol group than in the budesonide maintenance group had a decrease from baseline in the ACQ-5 score of at least 0.5 units (40.3% vs. 44.3%).<br>The change in the AQLQ overall score was less in the budesonide–formoterol group than in the budesonide maintenance group (mean difference, −0.10).	95% CI, 0.07 to 0.15<br><br>Odds ratio, 0.86; 95% CI, 0.75 to 0.99<br><br>95% CI, −0.14 to −0.05

BUD-FORM: Budesonide-Formoterol, OR: odds ratio, RR relative risk, CI: confidence interval, HR: hazard ratio, FEV1: forced expiratory flow in 1 second, ACQ-5 score: Asthma Control Questionnaire-5 score, AQLQ: Asthma Quality of Life Questionnaire, PAQLQ: Paediatric Asthma Quality of Life Questionnaire, PIS: pulmonary index score, ED: emergency department, SABA: short acting beta-2 agonist, ICS: inhaled corticosteroids, AE: asthma exacerbation, MART: maintenance and reliever therapy

References

1. Reddel HK, O'Byrne PM, FitzGerald JM, Barnes PJ, Zheng J, Ivanov S, Lamarca R, Puu M, Alagappan VKT, Bateman ED. Efficacy and Safety of As-Needed Budesonide-Formoterol in Adolescents with Mild Asthma. J Allergy Clin Immunol Pract. 2021 Aug;9(8):3069-3077.e6. doi: 10.1016/j.jaip.2021.04.016. Epub 2021 Apr 22. PMID: 33895362.

2. Rodríguez-Martínez CE, Sossa-Briceño MP, Buendia JA. As-Needed Use of Short-Acting β2-Agonists Alone Versus As-Needed Use of Short-Acting β2-Agonists Plus Inhaled Corticosteroids in Pediatric Patients With Mild Intermittent (Step 1) Asthma: A Cost-Effectiveness Analysis. J Allergy Clin Immunol Pract. 2022 Jun;10(6):1562-1568. doi: 10.1016/j.jaip.2022.02.022. Epub 2022 Mar 5. PMID: 35259534.
3. O'Byrne PM, FitzGerald JM, Bateman ED, Barnes PJ, Zhong N, Keen C, Jorup C, Lamarca R, Ivanov S, Reddel HK. Inhaled Combined Budesonide-Formoterol as Needed in Mild Asthma. N Engl J Med. 2018 May 17;378(20):1865-1876. doi: 10.1056/NEJMoa1715274. PMID: 29768149.
4. Bateman ED, Reddel HK, O'Byrne PM, Barnes PJ, Zhong N, Keen C, Jorup C, Lamarca R, Siwek-Posluszna A, FitzGerald JM. As-Needed Budesonide-Formoterol versus Maintenance Budesonide in Mild Asthma. N Engl J Med. 2018 May 17;378(20):1877-1887. doi: 10.1056/NEJMoa1715275. PMID: 29768147.

PICO question 2. In children with asthma, is daily therapy with ICS more effective than daily leukotriene receptor antagonist (LTRA)?

Patient or population: children and adolescents with asthma

Setting: primary to tertiary care

Intervention: daily ICS

Comparison: daily LTRA

Outcome: symptom control

Search strategy:

"Asthma"[Mesh] OR "asthma" AND ("Anti-Asthmatic Agents"[Mesh] OR "Adrenal Cortex Hormones"[Mesh] OR "inhaled steroid*" OR "Leukotriene Antagonists"[Mesh] OR "montelukast")

Filters applied: last 5 years, Child: 6-12 years, Adolescent: 13-18 years

PICO n°_2__Workflow of study selection process

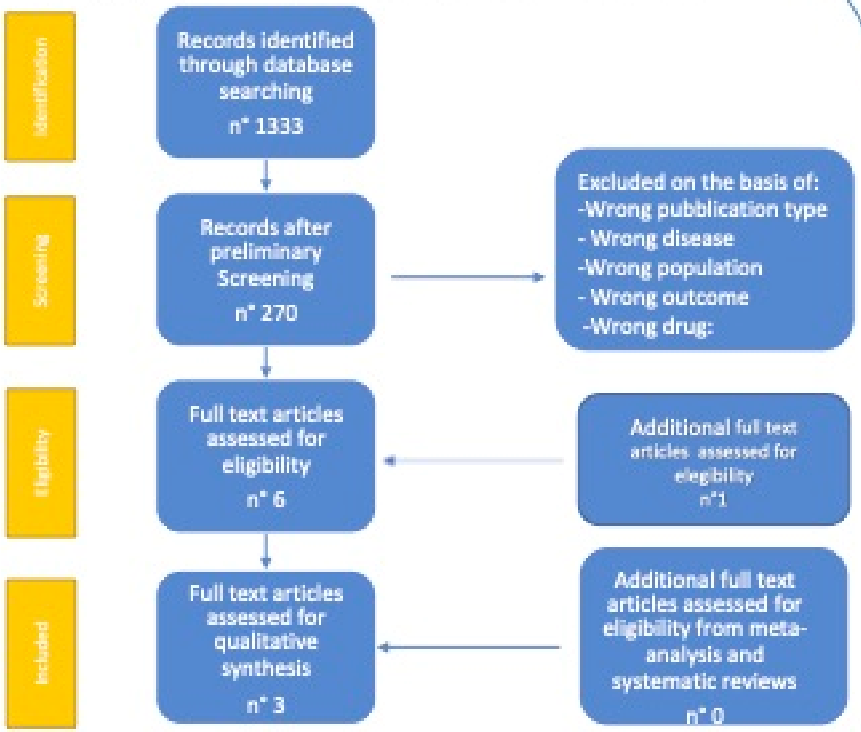

Modified from: Moher D, Liberati A, Tetzlaff J, Altman DG, The PRISMA Group (2009). Preferred Reporting Items for Systematic Reviews and Meta-Analyses: The PRISMA Statement. PLoS Med 6(7): e1000097

Title of the study, first author, year	Type of study	Study design	Population	N of patients (age)	Methods	Outcomes
Comparative outcomes between inhaled budesonide and oral montelukast in mild persistent childhood asthma, Sushant Mane et al. 2019 [1]	Randomized prospective parallel-group comparative study	Group A receiving Montelukast (4-5 mg once a day) vs Group B receiving inhaled Budesonide (100 mcg twice a day) for 3 months	Children with mild persistent asthma	54 children: group A (n=28); group B (n=26) (3-12 years)	Asthma control symptoms<br><br>Improvement in day-time symptoms<br>Asthma exacerbation	Asthma control was better in Group B vs Group A: lesser episodes of night awakening (23.1% vs 53.6%; p=0.028); fewer reliever medications (26.9% vs 60.7%; p=0.016).<br>Better improvement in control of asthma symptoms in Group B at 3 months than at 4 weeks (B p=0.046 vs A p=0.09).<br>Asthma exacerbation:<br>better control in Group A vs Group B at 4 weeks for viral infection triggered- exacerbations (p=0.045); no statistically significant difference at 3 months.
Comparative effectiveness of budesonide inhalation suspension and montelukast in children with mild asthma in Korea, Jina Shin et al., 2020 [2]	Retrospective, observational cohort study	Montelukast (MON) vs low-dose Budesonide inhalation suspension (BIS, ≤ 500 mcg budesonide per day) monotherapy	Children with mild persistent asthma (GINA 1 or 2)	-26052 for unmatched (n=1221 BIS; n=24831 MON) - 2290 for matched populations (n=1145 per cohort).<br><br>(2-17 years)	Data collected from the Health Insurance Review and Assessment (HIRA) Service Assessment of treatment adherence, treatment persistency, asthma control, health resource utilization, costs	Medication adherence was significantly higher for MON vs BIS (13.8% vs. 4.5%; p < 0.001).<br>Time to loss of persistency: longer for MON vs BIS (82.3 vs. 78.4 days, respectively; p<0.001).<br>Dose escalation of the index therapy: higher in the BIS cohort than MON (12.1% vs. 1.0%; p<0.001).<br>Mean number of asthma-related office visits: lower for BIS vs MON (6.6 vs 8.3; p < 0.001).<br>Asthma exacerbation-related office visit: greater for BIS than the MON cohort (78.3% vs. 56.1%; p<0.001).<br>Asthma-related total health-care costs: higher with MON vs BIS (₩ 190,185 vs. ₩ 167,432; p<0.001).
Treatment of pediatric mild persistent	Retrospective, observatio	Montelukast (MON, 4-5 mg	Children with mild persistent asthma	393 children (2–14 years): BIS (*n* = 153) vs	Data derived from a retrospective questionnaire-based analysis.	Medication compliance: better in the montelukast group than in the BIS group (*P* = 0.042).

asthma with low-dose budesonide inhalation suspension vs. montelukast in China, Zhi-Min Chen, 2021 [3]	nal cohort study	once daily) vs low-dose Budesonide inhalation suspension (BIS, 500 mcg per day) monotherapy.		Montelukast (*n* = 240).	Indicators of asthma control:<br><br>Asthma Control Test (ACT), Childhood ACT (C-ACT) score.<br>Asthma-related medical costs.	Asthma control better in the montelukast group: lower percentages of asthmatic children with symptoms more than twice a week ($p = 0.021$), having night waking or night coughing ($p = 0.022$), required reliever medication more than twice a week ($p < 0.001$).<br>ACT/C-ACT score: better in the montelukast group ($p = 0.015$).<br>Caregivers-reported exercise tolerance: better in montelukast group vs. BIS group ($p< 0.001$).<br>Medical costs: significantly higher in the BIS group vs. montelukast group ($p < 0.001$).

References

1. Mane DS, Mane S, Rampur PP, Rajagara SS. Comparative outcomes between inhaled budesonide and oral montelukast in mild persistent childhood asthma. International Journal of Medical and Biomedical Studies. 2019. doi:10.32553/ijmbs.v3i9.544

2. Shin J, Oh SJ, Petigara T, Tunceli K, Urdaneta E, Navaratnam P, Friedman HS, Park SW, Hong SH. Comparative effectiveness of budesonide inhalation suspension and montelukast in children with mild asthma in Korea. J Asthma. 2020 Dec;57(12):1354-1364. doi: 10.1080/02770903.2019.1648504. Epub 2019 Aug 6. PMID: 31386600.

3. Chen ZM, Zhao DY, Xiang L, Hong JG. Treatment of pediatric mild persistent asthma with low-dose budesonide inhalation suspension vs. montelukast in China. World J Pediatr. 2021 Dec;17(6):619-625. doi: 10.1007/s12519-021-00464-7. Epub 2021 Oct 6. PMID: 34613593.

PICO question 3. In children with uncontrolled asthma symptoms despite daily ICS, is increasing the dose of ICS more effective than adding long-acting beta2agonists (LABA) or LTRA?

Patient or population: children and adolescents with asthma
Setting: primary to tertiary care
Intervention: increasing ICS
Comparison: addition of LABA or LTRA
Outcome: symptom control and exacerbation

Search strategy:
"Asthma"[Mesh] OR "asthma" AND ("Anti-Asthmatic Agents"[Mesh] OR "Adrenergic beta-2 Receptor Agonists"[Mesh] OR "Bronchodilator Agents"[Mesh] OR "Adrenal Cortex Hormones"[Mesh] OR "inhaled steroid*" OR "Leukotriene Antagonists"[Mesh] OR "montelukast" OR "LABA" OR "salmeterol")
Filters applied: last 5 years, Child: 6-12 years, Adolescent: 13-18 years

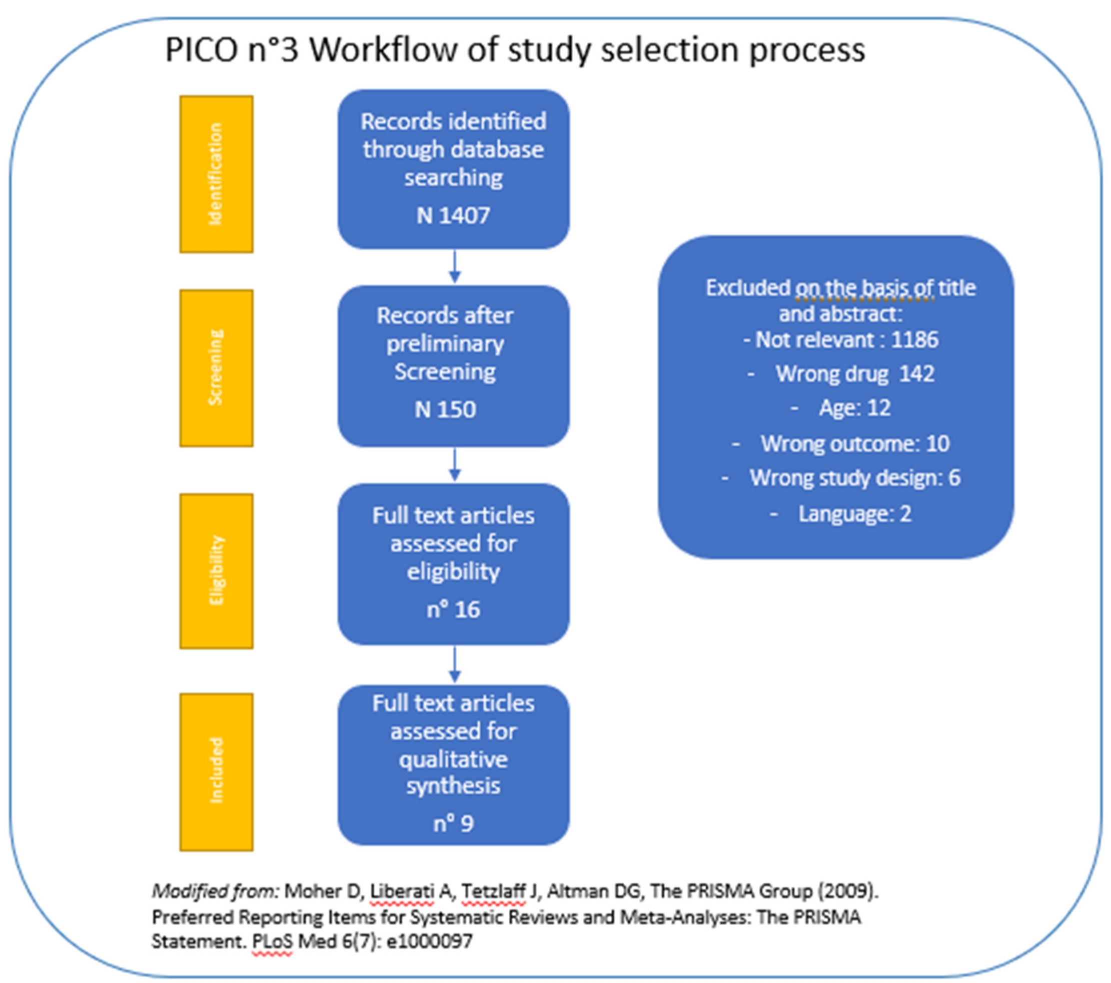

PICO n°3 Workflow of study selection process
Identification
Screening
Eligibility
Included
Records identified through database searching
N 1407
Records after preliminary Screening
N 150
Full text articles assessed for eligibility
n° 16
Full text articles assessed for qualitative synthesis
n° 9
Excluded on the basis of title and abstract:
- Not relevant : 1186
- Wrong drug 142
- Age: 12
- Wrong outcome: 10
- Wrong study design: 6
- Language: 2
Modified from: Moher D, Liberati A, Tetzlaff J, Altman DG, The PRISMA Group (2009). Preferred Reporting Items for Systematic Reviews and Meta-Analyses: The PRISMA Statement. PLoS Med 6(7): e1000097

Title of the study, first author, year [ref]	Type of study	Study design	Population	N of patients, age	Methods	Outcomes	
Efficacy and safety of fluticasone propionate/formoterol fumarate in pediatric asthma patients: a randomized controlled trial. Płoszczuk A, 2018 [1]	Randomized Controlled Trial	Fluticasone MDIP (100 µg twice daily, n=173) vs fluticasone/formoterol MDIP(100/10 µg twice daily, n=169) vs fluticasone/salmeterol MDIP (100/50 µg twice daily, n=170). All patients received two inhalers during the treatment period: active or placebo fluticasone and a corresponding active or placebo ICS/LABA (fluticasone/formoterol or fluticasone/salmeterol)	Persistent asthma ⩾ 6 months and (FEV_1) ⩽ 90% predicted	512 (5-12 years)	Primary endpoint: change from predose FEV_1 at baseline to 2 h post-dose FEV_1 over 12 weeks treatment period.	Fluticasone/formoterol was superior to fluticasone.	LS mean difference 0.07 l; 95% confidence interval (CI) 0.03, 0.11; p< 0.001.
					Secondary endpoints: FEV_1 area under the curve (AUC 0–4h) at week 12.	Fluticasone/formoterol was non-inferior to fluticasone/salmeterol.	LS mean difference 0.00 l; 95% CI −0.04, 0.04; p< 0.001.
					Change from predose FEV_1 at baseline to predose FEV_1 over the 12-week treatment period.	Fluticasone/formoterol was superior to fluticasone.	LS mean difference 0.09 l; 95% CI: 0.04, 0.13; p< 0.001.
						Fluticasone/formoterol was non-inferior to fluticasone/salmeterol.	LS mean difference 0.01; 95% CI −0.03, 0.06; p< 0.001.
						Difference not statistically significant	LS mean difference 0.03 l; 95% CI −0.01, 0.07; p= 0.091
						Fluticasone/formoterol was non-inferior to fluticasone/salmeterol.	Treatment difference −0.02 l; 95% CI −0.06, 0.02; p< 0.001
Budesonide/formoterol maintenance and reliever therapy in adolescent patients with asthma.	Post hoc analysis of six randomized controlled trials	Budesonide/formoterol maintenance and reliever therapy (n=694) vs budesonide plus terbutaline	Persistent asthma ⩾ 6 months	1847 (12 – 17 years)	Primary endpoint: time to first severe exacerbation.	Budesonide/formoterol maintenance and reliever therapy was similar to or more effective than comparator.	HR numerically favored budesonide/formoterol maintenance and reliever therapy for all treatment comparisons in five

Jorud C, 2018 [2]		(n=225) or budesonide/formoterol plus terbutaline (n=441) or Budesonide/formoterol plus formoterol (n=115) or salmeterol/fluticasone plus terbutaline (n=372)					of the six studies (ranging from 0.15 to 0.93) and were similar for budesonide/formoterol maintenance and reliever therapy and comparator (budesonide/formoterol + formoterol as needed) in the remaining study (HR 1.01)
					<u>Secondary endpoints:</u> total number of severe exacerbations, asthma symptom scores, night-time awakenings, as-needed inhalations, FEV_1, morning PEF and ACQ-5 score.	Pooled estimates were in favor of budesonide/formoterol maintenance and reliever therapy.	
					ICS use	The budesonide/formoterol maintenance and reliever therapy arms received a lower mean daily ICS dose than the comparator arms in four studies.	
					Safety	The incidence of adverse events and the types of adverse events reported were similar for adolescents receiving budesonide/formoterol maintenance and reliever therapy and those receiving comparator treatments	
As-Needed Budesonide-Formoterol versus Maintenance Budesonide in Mild Asthma.	Double-blind, randomized, parallel-group, phase 3 trial	Twice-daily placebo plus budesonide–formoterol (200 µg of budesonide and 6 µg of	Mild asthma	4215 (12-83 years old)	<u>Primary endpoint</u>: rate of severe asthma exacerbations.	Budesonide–formoterol used as needed was noninferior to budesonide maintenance therapy.	Annualized rate 0.11 (95% CI 0.10 to 0.13) in the budesonide–formoterol group and 0.12 (95% CI 0.10 to 0.14) in the

Bateman ED, 2018 [3]		formoterol, n= 2089) used as needed vs budesonide maintenance therapy with twice-daily budesonide (200 µg) plus terbutaline (0.5 mg) used as needed (n=2087)			Secondary endpoints:<br>Severe exacerbations<br><br>Time of first exacerbation<br><br>Adherence and treatment exposure<br><br>As-Needed Medication<br><br>Change in ACQ-5 score	No significant differences in each group in number of patients with severe exacerbations that led to an emergency department visit or hospitalization, in the time to the first severe asthma exacerbation nor in the rate of severe exacerbations.<br><br>Similar in the two groups<br><br>The median daily metered dose of inhaled glucocorticoid was lower in the budesonide–formoterol group (66 µg) than in the budesonide maintenance group (267 µg).<br><br>A mean of 0.52±0.55 inhalations per day of budesonide–formoterol was used as needed, as compared with 0.49±0.70 inhalations per day of terbutaline used as needed in the budesonide maintenance group.<br><br>Difference of 0.11 units (95% CI, 0.07 to 0.15) in favor of budesonide maintenance therapy.	budesonide maintenance group.<br><br>Rate ratio 0.97 (one-sided 95% upper confidence limit, 1.16)<br><br>HR 0.96 (95% CI, 0.78 to 1.17).
Randomized, double-blind trial evaluating the efficacy and	Randomized controlled double-blind phase 3 trial	Fluticasone propionate MDPI 50 µg vs fluticasone	Persistent asthma	647 (n=86, 12-17 years)	Primary efficacy endpoints:<br>Change from baseline in trough (morning predose and pre-rescue bronchodilator) FEV_1 at week 12.	Significant improvements in Fluticasone propionate MDPI 50 and 100 µg and Fluticasone	

safety of fluticasone propionate and fluticasone propionate/salmeterol delivered via multidose dry powder inhalers in patients with persistent asthma aged 12 years and older.<br>Raphael G, 2018 [4]		propionate MDPI 100 µg, fluticasone propionate /salmeterol MDPI 50/12.5 µg vs fluticasone propionate/salmeterol MDPI 100/12.5 µg or placebo, all twice daily for12 weeks				propionate/salmeterol MDPI 50/12.5 and 100/12.5 µg treatment groups compared with the placebo group ($p<0.05$).<br><br>Significant improvement of the Fluticasone propionate/salmeterol MDPI 50/12.5 µg group compared with the Fluticasone MDPI 50 µg group ($p<0.05$).<br><br>Significant improvements of Fluticasone propionate/salmeterol MDPI 50/12.5 µg and 100/12.5 µg treatment groups compared with the Fluticasone propionate MDPI 100 µg treatment group ($p\leq 0.05$)	
					Standardized baseline-adjusted area under the effect curve for FEV_1 from time 0 to 12 hours after dosing (FEV_1AUEC 0–12 h) at week 12.	Significantly improved compared with placebo for the Fluticasone propionate MDPI 50 and 100 µg and Fluticasone propionate/salmeterol MDPI 50/12.5and 100/12.5 µg groups ($p\leq0.05$).<br><br>Significantly improved at week 12 in the Fluticasone propionate/salmeterol MDPI 50/12.5 µg group compared with the Fluticasone propionate MDPI 50 µg group ($p<0.05$) and in the Fluticasone propionate/salmeterol MDPI 50/12.5 and100/12.5 µg treatment groups compared with the Fluticasone propionate MDPI 100 µg treatment group ($p \leq0.05$).	

					Secondary efficacy endpoints:<br>Change from baseline in weekly average of the daily through morning PEF,	Statistically significant improvement in patients treated with fluticasone propionate/salmeterol MDPI 50/12.5 µg vs Fluticasone propionate MDPI 50 µg (p 0.0011) and vs Fluticasone propionate MDPI 100 µg (p 0.0175).<br><br>Statistically significant improvement in patients treated with fluticasone propionate/salmeterol MDPI 100/12.5 µg vs Fluticasone propionate MDPI 100 µg (p 0.0233).	
					Change from baseline in weekly averages of the daily asthma symptom score,	Not statistically significant differences in patients treated with fluticasone propionate/salmeterol MDPI 50/12.5 µg vs Fluticasone propionate MDPI 50 µg (p 0.243) and vs Fluticasone propionate MDPI 100 µg (p 0.509).<br><br>Not statistically significant differences in patients treated with fluticasone propionate/salmeterol MDPI 100/12.5 µg vs Fluticasone propionate MDPI 100 µg (p 0.138).	
					Change from baseline in weekly averages of the total daily use of albuterol/salbutamol.	Not statistically significant differences in patients treated with fluticasone propionate/salmeterol MDPI 50/12.5 µg vs Fluticasone propionate MDPI 50 µg (p 0.064) and vs Fluticasone propionate MDPI 100 µg (p 0.062).	

						Not statistically significant differences in patients treated with fluticasone propionate/salmeterol MDPI 100/12.5 µg vs Fluticasone propionate MDPI 100 µg (p 0.101).	
					Patient withdrawal due to worsening asthma symptoms	Not statistically significant differences in patients treated with fluticasone propionate/salmeterol MDPI 50/12.5 µg vs Fluticasone propionate MDPI 50 µg (p 0.993) and vs Fluticasone propionate MDPI 100 µg (p 0.999).<br><br>Not statistically significant differences in patients treated with fluticasone propionate/salmeterol MDPI 100/12.5 µg vs Fluticasone propionate MDPI 100 µg (p 0.313).	
					Proportion of patients achieving a 15% improvement from baseline in FEV_1 after dosing on day 1	Statistically significant improvement in patients treated with fluticasone propionate/salmeterol MDPI 50/12.5 µg vs Fluticasone propionate MDPI 50 µg ($p<0.0001$) and vs Fluticasone propionate MDPI 100 µg ($p<0.0001$).<br><br>Statistically significant improvement in patients treated with fluticasone propionate/salmeterol MDPI 100/12.5 µg vs Fluticasone propionate MDPI 100 µg (p 0.0115).<br><br>Statistically significant improvement in patients treated	

					Proportion of patients achieving a 12% improvement from baseline in FEV_1 after dosing on day 1<br><br>Safety	with fluticasone propionate/salmeterol MDPI 50/12.5 µg vs Fluticasone propionate MDPI 50 µg ($p<0.0001$) and vs Fluticasone propionate MDPI 100 µg ($p<0.0001$).<br><br>Statistically significant improvement in patients treated with fluticasone propionate/salmeterol MDPI 100/12.5 µg vs Fluticasone propionate MDPI 100 µg (p 0.0008).<br><br>Adverse events were similar across groups	
Efficacy of montelukast sodium chewable tablets combined with inhaled budesonide in treating pediatric asthma and its effect on inflammatory factors.<br>Zhang Y, 2019 [5]	Randomized Controlled Trial	Montelukast sodium (n=45) vs Budesonide (n=45) vs Combined Montelukast/Budesonide (n=45)	Asthmatic children	135 (3-12 years)	Disappearance time of symptoms<br><br>Pulmonary function: FVC, FEV_1, FEV_1/FVC and PEF<br><br>Inflammatory factors: TNF-α, IL-4, IL-8 and hs-CRP	Significantly shorter in the combined group than those in the single-drug group ($p <0.001$).<br>No difference between single use of montelukast and budesonide ($p > 0.05$).<br><br>No differences in the three groups before treatment.<br>After treatment, those in the combined group were significantly higher than those in the single-drug group ($p < 0.001$).<br><br>Before treatment, there was no difference among the three groups (all $p > 0.05$). After treatment, their expression levels in the combined group were significantly lower than those in the single-drug group, with statistical difference ($p < 0.05$).	

					Immune indices: number of CD4+, CD3+, CD8+ cells and IgE expression level<br><br>Adverse reactions<br><br>Incidence of asthma after treatment	No differences before treatments. After treatment, the number of CD4+ and CD3+ cells in the combined group was significantly higher than that in the single-drug group, while the number of CD8+ cells and IgE expression level were significantly lower (all $p < 0.05$).<br><br>No difference in the proportion of nausea, rash and headache among the groups during treatment (all $p > 0.05$).<br><br>In the combined group within 6 months was significantly lower than that in the single-drug group ($p < 0.05$).	
A phase 3 study evaluating the safety and efficacy of a pediatric dose of mometasone furoate with and without formoterol for persistent asthma. Weinstein CLJ, 2020 [6]	Phase 3, multicenter, randomized controlled trial	Mometasone furoate/Formoterol MDI 100/10 µg (n = 91) vs Mometasone furoate 100 µg (n = 90)	Persistent asthma	181 (5 – 11 years)	Primary endpoint: the change from baseline in AM post-dose 60-minute AUC % predicted FEV_1% across 12 weeks of treatment.<br><br>Secondary endpoints:<br>Change from baseline AM post-dose in %predicted FEV_1.<br><br>Percentage of subjects who increased SABA usage from baseline.<br><br>Reported Adverse Effect	Statistically significant overall treatment advantage with Mometasone furoate/Formoterol (7.20%) than Mometasone furoate (3.21%) (P< .001)<br><br>Significant improvement with Mometasone furoate/Formoterol MDI 100/10 µg on day 1 at 5 minutes, which was sustained through 4 hours post-dose (P< .001)<br><br>Lower for the Mometasone furoate/Formoterol group (26.4%) compared with the Mometasone furoate group (37.8%)	

						Fewer participants in the Mometasone furoate/Formoterol group (40.7%) compared with the Mometasone furoate group (57.8%)	
Once-daily mometasone plus indacaterol versus mometasone or twice-daily fluticasone plus salmeterol in patients with inadequately controlled asthma (PALLADIUM): a randomised, double-blind, triple-dummy, controlled phase 3 study.<br>van Zyl-Smit RN, 2020 [7]	Phase 3 double-blind, triple-dummy, clinical trial	High-dose Mometasone Furoate/ Indacaterol acetate (n=445) vs medium-dose Mometasone Furoate/ Indacaterol acetate (n=439) vs high-dose Mometasone Furoate (n=442) vs medium-dose Mometasone Furoate (n=444) vs high-dose Fluticasone/Salmeterol (n=446)	Asthma diagnosed for at least 1 year with an Asthma Control Questionnaire 7 (ACQ-7) score of at least 1·5	2216 (12-75 years)	Primary endpoint: FEV_1 at week 26<br><br><br><br><br><br><br><br><br><br><br><br><br><br>Secondary endpoint:<br>ACQ-7 score at week 26 from baseline.	Superiority of high-dose Mometasone Furoate/ Indacaterol acetate and medium-dose Mometasone Furoate/ Indacaterol acetate over corresponding mometasone furoate doses.<br><br>High-dose Mometasone Furoate/ Indacaterol acetate was non-inferior to high-dose Fluticasone/Salmeterol.<br><br>Significant improvement with combined doses of Mometasone Furoate/ Indacaterol versus combined doses of Mometasone Furoate.<br><br>Significant improvement of high-dose Mometasone Furoate/ Indacaterol acetate vs high dose Mometasone Furoate.<br><br>Significant improvement of medium-dose Mometasone Furoate/ Indacaterol acetate vs medium dose Mometasone Furoate.	Treatment difference [Δ] 132 mL [95% CI 88 to 176] ; p<0·001 and Δ 211 mL [167 to 255]; p<0·001), respectively<br><br>Δ 36 mL [−7 to 80]; p=0·101).<br><br>Δ −0·209 [95% CI −0·270 to −0·149]; p<0·001<br><br>OR (95% CI),1·51 (1·20 to 1·89); p<0·001<br><br>Δ −0·171 (95% CI, −0·257 to −0·086); p<0·001<br><br>OR (95% CI),1·31 (0·95 to 1·81);p=0·094<br><br>Δ −0·248 (95% CI, −0·334 to −0·162); p<0·001<br>95% CI),1·73 (1·26 to 2·37); p<0·001

						Significant improvement of high-dose Mometasone Furoate/ Indacaterol acetate once daily showed improvements vs high-Fluticasone/Salmeterol twice daily	Δ −0·054 [95% CI −0·140 to 0·031]; p=0·21<br><br>OR (95% CI),1·06 (0·76 to 1·46); p=0·75
					Mean evening PEF (L/min) during week 1-52	Significant improvement of medium-dose Mometasone Furoate/ Indacaterol acetate vs medium dose Mometasone Furoate	Treatment difference (95% CI),29·1 (23·3–34·8); p<0·001
						Significant improvement of high-dose Mometasone Furoate/ Indacaterol acetate vs high dose Mometasone Furoate.	Treatment difference (95% CI), 23·7 (18·0–29·5); p<0·001
						Significant improvement of high-dose Mometasone Furoate/ Indacaterol acetate once daily showed improvements vs high-Fluticasone/Salmeterol twice daily	Treatment difference (95% CI),9·1 (3·3–14·9); p=0·002
						Significant improvement of medium-dose Mometasone Furoate/ Indacaterol acetate vs medium dose Mometasone Furoate	Treatment difference (95% CI),30·2 (24·2–36·3); p<0·001
					Mean morning PEF (L/min) during week 1-52	Significant improvement of high-dose Mometasone Furoate/ Indacaterol acetate vs high dose Mometasone Furoate.	Treatment difference (95% CI), 28·7 (22·7–34·8); p<0·001

					Incidence of adverse events	Significant improvement of high-dose Mometasone Furoate/ Indacaterol acetate once daily showed improvements vs high-Fluticasone/Salmeterol twice daily<br><br>Similar across the treatment groups	Treatment difference (95% CI),13·8 (7·7–19·8); p<0·001
Comparison of the effect of fluticasone combined with salmeterol and fluticasone alone in the treatment of pediatric asthma.<br>Cao H (2021) [8]	Systematic Review and Metanalysis	Salmeterol/Fluticasone (n=4133) vs Salmeterol (n=4139)		8272	FEV_1<br><br>Asthma exacerbation<br><br>Incidence of adverse events	Significantly different<br><br>Significantly different (fixed effects model, RR=0.85, 95<br><br>No difference (P>0.05)	Fixed effects model, WMD=3.26, 95% CI: 1.52-5.00, P=0.0002<br><br>% CI: 0.73-0.98, Z=2.18, P=0.03).
Efficacy and safety of salmeterol/fluticasone compared with montelukast alone (or add-on therapy to fluticasone) in the treatment of bronchial asthma in children and adolescents: a systematic review and meta-analysis.<br>Zhou X (2021) [9]	Systematic Review and Metanalysis	salmeterol/fluticasone vs montelukast or combination of montelukast and fluticasone	Bronchial asthma	2643 (4-17 years)	Primary outcome: Risk of asthma exacerbation.<br><br>Secondary outcomes: Pulmonary function<br><br>Asthma control level<br><br>Quality of life	Metanalysis could not been performed (more studies favored salmeterol/fluticasone)<br><br>Salmeterol/fluticasone showed a significant improvement of peak expiratory flow %predicted.<br><br>Salmeterol/fluticasone showed a higher full-controlled level.<br><br>Salmeterol/fluticasone presented a higher childhood asthma control test score.<br><br>No analysis performed (one study favored Salmeterol/fluticasone)	MD: 5.45; 95% CI: 1.57–9.34; I2=95%; P=0.006<br><br>RR: 1.51; 95% CI: 1.24–1.85; I2=0; P<0.001<br><br>MD: 2.30; 95% CI: 1.39–3.21; I2=72%; P<0.001)

					Risk of hospitalization<br>Adverse events	No analysis performed (one study favored Salmeterol/fluticasone)<br><br>No analysis performed (no significant differences)	

MDPI = Multidose Dry Powder Inhaler; FEV_1= Forced Expiratory Volume in 1 s; LS= Least squares; PEF= peak expiratory flow; AUC= Area Under the Curve, MDI = Metered Dose Inhalers; BID= Bis In Die ACQ-7= Asthma Control Questionnaire 7; MD= Mean Difference.

References

1. Płoszczuk A, Bosheva M, Spooner K, McIver T, Dissanayake S. Efficacy and safety of fluticasone propionate/formoterol fumarate in pediatric asthma patients: a randomized controlled trial. Ther Adv Respir Dis. 2018 Jan-Dec;12:1753466618777924. doi: 10.1177/1753466618777924. PMID: 29857783; PMCID: PMC5985608.
2. Jorup C, Lythgoe D, Bisgaard H. Budesonide/formoterol maintenance and reliever therapy in adolescent patients with asthma. Eur Respir J. 2018 Jan 4;51(1):1701688. doi: 10.1183/13993003.01688-2017. PMID: 29301922.
3. Bateman ED, Reddel HK, O'Byrne PM, Barnes PJ, Zhong N, Keen C, Jorup C, Lamarca R, Siwek-Posluszna A, FitzGerald JM. As-Needed Budesonide-Formoterol versus Maintenance Budesonide in Mild Asthma. N Engl J Med. 2018 May 17;378(20):1877-1887. doi: 10.1056/NEJMoa1715275. PMID: 29768147.
4. Raphael G, Yiu G, Sakov A, Liu S, Caracta C. Randomized, double-blind trial evaluating the efficacy and safety of fluticasone propionate and fluticasone propionate/salmeterol delivered via multidose dry powder inhalers in patients with persistent asthma aged 12 years and older. J Asthma. 2018 Jun;55(6):640-650. doi: 10.1080/02770903.2017.1350971. Epub 2017 Aug 31. PMID: 28763243.
5. Zhang Y, Wang H. Efficacy of montelukast sodium chewable tablets combined with inhaled budesonide in treating pediatric asthma and its effect on inflammatory factors. Pharmazie. 2019 Nov 1;74(11):694-697. doi: 10.1691/ph.2019.9582. PMID: 31739840.

6. Weinstein CLJ, Gates D, Zhang X, Varnell T, Mok W, Vermeulen JH, Amar NJ, Jain N. A phase 3 study evaluating the safety and efficacy of a pediatric dose of mometasone furoate with and without formoterol for persistent asthma. Pediatr Pulmonol. 2020 Apr;55(4):882-889. doi: 10.1002/ppul.24667. Epub 2020 Feb 5. PMID: 32022483.
7. van Zyl-Smit RN, Krüll M, Gessner C, Gon Y, Noga O, Richard A, de Los Reyes A, Shu X, Pethe A, Tanase AM, D'Andrea P; PALLADIUM trial investigators. Once-daily mometasone plus indacaterol versus mometasone or twice-daily fluticasone plus salmeterol in patients with inadequately controlled asthma (PALLADIUM): a randomised, double-blind, triple-dummy, controlled phase 3 study. Lancet Respir Med. 2020 Oct;8(10):987-999. doi: 10.1016/S2213-2600(20)30178-8. Epub 2020 Jul 9. PMID: 32653075.
8. Cao H, Gu J, Dai J, Yang G, Tang H, Ding A. Comparison of the effect of fluticasone combined with salmeterol and fluticasone alone in the treatment of pediatric asthma. Minerva Pediatr (Torino). 2021 Oct;73(5):452-459. doi: 10.23736/S2724-5276.21.05939-5. Epub 2021 May 14. PMID: 33988019.
9. Zhou XJ, Qin Z, Lu J, Hong JG. Efficacy and safety of salmeterol/fluticasone compared with montelukast alone (or add-on therapy to fluticasone) in the treatment of bronchial asthma in children and adolescents: a systematic review and meta-analysis. Chin Med J (Engl). 2021 Nov 15;134(24):2954-2961. doi: 10.1097/CM9.0000000000001853. PMID: 34784306; PMCID: PMC8710318.

PICO question 4. In children with uncontrolled asthma symptoms despite daily therapy, what is the preferred option between increasing the therapy or assess modifiable factors (adherence, inhalation technique, exposure to allergens)?

Patient or population: children and adolescents with asthma

Setting: tertiary care

Intervention: increasing therapy

Comparison: assessing modifiable factors

Outcome: symptom control

Search strategy:

"Asthma"[Mesh] OR "asthma" AND ("Anti-Asthmatic Agents"[Mesh] OR "Adrenergic beta-2 Receptor Agonists"[Mesh] OR "Adrenal Cortex Hormones"[Mesh] OR "inhaled steroid*" OR "adherence" OR "medication adherence"[Mesh] OR "inhalation" OR "treatment outcome"[Mesh])

Filters applied: last 5 years, Child: 6-12 years, Adolescent: 13-18 years

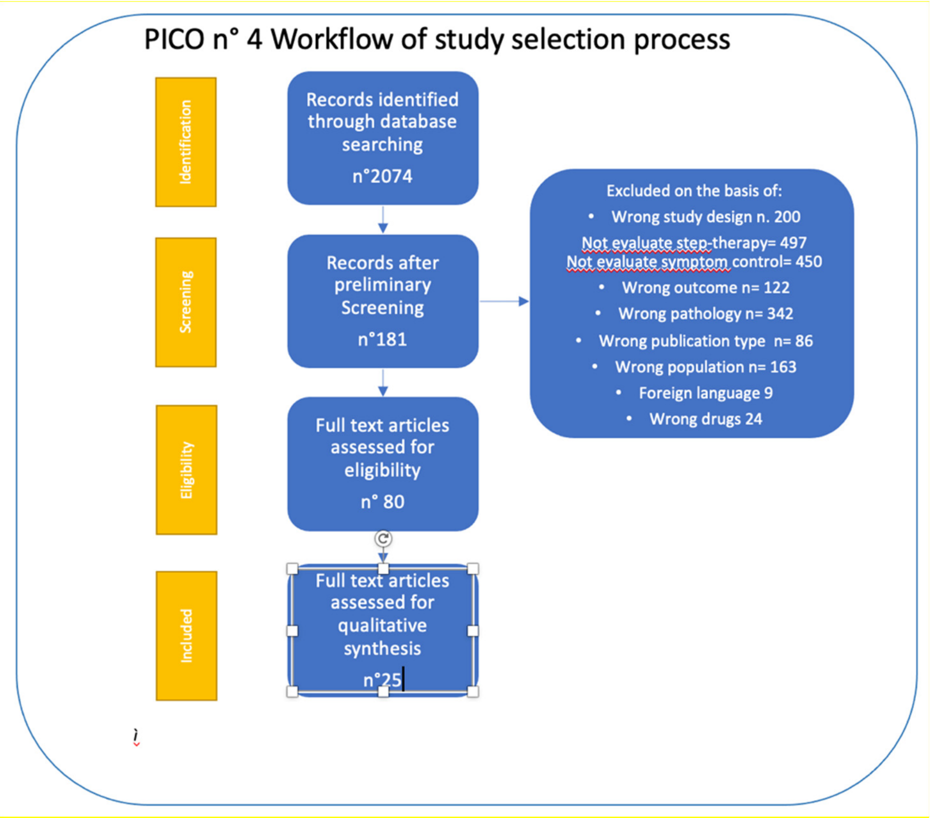
PICO n° 4 Workflow of study selection process
Identification
Screening
Eligibility
Included
Records identified through database searching
n°2074
Records after preliminary Screening
n°181
Full text articles assessed for eligibility
n° 80
Full text articles assessed for qualitative synthesis
n°25
Excluded on the basis of:
Wrong study design n. 200
Not evaluate step-therapy= 497
Not evaluate symptom control= 450
Wrong outcome n= 122
Wrong pathology n= 342
Wrong publication type n= 86
Wrong population n= 163
Foreign language 9
Wrong drugs 24

Title of the study, first author, year	Type of the study	Study design	Population	N° of patients age	Methods	Outcomes	
Treatment of childhood asthma with anti-IgE antibody (omalizumab). Milgrom, H et al. 2001 [1]	Randomised Control Trial (RCT)	Omalizumab + ICS (Budesonide Dry Powder, BDS) vs Placebo + ICS (BDP)<br><br>28 weeks	Children with moderate-severe asthma	334; 6-12 yrs	Asthma exacerbations<br><br>Global Evaluation of Treatment Effectiveness (GETE)	Omazlizumab was effective in reducing the risk of acute exacerbations in children with moderate-severe asthma<br><br>Physician assessed GETE was higher in patients treated with Omalizumab	RR=0,62 (0,40-0,97)<br><br>RR=1,65 (1,29-2,11)
Adherence feedback to improve asthma outcomes among inner-city children: a randomised trial. Otsuki, M et al. 2009. [2]	RCT	EMD (Doser CTTM + MEMS® Caps) vs Usual care (Booklet)	Children with 2 visit in ER or 1 hospitalisation	166; 2–12 yrs	Inhaler adherence (Rate)	EMD did not improve medication adherence	0,04 (-0,52/0,59) p=0,89
Providing feedback on adherence increases use of preventive medication by asthmatic children, Burgess. SW, et al. 2010 [3]	RCT	Electronic Monitoring Device (EMD, SmartInhalerTM) vs Placebo	Children with not well controlled asthma (FEV1 <80%)	26; 6–14 yrs	Inhaler adherence (Rate)<br><br>Asthma exacerbation (Rate)	EMD did not improve adherence nor exacerbations rate	0,51 (-0,40/1,43) p=0,27<br><br>0,59 (-0,70/1,87) p=0,37
Randomised trial of omalizumab (anti-IgE) for asthma in inner-city children. Busse, WW et al. 2011 [4]	RCT	Omalizumab + guideline-based treatment vs Placebo + guideline-based treatment<br>60 weeks	Children with moderate-severe asthma	419; 6-20 yrs	Asthma exacerbations	Omalizumab was effective in reducing the risk of acute exacerbations in children with moderate-severe asthma	RR=0,46 (0,38-0,55)
Mobile-based	single arm,	Patients were provided	Adolescents	20; 12-17	Mobile application	Adolescents utilised the	

asthma action plans for adolescents.<br>Burbank, AJ et al. 2015 [5]	feasibility and proof-of-concept study	with a mobile app giving management instructions depending on their symptoms or PEF<br>8 weeks	with persistent asthma	yrs	usage rates<br><br>Patient satisfaction<br><br>Pre- and post-intervention ACT (asthma control test) score<br><br>Child asthma self-efficacy scores	mobile app a median 4.3 days/week to record peak flow rates and/or asthma symptoms<br><br>93% of the population thought they were better able to control their asthma by utilising the mobile app<br><br>No ACT score improvement in the overall population; ACT improvement in subgroup with uncontrolled asthma<br><br>No improvement in total self-efficacy score; improvement in the asthma attack prevention domain	Median (IQR): 20 (16–23) pre-intervention and 21.5 (16– 23) post-intervention (p = 0.53).<br>subgroup with uncontrolled asthma: from 16 (13–17) at baseline to 18 (16–23) post-intervention (p = 0.03)<br><br>Median (IQR) :60.5 (54–64) pre-intervention and 62 (56–64) post-intervention (p = 0.13)<br>-asthma attack prevention domain: from 34 (33–36) to 36 (33–38; p = 0.04). Among adolescents with uncontrolled asthma at baseline, median scores for the asthma attack

							prevention domain were 33 (32–36) at baseline and 35 (33–36) post-intervention (p = 0.36)
A tailored mobile health intervention to improve adherence and asthma control in minority adolescents. Mosnaim, G et al. 2015 [6]	treatment group only proof-of-concept study	Effects of the M-ADEPT app, that provides therapy reminders Basketball game and Positive text messages for each puff of ICS taken (positive reinforcement)<br>8 weeks	African American adolescents with persistent asthma	12; 11-16 yrs	ICS adherence (≥50%)<br><br>ACT score median<br><br>Reliever use	The M-ADEPT app was effective in increasing adherence and ACT scores and lowering SABA use<br><br>18 at baseline to 23 at week 8. 58% of participants achieved the minimal clinically important difference (3 points) in ACT score from baseline to week 8.<br><br>Short acting beta-agonists (SABA) use decreased from a median of 3 puffs per week at baseline to 0 puffs per week at 8 weeks.	8% and 58% of participants met target at baseline and week 8
The effect of an electronic monitoring device with audiovisual reminder function on adherence to inhaled corticosteroids and school attendance in children with asthma: a randomised controlled trial.	RCT	The study analysed the effect of audiovisual reminders (AVR) on treatment adherence vs standard care<br>6 months	Children and adolescents with asthma	220; 6-15 yrs	ICS adherence	AVR significatively improved adherence to ICS therapy	78% (SD 19%) vs 35 (23); 2,03 (1,70-2,36); p <0,0001

Chan, AH et al. 2015 [7]							
e-monitoring of asthma therapy to improve compliance in children (e-MATIC): a randomised controlled trial. Vasbinder, EC et al. 2016.[8]	RCT	EMD (RTMM device, eMDITM) vs Placebo	Children with asthma ≥ 6 months	219; 4–11 yrs	Inhaler adherence (Rate)<br><br>Asthma control (C-ACT score)<br><br>Asthma exacerbation per year	EMD significantly improved medication adherence<br><br>EMD did not significatively modify C-ACT scores or asthma exacerbation rates	intervention group: 69.3% (95% CI 65.5–73.4%) vs 57.3% (95% CI 52.8–61.7%) control group.<br><br>21.10 vs 22.17 −1.07 (−3.51–0.56) 0.203<br><br>0.23 vs 0.37 −0.14 (−0.61–0.25) 0.432
A phase III randomised controlled trial of tiotropium add-on therapy in children with severe symptomatic asthma. Szefler SJ et al. 2017 [9]	RCT	Tiotropium, 5; Tiotropium, 2.5; or Placebo<br>12 weeks	Children with severe asthma	401; 6-11 yrs	FEV_1<br><br>Asthma control[11]	Tiotropium add on therapy was effective in improving FEV1<br><br>Tiotropium add on therapy was not effective in improving quality of life	0.19 (0.43) vs 0.14 (0.31); standardised mean difference 0.05 (–0.02 to 0.13)[12]<br><br>–0.95 (0.73) vs –0.97 (0.54); standardised mean difference 0.04 (–0.17 to 0.25)[12]

					Asthma exacerbations	Tiotropium add on therapy was effective in reducing the risk of acute exacerbations	10/266 vs 8/134; 0.63 (0.25-1.56)[12]
Electronic adherence monitoring device performance and patient acceptability: a randomised control trial. Chan, AHY et al. 2017 [10]	RCT	EMD (SmartTrackerTM) vs Placebo	Children with asthma exacerbation	220; 6–15 with	Inhaler adherence (Rate)<br><br>Asthma control (C-ACT score)<br><br>Asthma exacerbation (Rate)<br><br>FEV1(% predicted)<br><br>Acceptability (Scores)	EMD significantly improved medication adherence and asthma control, but failed to improve exacerbation rate and lung's function	1,27 (0,93/1,61) p=0,00<br><br>0,33 (0,06/0,59) p=0,02<br><br>-0,03 (-0,43/0,36) p=0,90<br><br>0,23 (-0,04/0,49) p=0,09
Electronic monitoring of adherence to inhaled corticosteroids: an essential tool in identifying severe asthma in children. Jochman A et al. 2017 [11]	prospective observational cohort study	Adherence was assessed via EMD in order to determine whether it had an impact on asthma control.<br><br>Median (range) duration of monitoring was 92 (56–200) days.	Asthmatic children already prescribed inhaled corticosteroids	93; 5-17 yrs	FEV_1<br><br>Bronchodilator reversibility	Median respiratory function (FEV1 and bronchodilator reversibility) improved significatively in good and medium adherence group but not in low adherence group	92 vs 96 p<0.0001 (good adherence group), 85 vs 91 p=0.02 (medium adherence group), 88 vs 91 p=0.14 (low adherence group)<br><br>7 vs 4,1 p=0.0080 (good

							adherence group), 9,1 vs 3,7 p=0.0043 (medium adherence group), 8,1 vs 4,7 p=0.1919 (low adherence group)
					FeNO	FeNO decreased significantly in good and medium adherence group	23 vs 13 p<0.0266 (good adherence group), 35 vs 19 p=0.0016 (medium adherence group), 41 vs 45 p=0.9772 (low adherence group)
					mPAQLQ	Mini Asthma quality of life questionnaire scores improved significantly in good and medium adherence group	5,2 vs 6,2 p<0.0001 (good adherence group), 3,9 vs 5,5 p= 0.004 (medium adherence group), 5,1 vs 5,6 p= 0.13 (low adherence group)
					Exarbations	Exacerbations were significantly less frequent in children with good-medium adherence	1 vs 0 p<0.001 (good adherence group), 1 vs 0 p=0.001 (medium

							adherence group), 0 vs 1 p= 0.10 (low adherence group)
STAAR: a randomised controlled trial of electronic adherence monitoring with reminder alarms and feedback to improve clinical outcomes for children with asthma. Morton, RW et al, 2017 [12]	RCT	EMD (SmartTrackerTM and SmartTurbosTM,) vs Placebo	Children with poorly asthma control (ACQ ≥ 1.5)	90; 6–16 yrs	Inhaler adherence (Rate)<br><br>Asthma control (ACQ Score)<br><br>FEV_1 (% predicted)	EMD significantly improved medication adherence, but failed to improve exacerbation rate and lung's function	0,79 (0,27 /1,30) p=0,00<br><br>0,02 (-0,40/0,43) p=0,94<br><br>0,18 (-0,23/0,59) p=0,39
Adherence to asthma treatment and their association with asthma control in children. Basharat, S et al., 2018 [13]	Descriptive cross-sectional study	Healthcare professionals assessed asthma control according to GINA guidelines and adherence to therapy via the MMAS[3] questionnaire	Persistent asthma patients who were taking medication for at least 1 year	310; 4-15 yrs	Adherence to controller therapy	Children with higher adherence to controller therapy had a higher chance of having well controlled asthma	Children with well controlled asthma had a significantly higher adherence to therapy (29.70% of the high adherence group; 14.08% of the medium adherence and 18.8% of the poor adherence) (p=0.031)

Severe asthma in paediatrics: Outcomes of the implementation of a special health care protocol. Giubergia, V et al. 2018 [14]	Cross-sectional, observational, and analytical study	The treating team systematically assessed the inhalation technique and worked on treatment adherence, environmental control, and the patient's housing conditions.<br>6 months (1 visit per month)	Children with uncontrolled symptoms with high-dose ICS (Patients were assessed by an interdisciplinary team according to the WHO protocol to differentiate those with severe treatment-resistant asthma (STRA) from those with severe difficult-to-treat asthma (SDCA)	69; 6-18 yrs (48% SDCA, 52% STRA)	No treatment adherence[7]	Treatment adherence was the only factor significantly different between SDCA and STRA	86% vs 56% p=0.01
					Initial/final FVC	In both groups, FVC, FEV_1 and FEF 25/75% improved significantly after follow up, but the increase was higher in the STRA group.	94.7% (89.5-100)/ 98.8% (93-103) (p=0,04) vs 99% (93-105)/ 105.7% (101-109.7) (p=0,01)
					Initial/final FEV_1	FEV_1/ FVC increased significantly only in the STRA group	89.7% (89-95)/ 93.9% (89-98) (p=0,04) vs 87% (80-94)/ 98.4% (93-104)(p=0,0001)
					Initial/final FEV_1/ FVC		85.5 (82-88)/ 84 (81-86) (p=0,2) vs 79 (75-83)/ 83 (80-86) (p=0,003)
					Initial/final FEF 25/75%		81.8% (72-91)/ 88.4% (76-99) (p=0,05) vs 74.5% (60-88)/ 85.7% (75-96) (p=0,02)

A Prospective, Randomised, Controlled Study of Inhaler Electronic Monitoring Devices to Improve Adherence in Children with Asthma. Simoneau, T et al. 2019 [15].	RCT	EMD (BreathSmart® app + HeroTracker® Sensor) vs Placebo	Children with various asthma control	43; 8–17 yrs	Inhaler adherence (Rate)	EMD did not improve medication adherence	0,50 (-0,54/1,54) p=0,36
Treatment adherence and level of control in moderate persistent asthma in children and adolescents treated with fluticasone and salmeterol. Jentzsch NS et al. 2019 [16]	prospective observational study	Asthma control levels and adherence to treatment were analysed. 6 months	Children with moderate persistent asthma	84; 5-16 yrs	Adherence	Adherence was significantly higher in children with good asthma control as compared with children with bad asthma control	2 months: 71.7 (13.2) vs 87.8 (8.8) p<0,0001<br><br>4 months: 56.0 (8.7) vs 74.9 (11) p<0,0001<br><br>6 months: 47.6 (11.1) vs 62.1 (13.5) p=0,002
Influence of weight status in the response to Step-2 maintenance therapies in children with asthma. Longo, C et al. 2019 [17]	Historical cohort study	The effects of weight status (BMI percentile) on time-to-management failure were estimated	Children with an asthma diagnosis confirmed by a specialist, on low-dose ICS or leukotriene antagonists (LTRA) (Step-2) maintenance monotherapies	518 visits from 342 patients, 2–18 yrs	time-to-management failure[13]	Higher BMI was associated with higher risk of management failure	Overall HR for every 10 BMI points increase: 1.09 (1.03 to 1.16)

A randomised controlled trial of a mobile application-assisted nurse-led model used to improve treatment outcomes in children with –asthma. Lv S et al, 2019 [18]	RCT	mobile application medication reminder, adherence management, alert of acute asthma exacerbations, assessment of ex- acerbation severity, treatment recommendation, keeping a health diary, instant communication with healthcare providers and health education vs standard care	Children with asthma	152; 6-12 yrs	Asthma exacerbations[14]<br><br>Treatment adherence[15]<br><br>C-ACT scores[15]<br><br>Respiratory infections (times/year)[15]<br><br>Antibiotic use (days/year)[15]<br><br>Days of school absence[15]<br><br>Days of parental work loss[15]	The mobile app used in the study helped increasing treatment-adherence and C-ACT scores as well as lowering exacerbations, infections, antibiotic use, school and workdays loss and medical expense, with better results than standard care	experimental group: 9 (7–10) vs 3 (2–4), p < 0.001; control group 9 (7–11) vs 4 (3–5), p < 0.001; inter-group difference p<0,05<br><br>94.46 vs 92.67, inter-group difference p<0,05<br><br>24.36 vs 22.44, inter-group difference p<0,05<br><br>3 (IQR 2-4) vs 4 (IQR 3-5), inter-group difference p<0,05<br><br>9.14 vs 10.51, inter-group difference p<0,05<br><br>1,25 (IQR 1-1,5) vs 2 (IQR 1,5-3) inter-group difference p<0,05

					Medical expenses (RMB Yuan/year))15		3 (IQR 2,-4) vs 4 (IQR 3,4-4,5), inter-group difference p<0,05<br><br>931 vs 1179, inter-group difference p<0,05
Treatment of allergic rhinitis reduces acute asthma exacerbation risk among asthmatic children aged 2–18 years; Yu, CL et al. 2019 [19]	Cohort study	Allergic Rhinitis had an impact on acute exacerbation (AE) and whether intranasal corticosteroid (INCS) and second-generation antihistamines (SGH) for AR modified the association of AR with AE in asthmatics aged 2-6 years and 7-18 years	Patients witch diagnosis of asthma in the years 2000 through 2012	2-18 yrs	Cox proportional hazards regression analysis	The appropriate diagnosis of AR is important so that AR can be properly controlled to reduce the worsening of asthma	The incidence of AE was higher in the preschool group than the older group (HR: 1.68, 95% CI: 1.44-1.95).<br><br>The use of INCS and/or SGH was associated with a significant reduction in the occurrence of AE among AR patients aged 2-6 years old (HR: 0.38, 0.57 and 0.45) and 7-18 years old (HR: 0.50, 0.52 and 0.35)

Ambulatory Management of Childhood. Asthma Using a Novel Self-management Application. Nkoy FL, 2019 [20]	Prospective Cohort	Matched Controls	Children 2 to 17 years with persistent who received asthma care in the previous year at participating clinics	327; 2-17 yrs	Longitudinal changes for the child and parents<br><br>Comparing ED and hospital admissions and oral corticosteroid use pre- and post intervention,<br><br>Comparing ED and hospital admissions and OCS use between e-AT users and matched controls	e-AT use led to high and sustained participation in self-monitoring and improved asthma outcomes	reduced ED and hospital admissions (rate ratio [RR]: 0.68; 95% confidence interval [CI]: 0.49–0.95) and OCS use (RR: 0.74; 95% CI: 0.61–0.91).<br><br>Compared with matched controls, participants had reduced ED and hospital admissions (RR: 0.41; 95% CI: 0.22–0.75) and OCS use (RR: 0.65; 95% CI: 0.46–0.93).
Asthma-related outcomes associated with indoor air pollutants among schoolchildren from four informal settlements in two municipalities in the Western Cape Province of South Africa. Olaniyan, T et. al. 2019 [21]	Cross-sectional study	The association between asthma and common indoor exposures.	Children to 9-11 years of. age	590; 9-11 yrs	ISAAC questionnaire for the caregivers and evaluation of pulmonary function<br><br>Logistic regression models	Visible mould growth, paraffin use for cooking, and passive smoking were associated with a twofold to threefold increased rock in upper and lower airway outcomes	Association with mould growth (aOR 3,37, 95% CI: 1,69-6,71)

The effect of electronic monitoring combined with weekly feedback and reminders on adherence to inhaled corticosteroids in infants and yrsunger children with asthma: a randomised controlled trial. Chen, J et al. 2020 [22]	RCT	EMD (SmartTrackerTM) vs Placebo	Children with regular ICS	116; 6 mo – 3 yrs	Inhaler adherence (Rate)	EMD significantly improved medication adherence	0,86 (0,32/1,38) p=0,00
Nonadherence to inhaled corticosteroids: A characteristic of the paediatric obese-asthma phenotype?. Orriens et al 2021 [23]	Cross-sectional Study	Influence of excess weight on adherence to corticosteroid therapy in asthma.	Children with asthma	566; 4-13 years of age		Excess weight was associated with. General nonadherence to ICS, but only in children with moderate-to-severe asthma	Excess weight was associated with a nonadherent behaviour (OR: 1,54 95% CI; 0,84-2.81) in severe asthma<br><br>This association appeared to be stronger in younger (OR 2,17; 95% CI 1.00-4,73) Vs older children
Psychosocial factors and lack of asthma knowledge undermine child and adolescent		Psychiatric questionnaire and evaluation adherence at the first time and after asthma education. Follow-up (3-6 month)	Children with asthma	134; 8-18 years of age	Evaluation of ICS adherence Depression and anxiety were assessed using	Psychological evaluation is warranted in paediatric patients with asthma<br><br>The mean ICS adherence	

adherence to inhaled corticosteroid. Takkinsatian et al 2022 [24]					psychiatric questionnaires.	was 75.9 ± 27.5%. 57patients (42.5%) were defined as having poor adherence (<75%)<br><br>Patients with ICS adherence <75% had unfavourable expectations from asthma treatment and a higher proportion of inhaled short-acting beta-agonist use before exercise<br><br>Depression and anxiety 27.5% and 23.3%, respectively<br><br>Significant improvement in ICS adherence (p = 0.02) and Asthma Control Test scores (p = 0.02) were observed at the follow-up visit	[OR]: 1.05, 95% [CI]: 1.01–1.10), (OR: 4.12, 95% CI: 1.27–13.36).<br><br>p > 0.05.
Assessment of level of asthma control and related factors in children attending pediatric respiratory clinics in Addis Ababa, Ethiopia. Aschalew , A et al. 2022 [25]	cross-sectional study	Healthcare professionals assessed asthma control via questionnaires[2] and the related risk factors for poor control	Children with physician-diagnosed asthma and on controller therapy for a minimum of 3 months	105; 1-14 yrs	Adherence to controller therapy<br><br>Poor inhaler technique	Adherence to controller therapy and inhalation technique were both significative factors in determining the level of asthma control in children	Children with poor adherence had a higher risk of uncontrolled asthma (defined based on a total C-ACT or ACT score≤19 or TRACK score≤80) (aOR=3.23; 95% CI 1.2–10.2; P=0.045)<br><br>Children with

							poor inhaler had a higher risk of uncontrolled asthma (aOR =3.48; 95% CI 1.19–10.3; P=0.024)

KIDMED: Mediterranean diet quality index, MDS: Mediterranean Diet Score, aβ: adjusted β coefficient, aOR: adjusted odds ratio.

[1] High scores in KIDMED were associated with lower risk of both inhalant and food allergens sensitization; high MDS was only associated with lower risk of inhalant sensitization.

[2]Test for Respiratory and Asthma Control in Kids (TRACK) for <5yrs, Childhood Asthma control test (C-ACT) for 5–<12 years, Asthma Control Test (ACT) for ≥12 years

[3]Morisky medication adherence assessment

[4]Severe persistent asthma was diagnosed if patients fulfilled one major and at least two minor criteria as recommended by the Severe Asthma Research Program (SARP) guideline

[5]Results are given as comparison between patients with and without fungal sensitization; respectively.

[6]Results are given as comparison between children with higher vs lower adherence to TMD

[7]Results are given as comparison between children with SDCA vs STRA

[8]LP: Lactobacillus paracasei, LF: Lactobacillus fermentum

[9]Results are given as LP vs LF vs LP+LF as compared to placebo

[10]Results are given as mean adherence rates in uncontrolled asthma vs controlled asthma

[11]Assessed via the Asthma Control Questionnaire 7

[12]Results are given as pooled triple therapy (with tiotropium) vs dual therapy (ICS and LABA without tiotropium)

[13]Defined as any step-up in therapy, acute care visit, hospitalisation or oral corticosteroids for asthma

[14]Results are given as pre-intervention vs post-intervention and then compared between experimental vs standard care group

[15]Results are given as experimental vs standard care group

References

1. Milgrom H, Berger W, Nayak A, Gupta N, Pollard S, McAlary M, et al. Treatment of childhood asthma with anti-immunoglobulin E antibody (omalizumab). Pediatrics. 2001;108: E36.
2. Otsuki M, Eakin MN, Rand CS, Butz AM, Van Doren H, Zuckerman IH, et al. Adherence Feedback to Improve Asthma Outcomes Among Inner-City Children: A Randomized Trial. Pediatrics. 2009. pp. 1513–1521. doi:10.1542/peds.2008-2961
3. Burgess SW, Sly PD, Devadason SG. Providing feedback on adherence increases use of preventive medication by asthmatic children. J Asthma. 2010;47: 198–201.
4. Busse WW, Morgan WJ, Gergen PJ, Mitchell HE, Gern JE, Liu AH, et al. Randomized trial of omalizumab (anti-IgE) for asthma in inner-city children. N Engl J Med. 2011;364: 1005–1015.
5. Burbank AJ, Lewis SD, Hewes M, Schellhase DE, Rettiganti M, Hall-Barrow J, et al. Mobile-based asthma action plans for adolescents. J Asthma. 2015;52: 583–586.
6. Mosnaim G, Li H, Martin M, Richardson D, Belice PJ, Avery E, et al. A tailored mobile health intervention to improve adherence and asthma control in minority adolescents. J Allergy Clin Immunol Pract. 2015;3: 288–290.e1.
7. Chan AHY, Stewart AW, Harrison J, Camargo CA, Black PN, Mitchell EA. The effect of an electronic monitoring device with audiovisual reminder function on adherence to inhaled corticosteroids and school attendance in children with asthma: a randomised controlled trial. The Lancet Respiratory Medicine. 2015. pp. 210–219. doi:10.1016/s2213-2600(15)00008-9
8. Vasbinder EC, Goossens LMA, Rutten-van Mölken MPMH, de Winter BCM, van Dijk L, Vulto AG, et al. e-Monitoring of Asthma Therapy to Improve Compliance in children (e-MATIC): a randomised controlled trial. Eur Respir J. 2016;48: 758–767.

9. Szefler SJ, Murphy K, Harper T 3rd, Boner A, Laki I, Engel M, et al. A phase III randomized controlled trial of tiotropium add-on therapy in children with severe symptomatic asthma. J Allergy Clin Immunol. 2017;140: 1277–1287.
10. Chan AHY, Stewart AW, Harrison J, Black PN, Mitchell EA, Foster JM. Electronic adherence monitoring device performance and patient acceptability: a randomized control trial. Expert Rev Med Devices. 2017;14: 401–411.
11. Jochmann A, Artusio L, Jamalzadeh A, Nagakumar P, Delgado-Eckert E, Saglani S, et al. Electronic monitoring of adherence to inhaled corticosteroids: an essential tool in identifying severe asthma in children. Eur Respir J. 2017;50. doi:10.1183/13993003.00910-2017
12. Morton RW, Elphick HE, Rigby AS, Daw WJ, King DA, Smith LJ, et al. STAAR: a randomised controlled trial of electronic adherence monitoring with reminder alarms and feedback to improve clinical outcomes for children with asthma. Thorax. 2017;72: 347–354.
13. Basharat S, Jabeen U, Zeeshan F, Bano I, Bari A, Rathore AW. Adherence to asthma treatment and their association with asthma control in children. J Pak Med Assoc. 2018;68: 725–728.
14. Giubergia V, Ramirez Farías MJ, Pérez V, González A, Crespi N, Fridman N, et al. Severe asthma in pediatrics: Outcomes of the implementation of a special health care protocol. Arch Argent Pediatr. 2018;116: 105–111.
15. Simoneau T, Sun Y, Gherlone N, Almeida S, Manice M, Hollenbach JP. A Prospective, Randomized, Controlled Study of Inhaler Electronic Monitoring Devices to Improve Adherence in Children with Asthma. D105 IMPROVING PEDIATRIC ASTHMA CONTROL AND OUTCOMES. American Thoracic Society; 2019. pp. A7177–A7177.
16. Jentzsch NS, Silva GCG, Mendes GMS, Brand PLP, Camargos P. Treatment adherence and level of control in moderate persistent asthma in children and adolescents treated with fluticasone and salmeterol. J Pediatr . 2019;95: 69–75.
17. Longo C, Bartlett G, Schuster T, Ducharme FM, MacGibbon B, Barnett TA. Influence of weight status in the response to Step-2 maintenance therapies in children with asthma. BMJ Open Respir Res. 2019;6: e000401.
18. Lv S, Ye X, Wang Z, Xia W, Qi Y, Wang W, et al. A randomized controlled trial of a mobile application-assisted nurse-led model used to improve treatment outcomes in children with asthma. J Adv Nurs. 2019;75: 3058–3067.
19. Yu C-L, Huang W-T, Wang C-M. Treatment of allergic rhinitis reduces acute asthma exacerbation risk among asthmatic children aged 2-18 years.

J Microbiol Immunol Infect. 2019;52: 991–999.

20. Nkoy FL, Fassl BA, Wilkins VL, Johnson J, Unsicker EH, Koopmeiners KJ, et al. Ambulatory Management of Childhood Asthma Using a Novel Self-management Application. Pediatrics. 2019. doi:10.1542/peds.2018-1711
21. Olaniyan T, Dalvie MA, Röösli M, Naidoo R, Künzli N, de Hoogh K, et al. Asthma-related outcomes associated with indoor air pollutants among schoolchildren from four informal settlements in two municipalities in the Western Cape Province of South Africa. Indoor Air. 2019;29: 89–100.
22. Chen J, Xu J, Zhao L, Zhang J, Yin Y, Zhang F. The effect of electronic monitoring combined with weekly feedback and reminders on adherence to inhaled corticosteroids in infants and younger children with asthma: a randomized controlled trial. Allergy Asthma Clin Immunol. 2020;16: 68.
23. Orriëns LB, Vijverberg SJH, Maitland-van der Zee AH, Longo C. Nonadherence to inhaled corticosteroids: A characteristic of the pediatric obese-asthma phenotype? Pediatr Pulmonol. 2021;56: 948–956.
24. Takkinsatian Md P, Chantaratin Md S, Sirisakpanit MSc Clinical Psychology S, Srisuwatchari Md W, Pacharn Md P, Jirapongsananuruk Md O, et al. Psychosocial factors and lack of asthma knowledge undermine child and adolescent adherence to inhaled corticosteroid. J Asthma. 2022;59: 2234–2245.
25. Aschalew A, Kebed RA, Demie TG, Weldetsadik AY. Assessment of level of asthma control and related factors in children attending pediatric respiratory clinics in Addis Ababa, Ethiopia. BMC Pulm Med. 2022;22: 70.

PICO question 5. In children with asthma, is metered dose inhaler (MDI) preferred to dry powder inhalers (DPI)?

Patient or population: children and adolescents with asthma

Setting: primary to tertiary care

Intervention: MDI

Comparison: DPI

Outcome: patient's preference

Search strategy:

"Asthma"[Mesh] OR "asthma" AND ("Anti-Asthmatic Agents"[Mesh] OR "inhalation" OR "administration, inhalation"[Mesh] OR "treatment outcome"[Mesh] OR "nebulizers and vaporizers"[Mesh] OR "metered dose inhaler" OR "MDI" OR "dry powder inhalers" OR "DPI")

Filters applied: last 5 years, Child: 6-12 years, Adolescent: 13-18 years

PICO n° 5 - Workflow of study selection process

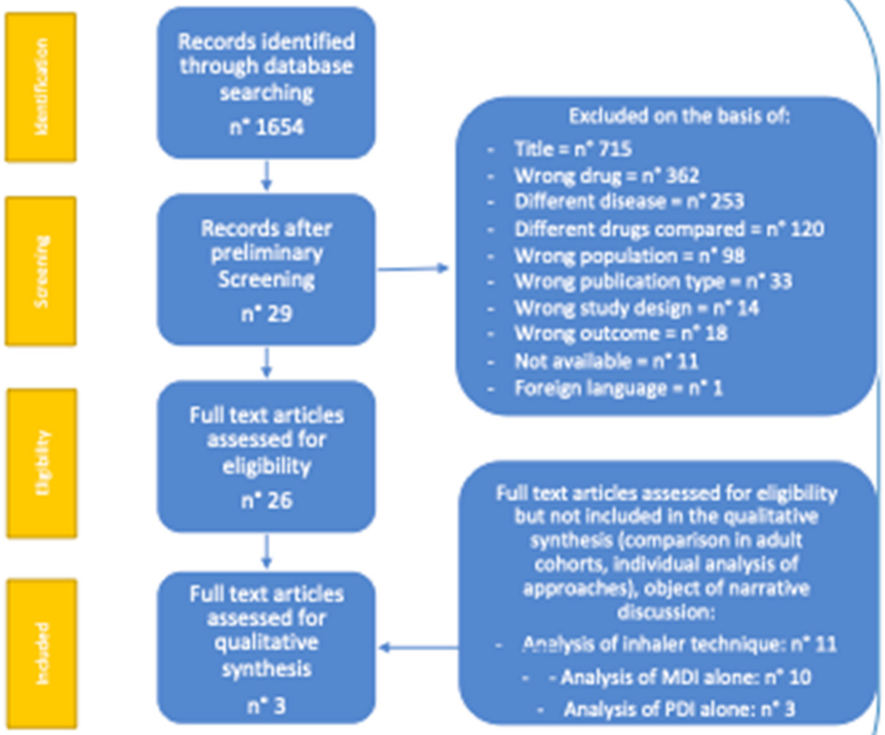

Modified from: Moher D, Liberati A, Tetzlaff J, Altman DG, The PRISMA Group (2009). Preferred Reporting Items for Systematic Reviews and Meta-Analyses: The PRISMA Statement. PLoS Med 6(7): e1000097

Title of the study, first author, year	Type of study	Study design	Population	N of patients (age)	Methods	Outcomes	
Impact of payor-initiated switching of inhaled corticosteroids on lung function. Bickel S, 2021 [1]	Retrospective chart review	Evaluate the impact of switching ICS from MDI to PDI (n= 24) on lung function (FEV1, FEF25-75) compared with children who remained on MDI (n=27)	Asthmatic children	111 (6-18 years)	Lung function at visit 1 and visit 2 (6 months)<br>- switched to DPI<br>- Remained on MDI	The change to a different inhaler device had a detrimental impact on lung function	FEV_1 declines from 98,5% to 91% (p=0.013)<br>FEF25%-75% from 89,5% to 76% (p=.041)<br><br>No statistically significant change in FEV_1 or FEF25-75
Predictors of proper inhaler technique and asthma control in pediatric patients with asthma. Alomani BA, 2020 [2]	A cross-sectional non-interventional study	To evaluate the proper inhaler techniques and factors affecting the application of inhaler technique among pediatric patients with asthma who self-administer their devices	Patients with an asthma diagnosis for ≥6months, who self-administer their controller steroid inhaler devices	150 (7–17 years)	- Appropriation of inhaler use: check-list step-to-step for MDI (89.4% of patients) and check-list step-to-step for DPI (Turbohaler 34.7%, Diskus 25.3%, and Handihaler 5.3% of patients). Appropriate technique if no error performed<br><br>-Level of asthma control: Asthma Control Test (ACT) in >12 years old patients; Childhood Asthma Control Test (C-ACT) in 4-11 years old patients.<br><br>-Parental knowledge: Athma Knowledge Questionnaire (AKQ)<br>-Children's stigma: Child	The majority of patients used MDI inappropriately compared to DPIs. Proper patients and/or parents' education on how to use inhaled devices is fundamental to achieve adequate asthma control. Clinical pharmacists would play a major role in reinforcing inhaler technique use.<br><br>Parental level of education and pediatrics' stigma independently associated with asthma control.<br><br>Influences number of correct MDI steps and of errors in critical steps.	MDI: 13.4%<br>Turbohaler: 38.5%<br>Diskus: 28.9<br>Handihaler: 12.5%<br><br>Parental education: OR = 5.181; 95% CI = 1.238–21.677; p = .024<br><br>Stigma score: OR = 2.825; 95% CI = 1.420–5.619; p = .003<br><br>OR=1.066; 95% CI=1.010–1.125; p= .020

					Attitude Toward Illness Scale (CATIS)<br>-Medications' adherence: medication adherent scale		
Assessment of regular drug use and inhaler technique skills in asthmatic children. Can C, 2019 [3]	Prospective observational study	Information on medication and demonstration on inhalation technique was given and one month later patients demonstrate their inhaler techniques (MDI, Turbuhaler, capsule-based DPI)	Asthmatic children with long term asthma control medication	100; (6-18 years)	All correct steps of the inhalation techniques<br><br>Most common mistakes<br><br>Adherence to drugs:<br>- Age<br>- Gender<br>- Level of asthma control<br>- Drugs by mouth or inhalation<br>- Number of drugs	Repeated training is necessary to ensure asthma control and successful treatment<br><br>The most common cause of irregular drug use is forgetting to take the drug<br><br>The age is the most important factor affecting regular use of medications: it was better in younger childrens	60.6 % MDI<br>80% Turbuhaler;<br>58% capsule-based DPI<br><br>MDI: not shaking the inhaler and/or removing the cap (21,3%), not rinsing mouth after inhalation (18,1%)<br>Turbuhaler: not loading a dose (20%), not rinsing mouth (20%)<br>Capsule-based DPI: not rinsing mouth (22,6%)<br><br>Mean age of patient NOT compliant 10.29 +- 3.26 y (p =0.04);<br><br>No difference (p=0.88)<br><br>No difference (p=0.55)<br><br>No difference (p=0.34)<br><br>No difference (p=0.49)

References

1. Can C, Akkelle E, Gökmirza Özdemir P, Yazıcıoğlu M, Süt N. Assessment of regular drug use and inhaler technique skills in asthmatic children. Allergol Immunopathol (Madr). 2020 Mar-Apr;48(2):124-129)

2. Bickel S, Morton R, O'Hagan A, Canal C, Sayat J, Eid N. Impact of Payor-Initiated Switching of Inhaled Corticosteroids on Lung Function. J Pediatr. 2021;234:128-133.e1.

3. Almomani BA, Al-Qawasmeh BS, Al-Shatnawi SF, Awad S, Alzoubi SA. Predictors of proper inhaler technique and asthma control in pediatric patients with asthma. Pediatr Pulmonol. 2021;56(5):866–74

PICO question 6. Which patient with asthma can benefit from immunotherapy?

Patient or population: children and adolescents with asthma

Setting: tertiary care

Intervention: immunotherapy

Comparison: standard therapy

Outcome: asthma control

Search strategy:

"Asthma"[Mesh] OR "asthma" AND ("Anti-Asthmatic Agents"[Mesh] OR "Adrenergic beta-2 Receptor Agonists"[Mesh] OR "Bronchodilator Agents"[Mesh] OR "Adrenal Cortex Hormones"[Mesh] OR "inhaled steroid*" OR "LABA" OR "salmeterol" OR "treatment outcome"[Mesh] OR "allergens"[Mesh] OR "desensitization, immunologic"[Mesh])

Filters applied: last 5 years, Child: 6-12 years, Adolescent: 13-18 years

PICO n°6 Workflow of study selection process

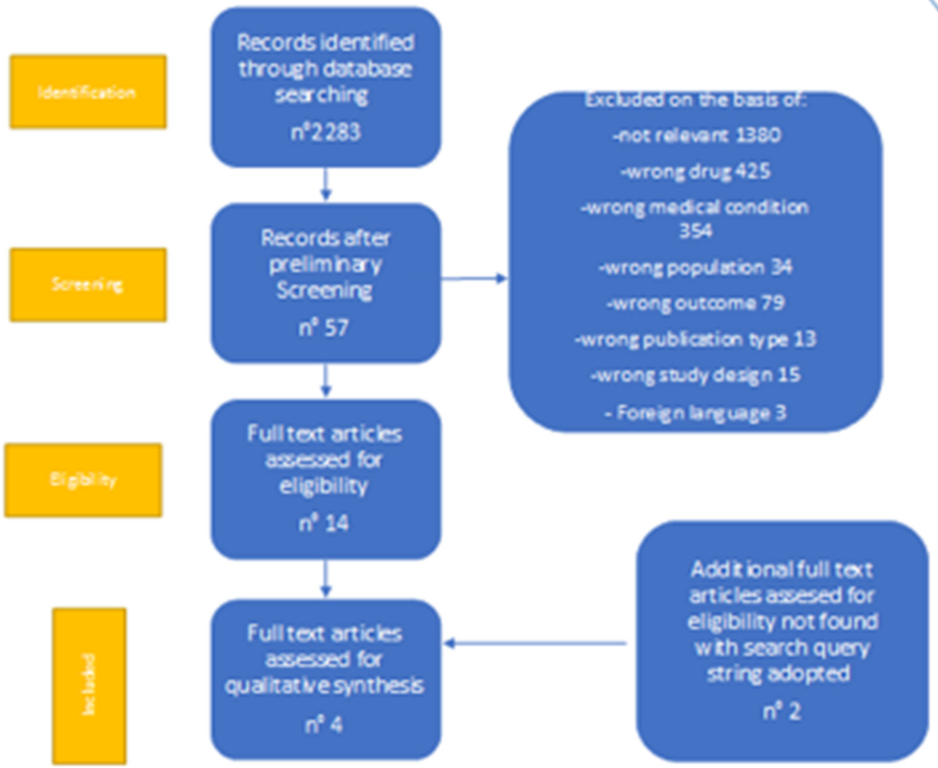

Modified from: Moher D, Liberati A, Tetzlaff J, Altman DG, The PRISMA Group (2009). Preferred Reporting Items for Systematic Reviews and Meta-Analyses: The PRISMA Statement. PLoS Med 6(7): e1000097

Title of the study, first author, year	Type of study	Study design	Population	N of patients, age	Methods	Outcomes (primary and secondary)	Results
Sublingual immunotherapy provides long-term relief in allergic rhinitis and reduces the risk of asthma: A retrospective, realworld database analysis. Zielen (2017) [1]	Retrospective case-control study	Study is a retrospective analysis of prescription data on symptomatic medications for AR and asthma. AR patients treated with grass pollen SLIT tablets were compared with a control group not having received AIT to assess the real-world, long-term efficacy of grass pollen tablets in AR and their impact on asthma onset and progression. Study consists of 3 phases: preindex period (365 days before treatment); treatment period – index date (from first to last prescription in AIT group) and follow-up period (from last	Population consists of grass-pollen allergic patients resulting in moderate-severe rhinoconjuntivitis and asthma	Global population is 74126 (adults+pediatric population).<br><br>6713 patients aged 5-17 years	Changes in prescriptions of symptomatic medications in AIT e non-AIT group during and after the immunotherapy.	The change over time in prescriptions of AR symptomatic medications after treatment cessation	The relative decrease in the mean number of AR prescriptions per year was greater in the SLIT tablet group than in the non-AIT group<br><br>RC -0.188 [CI 95% -0.222to-0.155]; p<.001;<br><br>Age < 18y: RC -0.127 [CI 95% -0.145to-0.11]; p<001
						New asthma onset, defined as the time to the first prescription of SABAs or ICSs, during treatment and after treatment cessation in patients without asthma at the index date	In the full analysis period, the proportion of initially asthma-free patients with new asthma onset was lower in the SLIT tablet group than in the non-AIT group.<br><br>**Treatment period**: OR 0.714 [CI 95% 0.547-0.932]; p=.013; Age < 18y OR 1.102 [CI 95% 0.963-1.260]; p=.159

		prescription to the end of the study). Analysis period ranged from January 2009 to February 2016				**Follow-up period**: OR 0.575 [CI 95% 0.372-0.888]; p=.013; Age < 18y OR 0.868 [CI 95% 0.729-1.033]; p=.110<br><br>**Full analysis period**: OR 0.696 [CI 95% 0.552-0.877]; p=.002; Age < 18y OR 0.973 [CI 95% 0.872-1.085]; p=.620
						The change over time in asthma medication prescriptions during the treatment and follow-up periods in patients with asthma at the index date.<br><br>The progression of asthma was consistently and significantly slower in the SLIT tablet group vs the non-AIT group<br><br>**Treatment period**: RC -0.206 [CI 95% -0.351to-0.061]; p=.005; Age < 18y RC 0.012 [CI 95% -0.060to0.085]; p=.743<br><br>**Follow-up period**: RC -0.167 [CI 95% -0.279to-0.055]; p=.004; Age < 18y RC -0.171 [CI 95% -0.227to-0.115]; p<.001<br><br>**Full analysis period**: RC -0.126 [CI 95% -0.227to-0.025]; p=.014; Age < 18y RC -0.145 [CI 95% -0.196to-0.094]; p<.001
Efficacy of house dust mite sublingual tablet in the treatment of allergic rhinoconjiunctivitis: A randomized	Multicenter, double-blind, randomized placebo-controlled study	Study compare AIT to placebo in AR. It's divided in phases: a screening period (up to 24 weeks before enrollment), a 2-	Population consists of patients with AR symptoms for > 2 years, HDM sensitized, positive allergen nasal provocation test and RTSS > 6 points for 7	438 patients aged 5-16 years. Patients were randomized 1:1 to receive placebo or HDM tablets once daily. The dose was increased from	<u>Interventions:</u><br>– HDM tablets once daily<br>– placebo	Primary efficacy end-point consists of the evaluation of AASS during weeks 48-52 of treatment; AASS (LS mean) during the last 4 weeks of treatment was significantly lower in HDM tablet group than placebo:

trial in a pediatric population<br><br>Okamoto Y., 2018 [2]		week pretreatment observation period, a 52-week treatment period and a 1-week post treatment observation period.	days before randomization.	100 IR (day 1) to 200IR (day 2) to the maintenance dose of 300IR (day 3 to week 52)		LS mean difference in AASS between groups: -0.95 +/-0.27; relative LS mean difference: -13.1%; p value = 0.0005<br><br>Additional efficacy end-points evaluated between HDM tablet and placebo group during last 4 weeks:<br><br>– ARTSS → LS mean difference +/- SE: -0.91+/-0.27; relative LS mean difference: -12.7%; p value = 0.0007<br>– ARMS → LS mean difference +/- SE: -0.006+/-0.021; p value = 0.7746<br>– ACS → LS mean difference +/- SE: -0.12+/-0.04; p value = 0.0010<br>– ISSs:<br>1. Sneezing → LS mean difference +/- SE: -0.25+/-0.08; p value = 0.0014<br>2. Rhinorrhea → LS mean difference +/- SE: -0.22+/-0.09; p value = 0.0103<br>3. Nasal congestion → LS mean difference +/- SE: -0.26+/-0.08; p value = 0.0007<br>4. Nasal pruritus → LS mean difference +/- SE: -0.18+/-0.07; p value = 0.006<br>5. Itchy eyes → LS mean difference +/- SE: -0.10+/-0.07; p value = 0.1887<br>6. Watery eyes → LS mean difference +/- SE: -0.05+/-0.05; p value = 0.3513
Immunoregulatory Effects of Subcutaneous Immunotherapy on Lymphocyte Subgroups and Cytokines in Children with	Case- control study	This study investigated the effects of SCIT on cytokine production and peripheral blood levels of lymphocyte	Population consists of children with HDM allergic asthma who had received antiasthmatic pharmacologic for 3 months at baseline.	N = 60; aged 5-10 years	<u>Intervention</u>:<br><br>– SCIT + medical treatment<br>– Only medical treatment	Comparison of TASS and TMS<br><br>After 3 months and 6 months of treatment, the change of clinical medication in the SCIT group was more obvious than that in the non-SCIT group<br><br>(p < 0,05)

Asthma. He (2019) [3]		subtypes in HDM allergic children resulting in moderate-severe asthma.				Comparison of ILC2 Percentage and IL-13 Level<br><br>SCIT induced a significant and progressive reduction in ILC2 percentage and IL-13 level after 3 and 6 months of treatment compared with baseline that was significantly higher than the non-SCIT treatment group ($p < 0,05$)
						Comparison of Th1/Th2 and IFN-γ/IL-4 Ratios.<br><br>SCIT induced a significant and progressive growth in the Th1/Th2 ratio and in the IFN-γ/IL-4 Ratio after 6 months of treatment compared with baseline that was significantly higher than the non-SCIT treatment group ($p < 0,01$)
						Comparison of the Th17/Treg. and IL-17/TGF-β Ratios<br><br>SCIT induced a significant and progressive reduction in the Th17/Treg ratio after 3 months and 6 months of treatment compared with baseline that was significantly higher than the non-SCIT treatment group ($p < 0,05$)
The moderating role of allergy immunotherapy in asthma progression.	Population-based cohort study	Longitudinal cohort study based on comprehensive routine healthcare data	Population consists of patients having asthma during the observational period.	N = 2586 (adolescentes aged 14-18 years old)	The effect of AIT on the transition between different GINA steps has been analyzed using multivariable Cox regression models	Progression of disease severity in asthma defined as a step up in asthma medication according to the GINA recommendations<br><br>The proportions of patients experiencing a step up in asthma therapy as an indicator for asthma progression

Results of a population-based cohort study<br><br>Schmitt J., 2020 [4]		from Germany. Using routine health care claims data from AOK PLUS.			adjusted for age and sex.	were lower in the subgroup of patients exposed to AIT than in patients not exposed to AIT.<br>AIT exposure is associated with a significantly decreased risk of asthma progression:<br><br>From GINA step 1 to step 3: All HR of 0,87 (95% CI 0.80-0.95); Adolescents HR of 0.72 (95% CI 0.58-0.88).<br><br>From GINA step 3 to step 4: All HR of 0,66 (95% CI 0.60-0.74); Adolescents HR of 0.76 (95% CI 0.58-0.99).
300 IR sublingual tablet is an effective, safe treatment for house dust mite-induces allergic rhinitis: An international, double-blind, placebo-controlled, randomized phase III clinical trial.<br><br>Demoly P., 2021[5]	Double-blind, placebo-controlled, randomized phase III clinical trial	If specific immunotherapy change the course of AR with moderate-severe symptoms in adolecents and adult population as compared to placebo. Trial consists of a screening phase lasting 6 weeks to 6 months, treatment phase lasting 12 months and a 2 weeks post-treatment follow-up phase.	Population sensitized to dust mites (confirmed by prick test and/or HDM-specific serum IgE level), with allergic rhinoconjunctivitis for at least 12 months before the study with moderate-severe symptoms that interfere with quality of life	1607 patients aged 12 or more; 802 receive AIT and 805 receive placebo. In particular, 312 adolescents: 155 receive 300IR tablets and 157 placebo.	<u>Intervention:</u><br>– Allergen immunotherapy (300IR tablets for HDM allergy)<br>– Placebo	Primary endopoint during the primary evaluation period(evaluable patients from the FAS) consists of aTCS differences between 300IR vs placebo groups: LS mean difference: – 0.74; IC 95% (-1.08 to – 0.38); p value 0.0001; relative LS means difference = -16.9% [IC 95%: -24.0 to -9.2]<br><br>Secondary end-points in evaluable patients from FAS between two groups:<br>– average nasal congestion symptom score: LS mean difference: – 0.19; IC 95% (-0.28 to – 0.10); p value <0.0001; relative LS means difference = -18.3%<br>– overall RQLQ12+ score: LS mean difference: – 0.19; IC 95% (-0.30 to – 0.09); p value 0.0004; relative LS means difference = -12%<br>– average RMS: LS mean difference: – 0.09; IC 95% (-0.14 to – 0.04); p value 0.0004; relative LS means difference = -29.7%<br>– average CSMS: LS mean difference: – 0.26; IC 95% (-0.38 to – 0.14); p value <0.0001; relative LS means difference = -18%<br>– average RCTSS: LS mean difference: – 0.81; IC 95% (-1.24 to – 0.39); p value 0.0002; relative LS means difference = -16.1%

ICS/LABA Combined With Subcutaneous Immunotherapy Modulates the Th17/Treg Imbalance in Asthmatic Children.<br>Dai, 2022 [6]	Case-control study	Immunologic changes analyzed on blood samples in asthmatic children treated by ICS/LABA powder inhalation compare to asthmatic children treated by ICS/LABA powder inhalation combined with HDM-SCIT. Study lasted 6 months	15 healthy children and 30 HDM allergic children with moderate to severe asthma who needed inhaled ICS/LABA treatment	45 children aged 5-12 years old	Interventions:<br>– ICS/LABA powder inalation<br>– ICS/LABA powder inalation + HDM-SCIT<br>– Control group	**Effects of ICS/LABA on Th17/Treg balance**<br><br>Th17/Treg ratio after ICS/LABA treatment:<br>0.194 ± 0.025 vs. 0.439 ± 0.072 ($p<0.01$)<br><br>**Effects of ICS/LABA + HDM-SCIT on Th17/Treg balance**<br><br>Th17/Treg ratio after ICS/LABA+HDM-SCIT treatment:<br>0.133 ± 0.015 vs. 0.419 ± 0.049 ($p<0.01$)<br><br>**ICS/LABA vs ICS/LABA + HDM-SCIT on Th17/Treg balance**<br><br>Th17/Treg ratio in ICS/LABA group vs ICS/LABA+SCIT group:<br>0.133 ± 0.015 vs 0.194 ± 0.025; $p<0.01$<br><br>Treg cell in ICS/LABA group vs ICS/LABA+SCIT group:<br>8.483 ± 0.408% vs. 6.833 ± 0.485%; $p<0.05$<br><br>IL-10 in ICS/LABA group vs ICS/LABA+SCIT group:<br>127.4 ± 4.423 pg/ml vs. 99.34 ± 6.496 pg/ml; $p<0.01$

AR = allergic rhinitis

RC = regression coefficient

SCIT = subcutaneous immunotherapy

SLIT = sublingual immunotherapy

HDM = house dust mite

AIT = allergen immunotherapy

AASS = average adjusted symptom score

RTSS = rhinitis total symptom score

ARMS = average rescue medication score

ISSs = individual symptom scores

ACS = average combined score

IR = index of reactivity

TASS = total asthma symptom score

TMS = total medication score

ILC2 = type 2 innate lymphoid cells

aTCS = average total combined score

LS = least squares

FAS = full analyses test

CSMS = combined symptom and medication score

RCTSS = rhinoconjunctivitis total symptom score

RQLQ12+ = standardized rhinoconjunctivitis quality of life questionnaire for 12 years and older

References

1. Zielen S, Devillier P, Heinrich J, Richter H, Wahn U. Sublingual immunotherapy provides long-term relief in allergic rhinitis and reduces the risk of asthma: A retrospective, real-world database analysis. Allergy. 2018 Jan;73(1):165-177. doi: 10.1111/all.13213. Epub 2017 Aug 10.

PMID: 28561266; PMCID: PMC5763412.

2. Okamoto Y, Fujieda S, Okano M, Hida H, Kakudo S, Masuyama K. Efficacy of house dust mite sublingual tablet in the treatment of allergic rhinoconjunctivitis: A randomized trial in a pediatric population. Pediatr Allergy Immunol. 2019;30(1):66-73. doi:10.1111/pai.12984
3. He YT, Zhou Y, Shao Q, Gan C, Chen M, Bao YL, Gu HY, Zhang SL, Cui Y, Tian M. Immunoregulatory Effects of Subcutaneous Immunotherapy on Lymphocyte Subgroups and Cytokines in Children with Asthma. J Immunol Res. 2019 Oct 13;2019:7024905. doi: 10.1155/2019/7024905. PMID: 31737687; PMCID: PMC6815992.
4. Schmitt J, Wüstenberg E, Küster D, Mücke V, Serup-Hansen N, Tesch F. The moderating role of allergy immunotherapy in asthma progression: Results of a population-based cohort study. Allergy. 2020 Mar;75(3):596-602. doi: 10.1111/all.14020. Epub 2019 Sep 8. PMID: 31408535.
5. Demoly, Pascal et al. “A 300 IR sublingual tablet is an effective, safe treatment for house dust mite-induced allergic rhinitis: An international, double-blind, placebo-controlled, randomized phase III clinical trial.” The Journal of allergy and clinical immunology vol. 147,3 (2021): 1020-1030.e10. doi:10.1016/j.jaci.2020.07.036
6. Dai H, Zheng R, Wang L, Wan J, Tong Y, Zhao W, Zhang W. ICS/LABA Combined With Subcutaneous Immunotherapy Modulates the Th17/Treg Imbalance in Asthmatic Children. Front Immunol. 2022 Mar 10;13:779072. doi: 10.3389/fimmu.2022.779072. PMID: 35355985; PMCID: PMC8960042.

PICO question 7. In children with uncontrolled asthma symptoms despite daily medium dose ICS combined with LABA, is increasing the dose of ICS more effective than adding the long-acting muscarinic antagonist (LAMA) tiotropium?

Patient or population: children and adolescents with asthma

Setting: tertiary care

Intervention: increasing ICS

Comparison: adding LAMA

Outcome: symptom control and lung function

Search strategy:

"Asthma"[Mesh] OR "asthma" AND ("Anti-Asthmatic Agents"[Mesh] OR "Adrenergic beta-2 Receptor Agonists"[Mesh] OR "Bronchodilator Agents"[Mesh] OR "Adrenal Cortex Hormones"[Mesh] OR "inhaled steroid*" OR "LABA" OR "salmeterol" OR "muscarinic antagonists"[Mesh] OR "LAMA" OR "tiotropium bromide"[Mesh])

Filters applied: last 5 years, Child: 6-12 years, Adolescent: 13-18 years

PICO n°7 – Workflow of study selection process

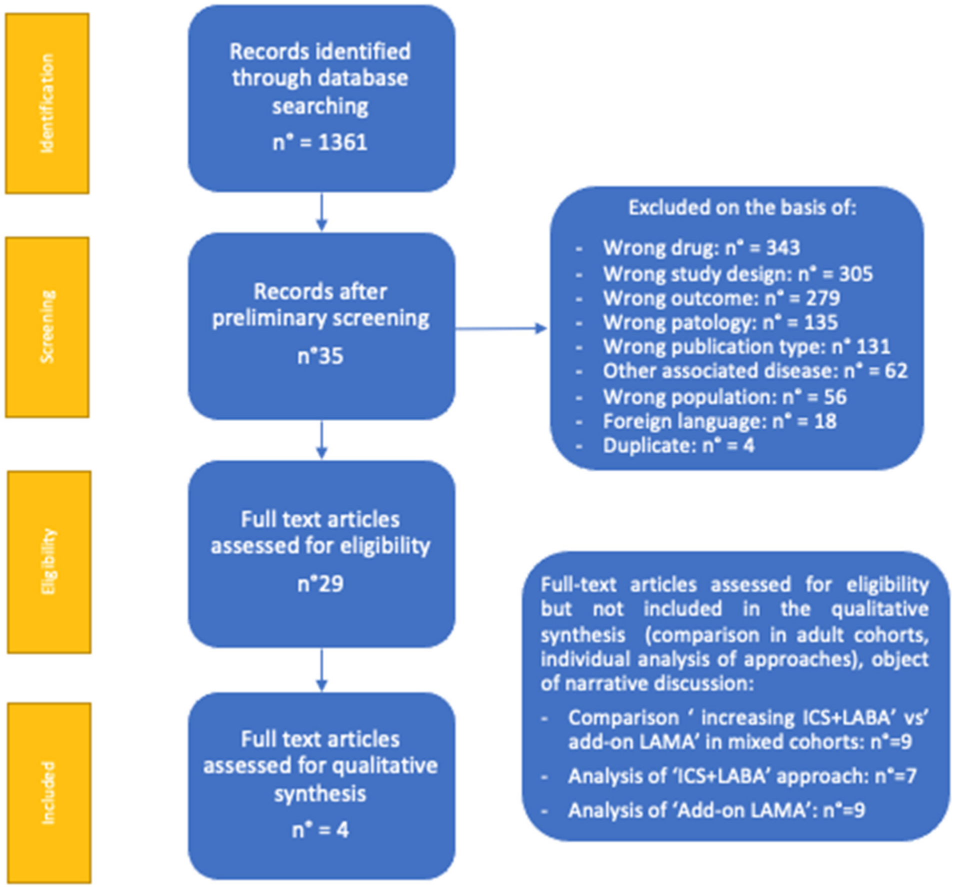

Modified from: Moher D, Liberati A, Tetzlaff J, Altman DG, The PRISMA Group (2009). Preferred Reporting Items for Systematic Reviews and Meta-Analyses: The PRISMA Statement. PLoS Med 6(7): e1000097

Title of the study, first author, year	Type of the study	Study design	Population	N° of patients, age	Methods	Outcomes	
Efficacy and safety of 2 doses of Tiotropium Respimat® compared to placebo in children with moderate persistent asthma.<br>Vogelberg C, 2018 [1]	Phase 3, double-blind, placebo-controlled, parallel-group study	To evaluate efficacy and safety of two different doses of tiotropium (5 ug/day; 2.5 ug/day) in patients with moderate symptomatic asthma over 48 weeks	Children treated with:<br>- medium-dose ICS (200-400 mg of budesonide or equivalent) with or without LTRA;<br>- LABA treatment was stopped at least 24 hours before the 4-wk run-in period;<br>- treatment with sustained-release theophylline was not allowed	n = 403 (6-11 years of age)	Intervention:<br>- Tiotropium low dose (2.5 ug/day)<br>- Tiotropium high dose (5 ug/day)<br>- Placebo	Primary outcome (adjusted mean difference vs placebo)	
						Peak FEV_1 (0-3h) at week 24:	Tio 5ug: 164 mL (95% CI, 103-225 mL); P < 0.001<br>Tio 2.5 ug: 170 mL (95% CI, 108-231 mL); P < 0.001
						Secondary outcome (lung function)	
						Trough FEV_1 at week 24	Tiotropium, 5 ug: 118 mL (95% CI, 48 to 188 mL); P= 0.001<br>Tiotropium, 2.5 mg: 116 mL (95% CI, 46-186 mL); P=0.001

						Peak FEV1 (0-3h) at week 48	Tiotropium, 5 ug: 127 mL (95% CI, 65-188 mL); P < 0.001<br><br>Tiotropium, 2.5 ug: 124 mL (95% CI, 62-185 mL); P<0.001
						Trough FEV1 at week 48	Tiotropium, 5 ug: 99 mL (95% CI, 29-170 mL); P = 0.006<br><br>Tiotropium, 2.5 ug: 71 mL (95% CI, 1-142 mL); P =0.048
						Peak FVC (0-3h) at week 24 (mL)	Tiotropium, 5 ug: 91 (95% CI: 18, 165; P=0.015<br><br>Tiotropium, 2.5 ug: 110 (95% CI: 36, 184; P=0.004)

						Trough FVC at week 24	Tiotropium, 5 ug: 52 mL (95% CI, 27 to 131 mL); P=0.20<br>Tiotropium, 2.5 ug: 92 mL (95% CI, 13-171 mL); P = 0.02
Add-on tiotropium versus step-up inhaled corticosteroid plus long-acting beta-2-agonist in real-world patients with asthma. Chipps B., 2020 [2]	Retrospective cohort study	To compare the effectiveness of add-on tiotropium versus an increased ICS plus LABA dose in patients with asthma in therapy with ICS plus LABA	Patients with asthma from the IMS LifeLink PharMetric Plus (North Carolina) and the EMRClaim+ (New York) databases between January 2014 and December 2018. All patients in therapy with ICS plus LABA	7.857, aged ≥12 years	The cohort of patients was followed-up and divided into two groups. One group received tiotropium Respimat 1.25 ug (Tio group) and the other group had their ICS plus LABA dose increased (inc-ICS group)	Primary outcome	
						Risk of exacerbation	35% lower in Tio group vs the inc-ICS group (Hazard ratio 0.65 [95% CI, O.43-0.99]; p<0.05)
						Secondary outcome	
						Rate of exacerbation within 6 months postindex	64% lower in Tio group vs the inc-ICS group (41.4 vs 116.1 cases per 100 person-years, p<0.0001)

						Rate of exacerbation within 12 months postindex	73% lower in Tio group vs the inc-ICS group (15.7 vs 57.2 cases per 100 person-years, p<0.0001)
						Health-care resource utilization	
						All-cause visit rate	47% lower in Tio group than in the inc-ICS group, p<0.0001
						Asthma-related ED visit rate	74% lower in Tio group than in the inc-ICS group, p<0.0001
						All-cause hospitalization rate	48% lower in Tio group than in the inc-ICS group, p<0.01

						Asthma-related hospitalization rate	76% lower in Tio group than in the inc-ICS group, p<0.001
						Short-acting beta2 agonist (SABA) refills within 12-month postindex	Lower in Tio group (56%) than in the inc-ICS group (67%), p<0.0001
Triple vs Dual Inhaler Therapy and Asthma Outcomes in Moderate to Severe Asthma. Kim LHY, 2021 [3]	Systematic review and Meta-analysis	To systematically synthesize the outcomes and adverse events associated with triple therapy (ICS, LABA and LAMA) vs dual therapy (ICS plus LABA) in	RCTs comparing triple vs dual therapy from MEDLINE, Embase, CENTRAL, ECTRP, FDA and EMA database from November 2017	11.894 children and adults (mean age 52 years, range 9-71 years)	Random-effects meta-analyses	Severe exacerbation risk (9 trials [9932 patients])	22.7% with triple therapy vs 27.4% with dual therapy; RR 0.83 [95% CI, 0.77-0.90]

		children and adults with persistent uncontrolled asthma	to December 2020			Asthma control (14 trials [11 230 patients])	Triple therapy associated with an improvement in asthma control scores compared with dual therapy: standardized mean difference [SMD], −0.06 [95% CI, −0.10 - −0.02]; mean difference in ACQ-7 scale, −0.04 [95% CI, −0.07 - −0.01]
						Asthma-related quality of life (7 trials [5247 patients]	Triple therapy was not significantly associated with an improvement in asthma-related quality SMD, 0.05 [95% CI, −0.03 - 0.13]; mean difference in AQLQ score, 0.05 [95% CI, −0.03 to 0.13]

						Mortality (17 trials [11 595 patients]	No significant difference in all-cause mortality between two groups: 0.12% vs 0.12%; RR 0.96 [95% CI, 0.33 - 2.75]
						Serious adverse events (12 trials [11505 patients]	No significant difference between two groups: 5.2% vs 5.6%, RR 0.92 [95% CI, 0.73 – 1.16]
						Spirometry Parameters – FEV1 (18 trials [11 715 patients]	Triple therapy was significantly associated with an improvement in FEV1; mean difference, 0.08 L [95% CI, 0.07 -0.10]

Effectiveness and tolerability of dual and triple combination inhaler therapies compared with each other and varying doses of inhaled corticosteroids in adolescents and	Systematic review and Meta-analysis	To assess the effectiveness and safety of dual (ICS/LABA) and triple therapies (ICS/LABA/LAMA) compared with each other and with varying	RCTs of at least 12 weeks of study duration from 2008 to 18 February 2022, including adolescents and adults with uncontrolled asthma who had been treated	17.161 from 17 studies (mean age 49.1 years)	Cochrane's Screen4ME workflow to assess search results and Grading of Recommendations Assessment, Development and Evaluation (GRADE) to assess the certainty of evidence, comparing dual and triple therapies	Primary outcomes

adults with asthma: a systematic review and network meta-analysis.<br>Oba Y., 2022 [4]		doses of ICS in adolescents and adults with uncontrolled asthma	with, or were eligible for, MD-ICS/LABA			Steroid-requiring asthma exacerbations	Medium-dose (MD) and high-dose (HD) triple therapies reduce steroid-requiring asthma exacerbations (HR 0.84 [95% CI 0.71 - 0.99] and 0.69 [95% CI 0.58 - 0.82], respectively).High-dose triple therapy likely reduces steroid-requiring asthma exacerbations compared to MD triple therapy (HR 0.83 [95%CI 0.69 - 0.996]

						Asthma-related hospitalisations	No statistical difference between triple therapy compared to MD-ICS/LABA
						Secondary outcomes	
						All-cause adverse events (AEs)	Reduction in HD triple therapy, but not MD triple groups compared to MD-ICS/LABA therapy group (OR 0.79 [95% CI 0.69 - 0.90])

						Dropouts due to AEs	Reduction in HD triple therapy, but not MD triple groups compared to MD-ICS/LABA therapy group (OR 0.50 [95% CI 0.30 - 0.84])

References

1. Vogelberg C, Engel M, Laki I, Bernstein JA, Schmidt O, El Azzi G, et al. Tiotropium add-on therapy improves lung function in children with symptomatic moderate asthma. J Allergy Clin Immunol Pract. 2018;6: 2160–2162.e9.

2. Chipps B, Mosnaim G, Mathur SK, Shaikh A, Khoury S, Gopalan G, et al. Add-on tiotropium versus step-up inhaled corticosteroid plus long-acting beta-2-agonist in real-world patients with asthma. Allergy Asthma Proc. 2020;41: 248–255.

3. Kim LHY, Saleh C, Whalen-Browne A, O'Byrne PM, Chu DK. Triple vs Dual Inhaler Therapy and Asthma Outcomes in Moderate to Severe Asthma: A Systematic Review and Meta-analysis. JAMA. 2021;325: 2466–2479.

4. Oba Y, Anwer S, Maduke T, Patel T, Dias S. Effectiveness and tolerability of dual and triple combination inhaler therapies compared with each other and varying doses of inhaled corticosteroids in adolescents and adults with asthma: a systematic review and network meta-analysis. Cochrane Database Syst Rev. 2022;12: CD013799.

PICO question 8. Considering the biologics for severe asthma, which are the differences among omalizumab, mepolizumab and dupilumab?

Patient or population: children and adolescents with asthma

Setting: tertiary care

Intervention: biologics

Comparison: standard therapy

Outcome: biologic efficacy

Search strategy:

"Asthma"[Mesh] OR "asthma" AND ("Anti-Asthmatic Agents"[Mesh] OR "Adrenergic beta-2 Receptor Agonists"[Mesh] OR "Bronchodilator Agents"[Mesh] OR "Adrenal Cortex Hormones"[Mesh] OR "inhaled steroid*" OR "LABA" OR "salmeterol" OR "treatment outcome"[Mesh] OR "biologics" OR "biologicals" OR "antibodies, monoclonal"[Mesh])

Filters applied: last 5 years, Child: 6-12 years, Adolescent: 13-18 years

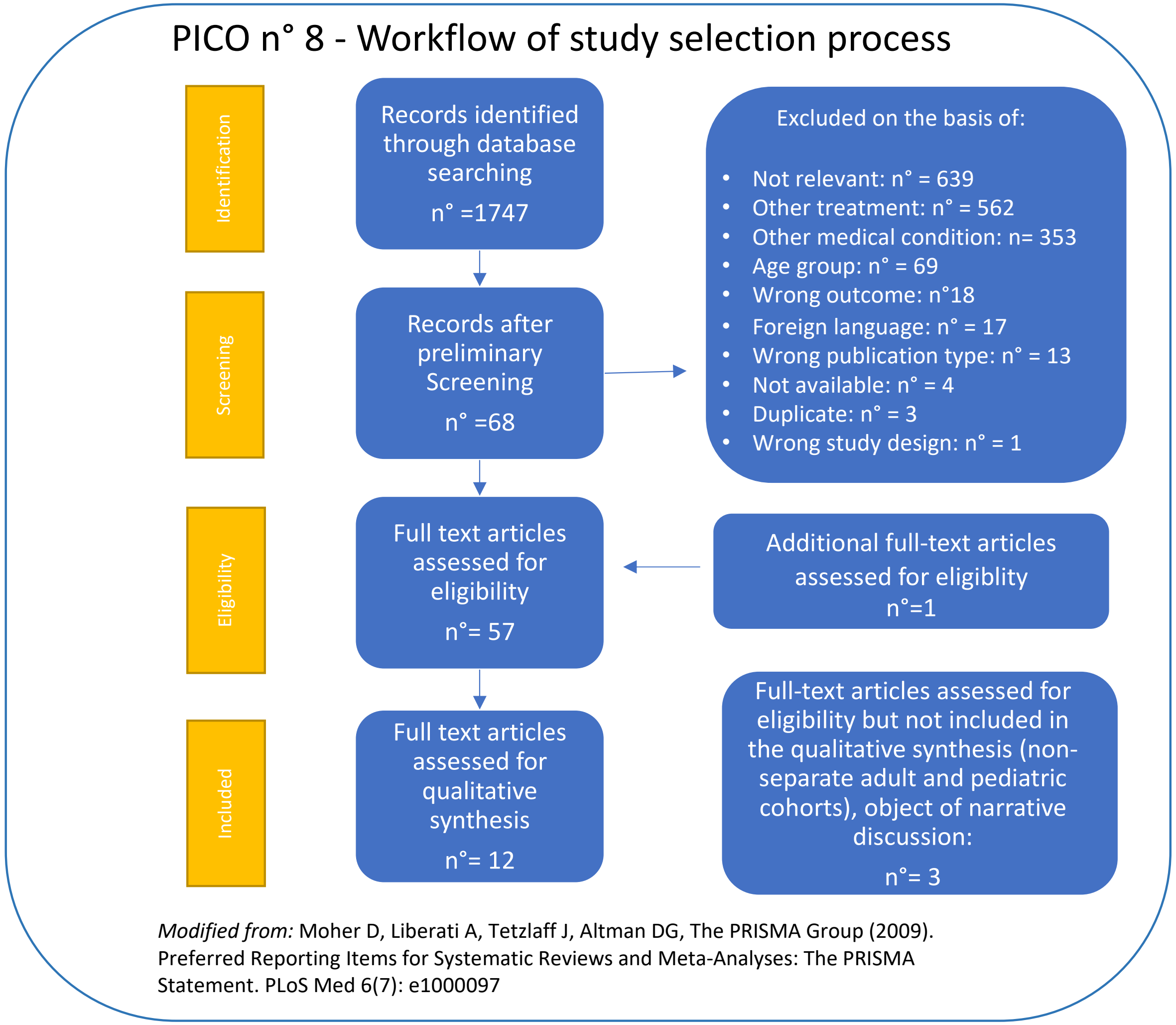
PICO n° 8 - Workflow of study selection process
Identification
Screening
Eligibility
Included
Records identified through database searching
n° =1747
Records after preliminary Screening
n° =68
Full text articles assessed for eligibility
n°= 57
Full text articles assessed for qualitative synthesis
n°= 12
Excluded on the basis of:
Not relevant: n° = 639
Other treatment: n° = 562
Other medical condition: n= 353
Age group: n° = 69
Wrong outcome: n°18
Foreign language: n° = 17
Wrong publication type: n° = 13
Not available: n° = 4
Duplicate: n° = 3
Wrong study design: n° = 1
Additional full-text articles assessed for eligiblity
n°=1
Full-text articles assessed for eligibility but not included in the qualitative synthesis (non-separate adult and pediatric cohorts), object of narrative discussion:
n°= 3
Modified from: Moher D, Liberati A, Tetzlaff J, Altman DG, The PRISMA Group (2009). Preferred Reporting Items for Systematic Reviews and Meta-Analyses: The PRISMA Statement. PLoS Med 6(7): e1000097

Title of the study, first author, year	Type of study	Study design	Population	N° of patients, age	Methods	Outcomes	
Omalizumab effectiveness in patients with severe allergic asthma according to blood eosinophil count: the STELLAIR study. Humbert, 2018 [1]	Multi-center, non-interventional, retrospective, observational study	Response to omalizumab prescribed as an add-on therapy, after 4-6 months of treatment (T_{4-6}) compared with the data recorded during the 12 months prior to omalizumab initiation.	Pediatric patients with severe allergic asthma.	149 (6-17 years, subgroup of 879 patients)	3 criteria: physician evaluation, with the Global Evaluation of Treatment Effectiveness (GETE) scale, Reduction of ≥40% in annual exacerbation rate and the combination of both. Documented blood eosinophils within 12 months prior to omalizumab initiation	Omalizumab appears to be as effective in patients with "high" eosinophils (≥300 cells/µl) as in those with "low" eosinophils (<300 cells/µl).<br>-Using the GETE scale 77.2% were responders<br>-Reduction ≥40% in annual exacerbation rate observed in 78.5% of patients<br>-Combined response: 70.9% in minors with an eosinophils ≥300 cells/µL, 59% in those with an eosinophils <300 cells/µL.<br>These results remain similar with all other blood EOS cut-offs studied and for all definitions of response.	95% CI, 71.1to84.8<br><br>95% CI, 61.5to79.2<br><br>95% CI, 42.1to74.4
Omalizumab Effectiveness by Biomarker Status in Patients with	Multicenter, prospective, 48-week effectivene	Outcomes after omalizumab initiation: data collected at baseline (12	Pediatric patients with moderate to severe allergic asthma,	69 (12-17 years, subgroup of 806 patients)	Exacerbation rate<br><br>Percentage of patients with ≥	Improved from a mean baseline of 2.80 ± 2.64 to a rate of 0.46 ± 0.82 through month 12.<br><br>Decreased from 28% in the 12 months	P <0.001

Asthma: Evidence From PROSPERO, A Prospective Real-World Study. Casale, 2019 [2]	ss study	months before study entry, T_{-12}) and through 12 months on study. (T_{12}). Omalizumab planned dosing frequency was almost evenly split between every 2 and 4 week dosing	eligible for omalizumab	69 receiving ≥1 dose of omalizumab and 59 patients completing study.	1 hospitalizations<br><br>Lung function<br><br>-ACT score	before baseline to 4% during the 12 months of omalizumab.<br><br>Relative unchanged:<br>-Mean postbronchodilator FEV1: 2.79 L at baseline and 3.00 L at month 12.<br>- Prebronchodilator FEV1 improved by 170 mL.<br><br>Mean improvement of 3.9 ± 5.0.	
The clinical benefit of mepolizumab replacing omalizumab in uncontrolled severe eosinophilic asthma. Chapman, 2019 [3]	Multi-center, open-label, single-arm, 32-week trial	1-4 week run-in period (all maintenance therapy, including omalizumab, was continued throughout the run-in period). At baseline study visit discontinuation of omalizumab treatment and change to mepolizumab 100 mg subcutaneously every 4 weeks for 32 weeks (final dose Week 28)	Patients with uncontrolled severe eosinophilic asthma (peripheral blood eosinophil count ≥150 cells/µl at enrollment or ≥300 cells/µl in the 12 months before) and ≥2 asthma exacerbations in the year prior to enrollment despite receiving high-dose inhaled	145 patients (1% of patients are between 12 and 17 years old)	-ACQ-5 score<br><br>St George's Respiratory Questionnaire (SGRQ) score<br><br>Exacerbations<br><br>Pre-bronchodilator FEV_1	Improved with mepolizumab treatment: from an LS mean score of 3.20 to 1.75.<br>At w 32 the LS mean change was -1.45 points with 77% of patients achieving the minimum clinically important difference (ACQ-5: ≥0.5 points).<br><br>Improved with mepolizumab treatment: from an LS mean score of 56.7 to 37.8.<br>At w 32 the LS mean change was -19 points with 79% of patients achieving the minimum clinically important difference (SGRQ: ≥4 points).<br><br>Annualized rates of clinically significant exacerbations were reduced by 64%.<br>Exacerbation requiring an ER visit/hospitalization were reduced by 69%.<br><br>Improved with mepolizumab treatment: from an LS mean 1755 mL to 1915 mL, with a LS mean change of 159 mL.	

			corticosteroids and other controller, plus omalizumab (≥4 months)		Post- bronchodilator FEV_1	Improved with mepolizumab treatment: from 1987 mL to 2106 mL, with a LS mean change of 120 mL.	
'Real-life' experience in asthmatic children treated with omalizumab up to six-years follow-up. Folqué, 2019 [4]	Observational single center 'real-life' study	Evolution of patients treated with omalizumab. The dose administered ranged from 150-200 mg per month. The frequency of administration was every two weeks in 34 cases and monthly in 14. Exacerbations requiring visit to the emergency department and hospital admission were collected starting the year prior to omalizumab (T_{-12}), six months after starting treatment (T_6), and the annually (T_{12}, T_{24}, $T_{36,}$	Children with severe uncontrolled asthma (according to GINA).	48 (5-17 years)	Admission for asthma exacerbations<br><br>Visits to the emergency department<br><br>Dose of fluticasone<br><br>Use of LABA<br><br>FEV_1<br><br>$FEF_{25-75\%}$	Decreased over the years: admission rate per 100 patients/year of 45.8 at T_{-12}; 8.5 at T_6; 3.9 at T_{24}; 0 from T_{36} to $T_{72.}$<br><br>Decreased over the years: admission rate per 100 patients/year of 110.41 at T_{-12}; 29.8 at T_6; 14.3 at T_{24}; 0 at $T_{36.}$<br><br>Decreased over the years: from 452 mcg/day at T_{-12} to 329.89 mcg/day at T_6; this difference was maintained through the follow-up.<br><br>Decreased over the years: 98% of the patients requiring LABA at T_{-12}; 86.96% at T_6; 75% at T_{12}; the difference was maintained through the follow-up.<br><br>Increased over the years: mean value of 79.88 at T_{-12}; 92.99 at T_6; values were maintained above normal for the course of the treatment.<br><br>Increased over the years: mean value of	p=0.02<br>p=0.017<br><br>p=0.007<br>p=0.001<br><br>p=0.0001<br><br>p=0.074<br>p=0.008<br><br>p=0.0001<br><br>*p*=0.0001

		T_{48}, T_{60}, T_{72})				62.94 at T_{-12};<br>76.31 at T_{6};<br>values were maintained above normal for the course of the treatment.	
Clinical impact of omalizumab treatment in children with severe asthma. Report of a local experience. Giubergia, 2019 [5]	Prospective, longitudinal (pre-/post-intervention), observational, analytical study	Treatment with Omalizumab between August 2012 and December 2017. The dose and the frequency of administration (monthly or biweekly) were determined based on weight and IgE levels.	Children with uncontrolled severe asthma, with criteria for omalizumab indication	17 (8-16 years)	Patients with asthma attacks	Decreased after omalizumab treatment from 94% to 59%	*p* =0.005
					Patients with severe attacks	Decreased after omalizumab treatment from 59% to 0%	*p* =0.0002
					Exacerbations	Reduced after omalizumab treatment by 48.5%	*p* =0.009
					Severe exacerbations	Reduced after omalizumab treatment by 100%	*p* = 0.001
					Hospitalized patients	Decreased after omalizumab treatment from 35% to 0%	*p* =0.004
					Hospitalizations per patients	Decreased after omalizumab treatment from 0.5 (95% CI 0-12-0.9) to 0.	*p* =0.007
					Days of OCS in the treatment of asthma attacks	Decreased after omalizumab treatment from 12 (95% CI 5.5-18) to 4.5 (95% CI 2-7)	*p* =0.03
					Days of salbutamol in the treatment of asthma attacks	Decreased after omalizumab treatment from 27.4 (95% CI 9.8-45.1) to 9.3(95% CI 1.8-16.7)	*p* =0.002
					Daily ICS doses CI in the chronic treatment	Decreased after omalizumab treatment from 1053.3 µg (95% CI 1002-1104) to 846.6 mcg (95% CI 697-996)	*p* =0.002
					Patients using OCS in the chronic treatment	Decreased after omalizumab treatment from 29.4% to 6%	*p* =0.01

					FVC<br><br>FEV_1<br><br>FEV_1/FVC<br><br>MMFEF	Decreased after omalizumab treatment from 106 (95% CI 98-114) to 101 (95% CI 93-109)<br><br>Decreased after omalizumab treatment from 95 (95% CI 84-105 to 90 (95% CI 80-99)<br><br>No difference after omalizumab treatment: from 80 (95% CI 73-88) to 80 (95% CI 70-86)<br><br>No difference after omalizumab treatment: from 73 (95% CI 53-94) to 73 (95% CI 51-95)	*p* =0.1<br><br>*p* =0.1<br><br>*p* =0.2<br><br>*p* =0.4
Long-term safety and pharmacodynamics of mepolizumab in children with severe asthma with an eosinophilic phenotype.Gupta, 2019 [6]	Open-label, uncontrolled, repeat-dose extension to a phase II study	Treatment with a body weight-dependent dose of subcutaneous mepolizumab of 40 mg (<40 kg) or 100 mg (≥ 40 kg) over 52 weeks	Children with severe asthma with an eosinophilic phenotype (blood eosinophil ≥150 cells/µl at screening or ≥300 cells/µl in the previous year)	30 (6-11 years)	On-treatment exacerbation rate<br><br>ACQ-7 score<br><br>ACQ-5 score<br><br>C-ACT score	69% lower than baseline: from a mean of 3.5 events/year to a mean of 1.09 events/year<br><br>Decreased from 1.79 before receiving mepolizumab to 0.79 at week 36 and increased to 1.14 at w 52<br><br>Decreased from 1.87 before receiving mepolizumab to 0.79 at week 36 and increased to 1.08 at w 52<br><br>Increased from 17.6 before receiving mepolizumab to 22 at week 36 and decreased to 20.05 at w 52	95% CI, 0.63 to 1.89<br><br>95% CI, 1.39 to 2.19 at baseline; 95% CI, 0.51 to 1.06 at w 36; 95% CI, 0.79 to1.49 at w 52<br><br>95% CI, 1.44 to 2.31 at baseline; 95% CI, 0.51 to 1.07 at w 36; 95% CI, 0.64 to1.52 at w 52<br><br>95% CI, 15.8 to 19.4 at baseline; 95% CI, 20.7 to 23-3 at w 36; 95% CI,

					Mean blood eosinophil count	Decreased from 366 cells/µl before the first mepolizumab treatment in part A of the study to 47 cells/µl at 52 w (overall study w 72)	18.8 to 22.2 at w 52
Subcutaneous mepolizumab in children aged 6 to 11 years with severe eosinophilic asthma. Gupta, 2019 [7]	Non-randomized, open-label, repeat-dose, phase II study	Treatment with mepolizumab every 4 weeks for a total of three doses (week 0, 4, and 8) with the study active treatment period defined as weeks 0 to 12. Mepolizumab 40 mg for children <40 kg and mepolizumab 100 mg for children ≥40 kg.	Pediatric patients with severe eosinophilic asthma (peripheral blood eosinophil counts ≥300 cells/µl <12 months of screening or ≥ 150 cells/µl at screening) and ≥ 2 exacerbation the prior year	36 (6-11 years)	-Blood eosinophil counts<br><br>-ACQ-7 score<br><br>C-ACT score<br><br>Prebronchodilator FEV_1	Reduced from baseline:<br>-by 88.5% in the 40 mg dose group (386 cells/µl to 42 cells/µl)<br>-by 83.4% in the 100 mg dose group (331 cells/µl to 55 cells/µl)<br><br>Decreased from baseline.<br>A minimally clinically important improvement (≥0.5-point reduction) was reported for:<br>-48% of children in the 40 mg dose group (-0.41)<br>-50% of children in the 100 mg dose group (0.08)<br><br>Increased from baseline.<br><br>No clear pattern of change from baseline.	95% CI, 26 to 67<br>95% CI, 31 to 97<br><br>95%CI, -0.91to0.08<br>95%CI, -0.88to1.04
Dupilumab in Children with Uncontrolled Moderate-to-Severe Asthma. Bacharier, 2021 [8]	Randomized, double-blind, placebo-controlled trial	Injection of Dupilumab (at a dose of 100 mg for those weighing ≤30 kg and 200 mg for those weighing > 30 kg) vs placebo.	Children with moderato-to-severe asthma (according to GINA)	408 (6-11 years)	Annualized rate of severe asthma exacerbations<br><br>Mean predicted prebronchodilator FEV1	Lower in the dupilumab group:<br>-0.31 in the dupilumab group<br>-0.75 in the placebo group<br><br>Major improvement in the dupilumab group at w 12.<br>-from 77.7±14.4 at baseline to 87.8±14.6 in the dupilumab group<br>-from 78.4±14.5 at baseline to 83.2±15.5 in	95% CI, 0.22to0.42<br>95% CI, 0.54to1.03<br><br>RR reduction in the dupilumab group, 59.3% (95% CI, 39.5 to 72.6; *p*<0.001)

					ACQ-7-IA Score<br><br>FE_{NO}<br><br>Time until the first severe exacerbation<br><br>Risk of loss of asthma control	the placebo group<br><br>Significantly better asthma control with dupilumab than placebo at w 24.<br>- -1.33±0.05 in the dupilumab group<br>- -1.00±0.07 in the placebo group<br><br>Higher decrease at w 12 in the dupilumab group.<br><br>Longer in the dupilumab group.<br><br>Lower in the dupilumab group.	LS Mean Difference vs. Placebo: 4.7 (95% CI, 1.9 to 7.5) *p*<0.001<br><br>LS Mean Difference vs. Placebo: -0.28 (95% CI, -0.44 to -0.12) *p*<0.001<br><br>-patients with type 2 phenotype: hazard ratio, 0.44 (95% CI, 0.29 to 0.67)<br>-patients with blood eosinophil > 300 eosinophils/mm^3 :hazard ratio 0.38 (95% CI, 0.23 to 0.63)<br><br>-patients with type 2 phenotype: hazard ratio, 0.69 (95% CI, 0.52 to 0.9)<br>-patients with blood eosinophil > 300 eosinophils/mm^3 :hazard ratio 0.66 (95% CI, 0.48 to 0.90)

Omalizumab outcomes for up to 6 years in pediatric patients with severe persistent allergic asthma. Garcia, 2021 [9]	Multicenter, observational, retrospective cohort study	Data collected between 2006 and 2018, from the year prior to omalizumab initiation (T_{-12}) to discontinuation or last available follow-up	Pediatric patients with severe persistent allergic asthma and unable to achieve disease control	426 (<18 years)	Moderate-to-severe exacerbations	Decreased by 80.2% from T_{-12} to the 1°year. The number continued low up to Year 6.	95% CI -84.5% to -75.8%
					FeNO	Decreased significantly during the 1°year, remaining stable the following years.	Mean ppb: T_{-12} 44.5; 1° year 28.4; 2° year 28.7; 3° year 30.5; 4° year 27.9; 5° year 25.2; 6° year 27.8
					FEV_1	Increased significantly during the 1°year, remaining stable the following years.	Mean % predicted: T_{-12} 84.6; 1° year 92.3; 2° year 92.4; 3° year 91.8; 4° year 91.4; 5° year 93.6; 6° year 92.8
					Emergency visits	Decreased significantly during the 1°year, remaining stable the following years.	T_{-12} 3.6; 1° year 0.5; 2° year 0.4; 3° year 0.4; 4° year 0.2; 5° year 0.2; 6° year 0.1
					Hospitalizations	Decreased significantly during the 1°year, remaining stable the following years.	T_{-12} 1.0; 1° year 0.1; 2° year 0.1; 3° year 0; 4° year 0; 5° year 0; 6° year 0
					PICU admissions	Decreased significantly during the 1°year, with no PICU admissions from years 2 onwards.	T_{-12} 0.1; 1° year 0; 2° year 0; 3° year 0; 4° year 0; 5° year 0; 6° year 0
Real-life long-term safety	104-week, multicenter	Post marketing surveillance	Japanese pediatric	127 (6-15 years)	Adverse drug reactions	10.2%	

and effectiveness of omalizumab in Japanese pediatric patients with severe allergic asthma: A post-marketing surveillance. Nakamura, 2021.<br>[10]	surveillanc e.	conducted over six years in children who were first-time omalizumab users	patients with severe allergic asthma		Any adverse events<br><br>Serious adverse events<br><br>Global Evaluation of Treatment Effectiveness (GETE)<br><br>Proportion of patients without asthma exacerbation-related events<br><br>Japanese Pediatric Asthma Control Program (JPAC) scores	47.2%<br><br>23.6%<br><br>'Effective' patients 77.2%:<br>-excellent 41.7%<br>-good 35.4%<br>'Not effective' patients 23.8%.<br><br>Higher for post-treatment with omalizumab than pre-treatment:<br>-Worsening of asthma symptoms requiring systemic steroid: 25.2% vs 74%.<br>-Frequency of hospitalization: 54% vs 85%.<br>-Visits to the emergency room: 43.6% vs 78.2%.<br>-Absence from school: 36.4% vs 78.2%.<br><br>Percentage of patients "completely controlled" (score 15) increased from 8.6% to 48.6%.<br>Percentage of patients "adequately controlled" (score 15) increased from 12.1% to 24.1%.<br>Percentage of patients "adequately controlled" (score 12-14) increased from 12.1% to 24.1%.<br>Percentage of patients "poorly controlled" (score ≤ 11) decreased from 79.3% to 27.6%.	
Real-life omalizumab exposure and discontinuatio n in a large	Real-life prospective study	Omalizumab exposure and long-term discontinuation in patients from	Patients with severe asthma<br><br>Regarding the exposure	2453 (6-18 years, subgroup of a total cohort of	Exposure group:<br>-Hospitalizations for asthma<br><br>-Use of OCS	 Decreased by 76.6%.<br><br>Decreased by 32.5%.	

nationwide population-based study of paediatric and adult asthma patients.<br>Humbert, 2022.<br>[11]		SNDS (National Health Data System)<br>database, with >10 years follow-up.	group, data were reported from the year preceding omalizumab initiation (T_{-12}/T_0) to 2 years after T_0 (T_{24})<br><br>Regarding the discontinuation group, data were reported from the year preceding omalizumab discontinuation (T_{-12}/T_{stop}) to 2 years after T_{stop} (T_{24}).<br>Two subgroup in this group: subgroup 1 with uncontrolled asthma patients at T_{stop} vs. subgroup 2 with controlled asthma patients at T_{stop}.	19203 patients)	-Use of ICS<br><br>Discontinuation group:<br>- Hospitalizations for asthma<br><br>-Use of OCS<br><br>-Use of ICS	Decreased by 11.6%.<br><br>For both subgroups, lower than before omalizumab treatment initiation (33.7% at T_0).<br>Subgroup 1: 18.4% before T_{stop}, 6.7% at 2 years.<br>Subgroup 2: 0 before T_{stop}, 0.6% at 2 years.<br><br>For both subgroups, lower than before omalizumab treatment initiation (73.2% at T_0).<br>Subgroup 1: 70.9% of patients before T_{stop}, 57% at 2 years.<br>Subgroup 2: 20.2% of patients before T_{stop}, 24.6% at 2 years.<br><br>For both subgroups, lower than before omalizumab treatment initiation (89.3% at T_0).<br>Subgroup 1: 84.9% of patients before T_{stop}, 73.3% at 2 years.<br>Subgroup 2: 60.8% of patients before T_{stop}, 53.5% at 2 years.	

Mepolizumab for urban children with exacerbation-prone eosinophilic asthma in the USA (MUPPITS-2): a randomised, double-blind, placebo-controlled, parallel-group trial. Jackson, 2022 [12]	Randomised, double-blind, placebo controlled, parallel-group trial.	Patients were randomly assigned 1:1 to mepolizumab (6-11 years: 40 mg; 12-17 years: 100 mg) or placebo injections once every 4 weeks for 52 weeks with the aim of defining whether mepolizumab, added to guidelines-based care, reduced the number of asthma exacerbations compared with guidelines-care alone.	Children and adolescents with exacerbation-prone asthma (defined as ≥ 2 exacerbations in the previous year) and blood eosinophil of at least 150 cells/μl	290 (6-17 years): 146 assigned to mepolizumab and 144 to placebo	Number of asthma exacerbations treated with systemic corticosteroids<br><br>Time to first asthma exacerbations<br><br>Adverse events<br><br>Blood eosinophil counts	It was 0.96 with mepolizumab vs 1.30 with placebo.<br><br>Not significantly different between treatment groups.<br><br>Modest between-group differences, except for higher rates of injection-site reactions associated with mepolizumab than with placebo.<br>Treatment-emergent adverse events occurred in 29% of patients in the mepolizumab group and in 11% of participants in the placebo group.<br><br>At the end were significantly reduced in the mepolizumab group with a different from the baseline of -299, but remained unchanged in the placebo group.	95% CI, 0.78-1.17<br>95% CI, 1.08-1.57<br>(rate ratio 0.73, *p* = 0.027)<br><br>HR 0.86 (95% CI, 0.063-1.18)<br><br>95% CI, -363 to -235; *p*<0.0001

CI: Confidence Interval, EOS: eosinophils, GC: glucocorticoid, FEV1: Forced Expiratory Flow in 1 second, FENO: Fractioned exhaled nitric oxide, ACQ-5 score: Asthma Control Questionnaire-5 score, OR: Odds Ratio, ACT: Asthma Control Test, LS: Least Squares, SGRQ: St George' s Respiratory Questionnaire, LABA: long-acting β-2 bronchodilator, ACQ-7 score: Asthma Control Questionnaire-7 score, C-ACT score: Childhood Asthma Control

Test, RR: Relative Risk, ACQ-7-IA Score: Asthma Control Questionnaire-7 Interviewer-Administered score, GETE: Global Evaluation of Treatment Effectiveness, HRCU: Healthcare Resource Use, OCS: oral corticosteroids, ICS: inhaled corticosteroids, IV: intravenously, SC: subcutaneously.

References

1. Humbert M, Taillé C, Mala L, Le Gros V, Just J, Molimard M; STELLAIR investigators. Omalizumab effectiveness in patients with severe allergic asthma according to blood eosinophil count: the STELLAIR study. Eur Respir J. 2018 May 10;51(5):1702523. doi: 10.1183/13993003.02523-2017. PMID: 29545284; PMCID: PMC6383600.

2. Casale TB, Luskin AT, Busse W, Zeiger RS, Trzaskoma B, Yang M, Griffin NM, Chipps BE. Omalizumab Effectiveness by Biomarker Status in Patients with Asthma: Evidence From PROSPERO, A Prospective Real-World Study. J Allergy Clin Immunol Pract. 2019 Jan;7(1):156-164.e1. doi: 10.1016/j.jaip.2018.04.043. Epub 2018 May 22. PMID: 29800752.

3. Chapman KR, Albers FC, Chipps B, Muñoz X, Devouassoux G, Bergna M, Galkin D, Azmi J, Mouneimne D, Price RG, Liu MC. The clinical benefit of mepolizumab replacing omalizumab in uncontrolled severe eosinophilic asthma. Allergy. 2019 Sep;74(9):1716-1726. doi: 10.1111/all.13850. Epub 2019 Jul 1. PMID: 31049972; PMCID: PMC6790683.

4. Folqué MM, Lozano J, Riggioni C, Piquer M, Álvaro M, Machinena A, Giner MT, Domínguez O, Jiménez-Feijoo RM, Dias da Costa M, Plaza AM. 'Real-life' experience in asthmatic children treated with omalizumab up to six-years follow-up. Allergol Immunopathol (Madr). 2019 Jul-Aug;47(4):336-341. doi: 10.1016/j.aller.2018.09.009. Epub 2018 Nov 30. PMID: 30509559.

5. Giubergia V, Ramírez Farías MJ, Pérez V, Crespi N, Castaños C. Clinical impact of omalizumab treatment in children with severe asthma: Report of a local experience. Arch Argent Pediatr. 2019 Apr 1;117(2):e115-e120. English, Spanish. doi: 10.5546/aap.2019.eng.e115. PMID: 30869489.

6. Gupta A, Ikeda M, Geng B, Azmi J, Price RG, Bradford ES, Yancey SW, Steinfeld J. Long-term safety and pharmacodynamics of mepolizumab in children with severe asthma with an eosinophilic phenotype. J Allergy Clin Immunol. 2019 Nov;144(5):1336-1342.e7. doi: 10.1016/j.jaci.2019.08.005. Epub 2019 Aug 16. PMID: 31425781.

7. Gupta A, Pouliquen I, Austin D, Price RG, Kempsford R, Steinfeld J, Bradford ES, Yancey SW. Subcutaneous mepolizumab in children aged 6 to 11 years with severe eosinophilic asthma. Pediatr Pulmonol. 2019 Dec;54(12):1957-1967. doi: 10.1002/ppul.24508. Epub 2019 Sep 9. PMID: 31502421; PMCID: PMC6972599.

8. Bacharier LB, Maspero JF, Katelaris CH, Fiocchi AG, Gagnon R, de Mir I, Jain N, Sher LD, Mao X, Liu D, Zhang Y, Khan AH, Kapoor U, Khokhar FA, Rowe PJ, Deniz Y, Ruddy M, Laws E, Patel N, Weinreich DM, Yancopoulos GD, Amin N, Mannent LP, Lederer DJ, Hardin M; Liberty Asthma VOYAGE Investigators. Dupilumab in Children with Uncontrolled Moderate-to-Severe Asthma. N Engl J Med. 2021 Dec 9;385(24):2230-2240. doi: 10.1056/NEJMoa2106567. PMID: 34879449.

9. Nieto García A, Garriga-Baraut T, Plaza Martín AM, Nieto Cid M, Torres Borrego J, Folqué Giménez MDM, Lozano Blasco J, Bosque García M, Moreno-Galarraga L, Tortajada-Girbés M, Rivas Juesas C, Penín Antón M, Caballero-Rabasco MA, Gaboli M, López Neyra A, Navarro Morón J, Freixa Benavente A, Valdesoiro Navarrete L, Ballester Asensio E, Sanz Santiago V, Romero García R, Gimeno Díaz de Atauri Á, Valenzuela Soria A, Sánchez Mateos M, Batlles Garrido J, Andrés Martín A, Campos Alonso E, Aragón Fernández C, Vázquez Rodríguez E, Martínez Pardo L, Del-Río Camacho G, Mazón Ramos Á. Omalizumab outcomes for up to 6 years in pediatric patients with severe persistent allergic asthma. Pediatr Allergy Immunol. 2021 Jul;32(5):980-991. doi: 10.1111/pai.13484. Epub 2021 Mar 20. PMID: 33619748.

10. Nakamura N, Kashitani Y, Yoshisue H, Nagasaki M, Sasajima T. Real-life long-term safety and effectiveness of omalizumab in Japanese pediatric patients with severe allergic asthma: A post-marketing surveillance. Allergol Int. 2021 Jul;70(3):319-326. doi: 10.1016/j.alit.2021.01.001. Epub 2021 Jan 30. PMID: 33526351.

11. Humbert M, Bourdin A, Taillé C, Kamar D, Thonnelier C, Lajoinie A, Rigault A, Deschildre A, Molimard M. Real-life omalizumab exposure and discontinuation in a large nationwide population-based study of paediatric and adult asthma patients. Eur Respir J. 2022 Nov 10;60(5):2103130. doi: 10.1183/13993003.03130-2021. PMID: 35618272; PMCID: PMC9647070.

12. Jackson DJ, Bacharier LB, Gergen PJ, Gagalis L, Calatroni A, Wellford S, et al. Mepolizumab for urban children with exacerbation-prone eosinophilic asthma in the USA (MUPPITS-2): a randomised, double-blind, placebo-controlled, parallel-group trial. Lancet 2022;400:502-11

PICO question 9. In children with asthma, does vitamin D supplementation help with asthma control?

Patient or population: children and adolescents with asthma

Setting: primary to tertiary care

Intervention: vitamin D supplementation

Comparison: no vitamin D supplementation

Outcome: asthma control

Search strategy:

(("Vitamin D"[Mesh]) AND "Asthma"[Mesh]) AND "Child"[Mesh]

Filters applied: last 5 years, Child: 6-12 years, Adolescent: 13-18 years

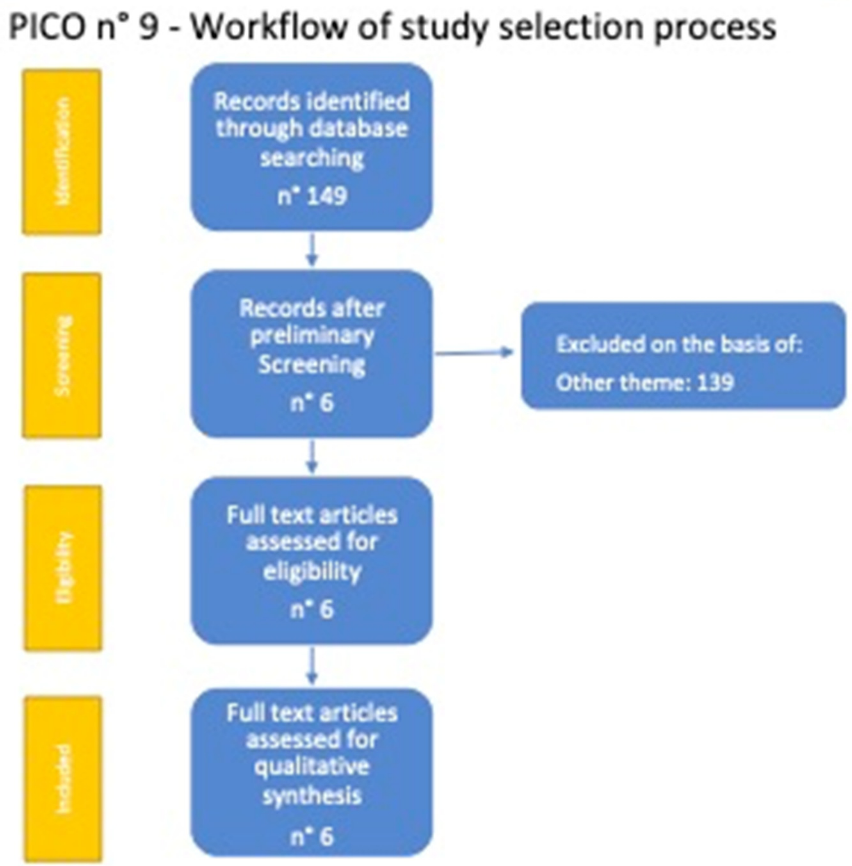

Modified from: Moher D, Liberati A, Tetzlaff J, Altman DG, The PRISMA Group (2009). Preferred Reporting Items for Systematic Reviews and Meta-Analyses: The PRISMA Statement. PLoS Med 6(7): e1000097

Title of the study, first author, year	Type of study	Study design	Population	N° of patients, age	Methods	Outcomes	
Association between serum Vitamin D levels and asthma severity and control in children and adolescents. Malheiro APG, 2023 [1]	Longitudinal and prospective study	Assessment the association of serum Vitamin D (vitD) levels with asthma control and severity in children and adolescents in different seasons of the year	children and adolescents diagnosed with asthma underwent two assessments conducted in opposite seasons of the year which included a clinical assessment, a questionnaire for classification of asthma control (Asthma Control Test), spirometry, and blood collection to measure serum vitD levels	141, 7-17 years of age	serum Vitamin D (vitD) levels<br><br>Asthma control	The mean vitD was lower in females and sunlight exposure appears not to be an influencing factor for vitD levels.<br><br>no differences in mean vitD of patients with controlled and uncontrolled asthma<br><br>Severe asthma group had lower mean Vitamin D than the mild/moderate asthma group for both assessments. The group with vitD insufficiency had a higher prevalence of severe asthma Vitamin D was positively correlated with FEV_1 in both assessments and with $FEF_{25-75\%}$ in the first assessment.	
Effect of vitamin D on lung function assessed by forced oscillation technique in asthmatic children with vitamin D deficiency: A	randomized double-blind placebo-controlled trial	Evaluation if vitamin D treatment would improve lung function assessed by forced oscillation technique (FOT) in	Asthmatic children aged 3-18 years who had controlled asthma according to GINA guideline for a least 1 month. Ninety two asthmatic children	97, 3-18 years of age	Level of asthma control using pediatric asthma control test (P-ACT) for patients aged 4–11 years or the asthma control test (ACT) for	no significant differences	

randomized double-blind placebo-controlled trial. Swangtrakul, 2022 [2]		vitamin D deficient asthmatic children	were recruited: 41 children (44.6%) had total 25(OH)D < 20 ng/ml (VDD) and 51 children had total 25(OH)D ≥ 20 ng/ml (nVDD): 40 children (43.5%) had total 25(OH)D 21-30 ng/ml and 11 children (12%) had total 25(OH)D > 30 ng/ml. Forty one children in VDD group were randomized: 21 children in VDD group with treatment (tVDD), and 20 children in VDD group with placebo (pVDD).		patients aged 12–15 years.<br><br>Forced Oscillation Technique (FOT) parameters includ-ing respiratory resistance at 5 Hz (R5), respiratory resistance at 20 Hz (R20), and respiratory reactance at 5 Hz (X5), area of reactance (ALX), resonance frequency (Fres) and the percentage of the predicted (% predicted) of FOT values were re-corded.<br><br>Serum concentrations of 25(OH) vitamin D	The decrease of FOT parameters was observed in all groups but the significant changes of R5 and R20 were demonstrated only in nVDD and pVDD groups. The percentage changes of R5 and R20 from baseline values at 1 and 3 months were not significantly different among nVDD, tVDD and pVDD.<br><br>There was no significant correlation between serum total 25 (OH)D level and % predicted of FOT parameter.	
Vitamin D supplementation, lung function, and asthma control in children with asthma and low vitamin D levels Yueh-Ying Han, 2021 [3]	randomized, double-blind, parallel, placebo-controlled clinical trial	Evaluation if high-dose vitamin D supplementation would improve lung function, asthma control, and asthma-related quality of life in children with asthma and vitamin D levels below 30 ng/ml.	Eligible participants were children with asthma, aged 6 to 16 years, with serum vitamin D levels <30 ng/mL but ≥ 10 ng/mL (until July 21, 2017) or ≥14 ng/mL.	192, 6-16 years of age	Percent predicted lung function measures (FEV1, FVC, or FEV1/FVC), asthma control, or asthma-related quality of life	Vitamin D supplementation, compared with placebo, had no significant effect.	These results do not support recommending vitamin D supplementation to improve lung function, asthma control, or asthma-related quality of life in this population.

		Each participant was randomly assigned to either daily placebo capsules or daily vitamin D3, 4,000 IU, plus inhaled fluticasone propionate (88 µg twice per day in children aged 6–11 years and 110 µg twice per day in children ≥12 years).					
Vitamin - D supplementation as an adjunct to standard treatment of asthma in children: A randomized controlled trial (ViDASTA Trial). Chirag Thakur, 2021 [4]	Randomized controlled trial	Evaluation of the role of vitamin D supplementation as an adjunct to standard treatment in childhood asthma	Children aged 6 to 11 years with first time diagnosed moderate persistent asthma and randomly assigned them into intervention (2000 IU per day of vitamin D) and placebo groups (n = 30 each)	60, 6-11 years of age	Primary outcome: asthma control as assessed by the childhood asthma control test (C - ACT) scores at 12 weeks post - randomization<br><br>Secondary outcomes: improvement in the forced expiration in 1 s (FEV1), fractional exhaled nitric oxide (FeNO), asthma exacerbations, use of systemic steroids, number of emergency visits, post - intervention vitamin D levels, and adverse outcomes.	no significant difference between the C - ACT score in the two groups<br><br>no significant difference	median [first–third quartile] scores were 25 [24–26] in both groups, p = 0.719<br><br>p of FEV1 mean: 0.2<br><br>p of FeNO: 0.2<br><br>p of no. of patients with exacerbation: 0.3<br><br>p of use of systematic steroids: 0.3

Efficacy of vitamin D supplementation in asthmatic children with vitamin D deficiency: A randomized controlled trial (ESDAC trial). Kana Ram Jat, 2020 [5]	Randomized controlled trial	Evaluation of the Efficacy and Safety of vitamin D supplementation as compared to placebo supplementation in Asthmatic Children who were vitamin D deficient- the ESDAC trial	Asthmatic children of 4-12 years of age who had 25-hydroxyvitamin D [25(OH)D] levels <20 ng/mL. The participants were randomized to receive either vitamin D orally 1000 IU/d for 9 months or similar-looking placebo.	250 children (125 in each group), 4 to 12 years of age	Primary outcome: the proportion of children having Childhood Asthma Control Test (CACT) score15 of ≥ 20 or more at the end of the intervention and any adverse effects	The proportion of children with CACT scores ≥ 20 increased significantly from baseline to end of the study in both vitamin D and placebo groups, but there was no difference between the groups at the end of study. The adverse effects were not different between two groups.	93.6% vs 92.0%; p-value 0.625
					Secondary outcomes: change in forced expiratory volume in the first second (FEV1), FEV1/FVC, and PEFR from baseline to end of the treatment	Mean values of lung function parameters at the end of study and change from baseline to end of the study were not different between the groups except that mean FVC was significantly more in placebo at the end of the study	
					change in mean CACT score from baseline to end of the treatment	No difference in CACT score and in median (IQR) change in CACT score from baseline to end of the study	23.1±4.4 vs 22.7±3.7; p 0.743
					number of emergency visits during study period, number of days requiring rescue medications and the number of night awakenings,	no difference between the groups	8 (5, 9) vs 7.5 (5, 9.5); p-value 0.914

					height gain from enrolment to end of the study, the number of courses of oral steroids uses, total dose of inhaled steroids, change in 25(OH)D levels		
Effect of Vitamin D3 Supplementation on Severe Asthma Exacerbations in Children With Asthma and Low Vitamin D Levels: The VDKA Randomized Clinical Trial. Forno, 2020 [6]	Randomized Clinical Trial	The Vitamin D to Prevent Severe Asthma Exacerbations (VDKA) Study was a randomized, double-blind, placebo-controlled clinical trial of vitamin D3 supplementation to improve the time to severe exacerbations in high-risk children with asthma aged 6 to 16 years taking low-dose inhaled corticosteroids and with serum 25-hydroxyvitamin D levels less than 30 ng/mL.	High-risk children with asthma, aged 6 to 16 years, with serum vitamin D levels less than 30 ng/mL but greater than or equal to 10 ng/mL or greater than or equal to 14 ng/mL	192, 6 to 16 years of age	Primary outcome: time to a severe asthma exacerbation during the 48-week trial period<br><br>Secondary outcomes: the time to a viral-induced severe asthma exacerbation<br><br>the ability to reduce the dose of inhaled steroids by50%at the 24-week study visit	VitaminD3 supplementation did not significantly prolong the time to a severe asthma exacerbation<br><br>VitaminD3 supplementation did not significantly prolong the time to a first viral-induced severe exacerbation compared with placebo<br><br>not significantly different between the vitamin D3 and the placebo groups<br><br>No significant reduction in	Mean group difference of -13.1 days (95% CI, −42.6 to 16.4) and adjusted HR of 1.13 (95% CI, 0.69-1.85; *P* = .63)<br><br>mean group difference of −9.1 days (95% CI, −35.5 to 17.2) and an adjusted HR of 1.32 (95% CI, 0.63-2.75; *P* = .46)<br><br>(group difference, −1.1 % [95%CI, −14.6% to 12.4%]; adjusted relative risk ratio, 0.99 [95% CI, 0.66-1.52]; *P* = .99)

					the cumulative dose of inhaled steroids during the study period.	the cumulative dose of fluticasone during the trial	mean group difference, 4.41 mg (95% CI, − 0.99 to 9.80); adjusted mean difference, 4.40 mg (95% CI, 0.001 to 8.80); *P* = .049

References

1. Malheiro APG, Gianfrancesco L, Nogueira RJN, Grotta MB, Morcillo AM, Ribeiro JD, Toro AADC. Association between serum Vitamin D levels and asthma severity and control in children and adolescents. Lung. 2023 Apr;201(2):181-187. doi: 10.1007/s00408-023-00605-x. Epub 2023 Feb 21. PMID: 36809416.
2. Swangtrakul N, Manuyakorn W, Mahachoklertwattana P, Kiewngam P, Sasisakulporn C, Jotikasthirapa W, Kamchaisatian W, Benjaponpitak S. Effect of vitamin D on lung function assessed by forced oscillation technique in asthmatic children with vitamin D deficiency: A randomized double-blind placebo-controlled trial. Asian Pac J Allergy Immunol. 2022 Mar;40(1):22-30. doi: 10.12932/AP-010519-0553. PMID: 31837209.
3. Han YY, Forno E, Bacharier LB, Phipatanakul W, Guilbert TW, Cabana MD, Ross K, Blatter J, Rosser FJ, Durrani S, Luther J, Wisniewski SR, Celedón JC. Vitamin D supplementation, lung function and asthma control in children with asthma and low vitamin D levels. Eur Respir J. 2021 Oct 28;58(4):2100989. doi: 10.1183/13993003.00989-2021. PMID: 34326185; PMCID: PMC8556333.
4. Thakur C, Kumar J, Kumar P, Goyal JP, Singh K, Gupta A. Vitamin-D supplementation as an adjunct to standard treatment of asthma in children: A randomized controlled trial (ViDASTA Trial). Pediatr Pulmonol. 2021 Jun;56(6):1427-1433. doi: 10.1002/ppul.25287. Epub 2021 Feb 1. PMID: 33522698.

5. Jat KR, Goel N, Gupta N, Gupta CP, Datta S, Lodha R, Kabra SK. Efficacy of vitamin D supplementation in asthmatic children with vitamin D deficiency: A randomized controlled trial (ESDAC trial). Pediatr Allergy Immunol. 2021 Apr;32(3):479-488. doi: 10.1111/pai.13415. Epub 2020 Dec 4. PMID: 33207014.
6. Forno E, Bacharier LB, Phipatanakul W, Guilbert TW, Cabana MD, Ross K, Covar R, Gern JE, Rosser FJ, Blatter J, Durrani S, Han YY, Wisniewski SR, Celedón JC. Effect of Vitamin D3 Supplementation on Severe Asthma Exacerbations in Children With Asthma and Low Vitamin D Levels: The VDKA Randomized Clinical Trial. JAMA. 2020 Aug 25;324(8):752-760. doi: 10.1001/jama.2020.12384. Erratum in: JAMA. 2021 Jul 6;326(1):90. PMID: 32840597; PMCID: PMC7448830.

PICO question 10. In children with asthma, does flu vaccination help with asthma control?

Patient or population: children and adolescents with asthma

Setting: primary to tertiary care

Intervention: flu vaccine

Comparison: no flu vaccine

Outcome: asthma control

Search strategy:

(("Influenza Vaccines"[Mesh]) AND "Asthma"[Mesh]) AND "Child"[Mesh]

Filters applied: last 5 years, Child: 6-12 years, Adolescent: 13-18 years

PICO n° 10 - Workflow of study selection process

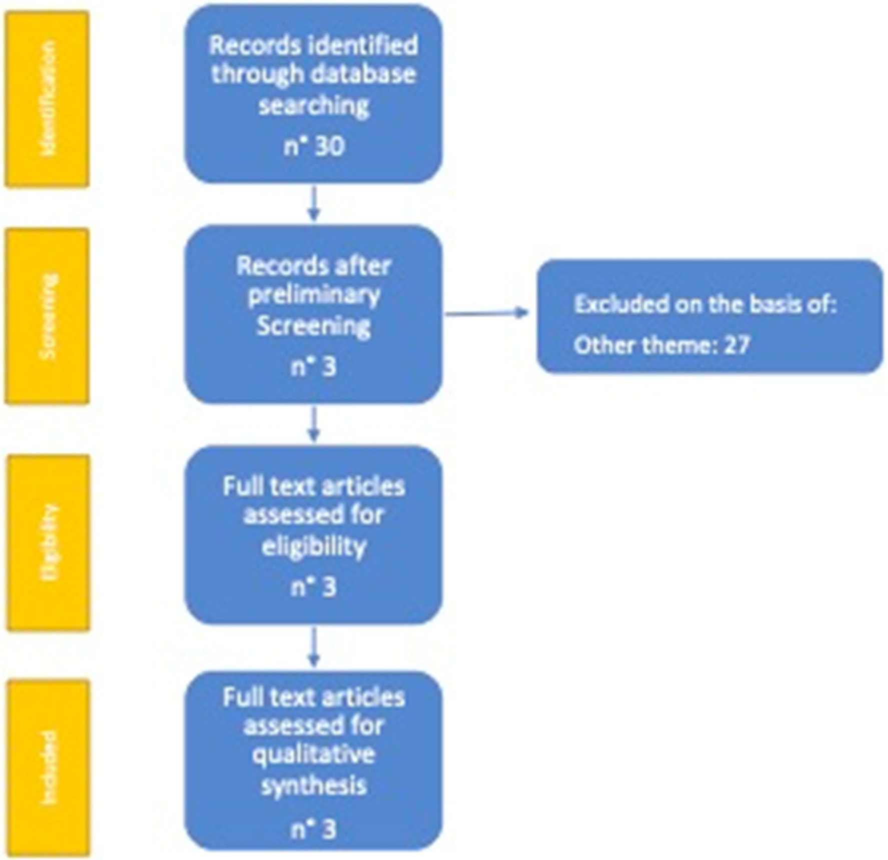

Modified from: Moher D, Liberati A, Tetzlaff J, Altman DG, The PRISMA Group (2009). Preferred Reporting Items for Systematic Reviews and Meta-Analyses: The PRISMA Statement. PLoS Med 6(7): e1000097

Title of the study, first author, year	Type of study	Study design	Population	N° of patients, age	Outcomes	Results	
Safety of Live Attenuated Influenza Vaccine in Children With Asthma. Sokolow, 2022 [1]	Randomized Controlled Trial	Safety of quadrivalent live attenuated abstract influenza vaccine (LAIV4) in children with asthma, comparing the proportion of children with asthma exacerbations after LAIV4 or quadrivalent inactivated influenza vaccine (IIV4)	Children 5 to 17 years of age with a current diagnosis of persistent asthma. Participants were randomly assigned 1:1 to receive either a single intranasal dose of LAIV4 (FluMistVR Quadrivalent, AstraZeneca) or an intramuscular injection of IIV4 (FluzoneVR Quadrivalent Vaccine, Sanofi Pasteur).	151 children with asthma, aged 5 to 17 years	Primary outcome: asthma exacerbation (any acute episode of progressively worsening shortness of breath, cough, wheezing, chest tightness, and/or respiratory distress during the 42 days after influenza vaccination)<br><br>Secondary outcomes: frequency of asthma symptoms, change in PEFR, or childhood asthma control test/asthma control test scores in the 14 days postvaccination between LAIV4 and IIV4 recipients.<br><br>Vaccine reactogenicity	18 of 142 (12.7%) of participants experienced an asthma exacerbation: 8 of 74 in the LAIV4 group (10.8%) versus 10 of 68 in the IIV4 group (14.7%)<br><br>no significant differences<br><br>similar between groups, although sore throat (P=.051) and myalgia (P<.001) were more common in the IIV4 group	The risk difference of LAIV4 and IIV4 was -0.0390 (90% confidence interval: -0.1453 to 0.0674)
Increasing rates of influenza vaccination were associated with lower asthma prevalence in United States	Letter	Evaluation whether asthma is associated with increased influenza vaccination and whether influenza vaccination trends	Cohorts were stratified by age (0 − 5/6 − 10/11 − 17 years), personal history of atopic disease (yes[high risk of asthma]/no	124,569, 0 to 17 years of age		There were significant interactions between influenza vaccination and year as predictors for current asthma at ages 6 − 10 (both: $p < .0001$) and 11 − 17 years (both: $p < .0001$) in	

children. Hou, 2021 [2]		contributed to trends of asthma prevalence in US children	[low risk of asthma]), and parental history of atopic disease (yes/no)			linear and spline models, but not at ages 0 − 5 years (linear: *p* = .153, spline: *p* = .0901).<br><br>There were greater decreases in the odds of current asthma prevalence among children who were vaccinated (all ages, 6 − 10, 11 − 17 years) compared with those who were not vaccinated for influenza (all ages, 6 − 10, 11 − 17 years) from 2005 − 2006 to 2017 − 2018<br><br>Similar results were observed in those with or without a personal or family history of atopic disease	
Effect of influenza vaccination in patients with asthma. Martinez-Baz, 2021 [3]	test-negative case-control study	Effect of influenza vaccination in the current and previous seasons in preventing influenza among people with asthma	Patients with asthma who were in hospital or seen in primary health care centres for influenza-like illness and were tested for influenza virus using RT-PCR. We were blinded to vaccination status and test results for patients during the inclusion process	1032, >9 years of age	Influenza vaccination effect in patients with asthma	The overall protective effect against influenza was 43% in patients who were vaccinated in the current season regardless of vaccination in previous seasons, and 38% in patients who were vaccinated in previous seasons but not the current one, compared with those with asthma who were not vaccinated in the current and 5 previous seasons.<br><br>The odds of having influenza between people with and without asthma did not differ significantly among those who were not vaccinated, among people vaccinated in the current season or among	OR 0.57, 95% CI 0.40 to 0.80<br><br>OR 0.62, 95%CI 0.39 to 0.96

					Vaccination effect in persons with and without asthma	people vaccinated in previous seasons only	(OR 1.16, 95% CI 0.89 to 1.51)<br><br>(OR 1.12, 95% CI 0.91 to 1.38)<br><br>(OR 1.05, 95% CI 0.71 to 1.55)

References

1. Sokolow AG, Stallings AP, Kercsmar C, Harrington T, Jimenez-Truque N, Zhu Y, Sokolow K, Moody MA, Schlaudecker EP, Walter EB, Staat MA, Broder KR, Creech CB. Safety of Live Attenuated Influenza Vaccine in Children With Asthma. Pediatrics. 2022 Apr 1;149(4):e2021055432. doi: 10.1542/peds.2021-055432. Erratum in: Pediatrics. 2022 Aug 1;150(2): PMID: 35342923.
2. Hou A, Silverberg JI. Increasing rates of influenza vaccination were associated with lower asthma prevalence in United States children. Allergy. 2021 Jul;76(7):2273-2275. doi: 10.1111/all.14790. Epub 2021 Mar 15. PMID: 33624361.
3. Martínez-Baz I, Navascués A, Casado I, Portillo ME, Guevara M, Gómez-Ibáñez C, Burgui C, Ezpeleta C, Castilla J. Effect of influenza vaccination in patients with asthma. CMAJ. 2021 Jul 26;193(29):E1120-E1128. doi: 10.1503/cmaj.201757. PMID: 34312165; PMCID: PMC8321300.
